# Supplementary material for: Mediator or moderator? The role of obesity in the association between age at menarche and blood pressure in middle-aged and elderly Chinese: a population-based cross-sectional study
Source: BMJ Open. 2022 May 25;12(5):e051486. doi: 10.1136/bmjopen-2021-051486 (PMC9137347; doi:10.1136/bmjopen-2021-051486)
Supplement: Author's manuscript [file bmjopen-2021-051486.draft_revisions.pdf]

BMJ Open is committed to open peer review. As part of this commitment we make the peer review history of every article we publish publicly available.

When an article is published we post the peer reviewers' comments and the authors' responses online. We also post the versions of the paper that were used during peer review. These are the versions that the peer review comments apply to.

The versions of the paper that follow are the versions that were submitted during the peer review process. They are not the versions of record or the final published versions. They should not be cited or distributed as the published version of this manuscript.

BMJ Open is an open access journal and the full, final, typeset and author-corrected version of record of the manuscript is available on our site with no access controls, subscription charges or pay-per-view fees (http://bmjopen.bmj.com).

If you have any questions on BMJ Open's open peer review process please email
info.bmjopen@bmj.com

BMJ Open

Association between age at menarche and blood pressure in mid-aged and elderly Chinese: is obesity an important mediator or moderator?

Journal:	*BMJ Open*
Manuscript ID	bmjopen-2021-051486
Article Type:	Original research
Date Submitted by the Author:	21-Mar-2021

Complete List of Authors:	zhang, lin; Wannan Medical College, Department of Internal Medicine Nursing, School of Nursing, Wannan Medical College, 22 Wenchang West Road, Higher Education Park, Wuhu City, An Hui Province, P.R.China<br>Yang, Liu; Department of Internal Medicine Nursing, School of Nursing, Wannan Medical College, 22 Wenchang West Road, Higher Education Park, Wuhu City, An Hui Province, P.R.China<br>Wang, Congzhi; Wannan Medical College, Department of Internal Medicine Nursing, School of Nursing, Wannan Medical College, 22 Wenchang West Road, Higher Education Park, Wuhu City, An Hui Province, P.R.China<br>Yuan, Ting; Wannan Medical College, Obstetrics and Gynecology Nursing, School of Nursing, Wannan Medical College, 22 Wenchang West Road, Higher Education Park, Wuhu City, An Hui Province, P.R.China<br>Zhang, Dongmei; Wannan Medical College, Department of Pediatric Nursing, School of Nursing, Wannan Medical College, 22 Wenchang West Road, Higher Education Park, Wuhu City, An Hui Province, P.R.China<br>Wei, Huanhuan; Wannan Medical College, Obstetrics and Gynecology Nursing, School of Nursing, Wannan Medical College, 22 Wenchang West Road, Higher Education Park, Wuhu City, An Hui Province, P.R.China<br>Li, Jing; Wannan Medical College, Department of Surgical Nursing, School of Nursing, Wannan Medical College, 22 Wenchang West Road, Higher Education Park, Wuhu City, An Hui Province, P.R.China<br>Lei, Yunxiao; Wannan Medical College, Obstetrics and Gynecology Nursing, School of Nursing, Wannan Medical College, 22 Wenchang West Road, Higher Education Park, Wuhu City, An Hui Province, P.R.China; Henan University of Science and Technology, School of Nursing<br>Sun, Lu; Wannan Medical College, Department of Emergency and Critical Care Nursing, School of Nursing, Wannan Medical College, 22 Wenchang West Road, Higher Education Park, Wuhu City, An Hui Province, P.R.China<br>Li, Xiaoping; Wannan Medical College, Department of Emergency and Critical Care Nursing, School of Nursing, Wannan Medical College, 22 Wenchang West Road, Higher Education Park, Wuhu City, An Hui Province, P.R.China<br>Hua, Ying; Wannan Medical College, Rehabilitation Nursing, School of Nursing, Wanna Medical college, 22 Wenchang West Road, Higher Education Park, Wuhu City, An Hui Province, P.R.China<br>Che, Hengying; Yijishan Hospital of Wannan Medical College, Department of nursing, Yijishan Hospital, the First Affiliated Hospital of Wannan

Medical College, Zheshan West Road, Yijishan District, Wuhu City, Anhui Province
Li, Yuanzhen; Wannan Medical College, Department of Emergency and Critical Care Nursing, School of Nursing, Wannan Medical College, 22 Wenchang West Road, Higher Education Park, Wuhu City, An Hui Province, P.R.China

Keywords: Hypertension < CARDIOLOGY, Risk management < HEALTH SERVICES ADMINISTRATION & MANAGEMENT, Nutrition < TROPICAL MEDICINE

SCHOLARONE™ Manuscripts

I, the Submitting Author has the right to grant and does grant on behalf of all authors of the Work (as defined in the below author licence), an exclusive licence and/or a non-exclusive licence for contributions from authors who are: i) UK Crown employees; ii) where BMJ has agreed a CC-BY licence shall apply, and/or iii) in accordance with the terms applicable for US Federal Government officers or employees acting as part of their official duties; on a worldwide, perpetual, irrevocable, royalty-free basis to BMJ Publishing Group Ltd ("BMJ") its licensees and where the relevant Journal is co-owned by BMJ to the co-owners of the Journal, to publish the Work in this journal and any other BMJ products and to exploit all rights, as set out in our licence.

The Submitting Author accepts and understands that any supply made under these terms is made by BMJ to the Submitting Author unless you are acting as an employee on behalf of your employer or a postgraduate student of an affiliated institution which is paying any applicable article publishing charge ("APC") for Open Access articles. Where the Submitting Author wishes to make the Work available on an Open Access basis (and intends to pay the relevant APC), the terms of reuse of such Open Access shall be governed by a Creative Commons licence – details of these licences and which Creative Commons licence will apply to this Work are set out in our licence referred to above.

Other than as permitted in any relevant BMJ Author's Self Archiving Policies, I confirm this Work has not been accepted for publication elsewhere, is not being considered for publication elsewhere and does not duplicate material already published. I confirm all authors consent to publication of this Work and authorise the granting of this licence.

For peer review only

Association between age at menarche and blood pressure in mid-aged and elderly Chinese: is obesity an important mediator or moderator?

Lin Zhang[1], Liu Yang[1], Congzhi Wang[1], Ting Yuan[2], Dongmei Zhang[3], Huanhuan Wei[2], Jing Li[4], Yunxiao Lei[2], Lu Sun[5], Xiaoping Li[5], Ying Hua[6], Hengying Che[7*], Yuanzhen Li[5*]

*Correspondence: Hengying Che: 2722659079@qq.com and Yuanzhen Li: yuanzhen.li@wnmc.edu.cn

Yuanzhen Li and Hengying Che contributed equally to this work.

Lin Zhang: yaoran2008@163.com

Liu Yang: zqzqylyl5858@163.com

Congzhi Wang: 289957420@qq.com

Ting Yuan: yuanting@wnmc.edu.cn

Dongmei Zhang: dongmei.0715@163.com

Huanhuan Wei: whh0702@163.com

Jing Li: 475366616@qq.com

Yunxiao Lei: lyxheart@163.com

Lu Sun: 565196200@qq.com

Xiaoping Li：349274808@qq.com

Ying Hua: 1542997737@qq.com

Hengying Che: 2722659079@qq.com

Yuanzhen Li: yuanzhen.li@wnmc.edu.cn

[1] Department of Internal Medicine Nursing, School of Nursing, Wannan Medical College, 22 Wenchang West Road, Higher Education Park, Wuhu City, An Hui Province, P.R.China

[2] Obstetrics and Gynecology Nursing, School of Nursing, Wannan Medical College, 22 Wenchang West Road, Higher Education Park, Wuhu City, An Hui Province, P.R.China

[3] Department of Pediatric Nursing, School of Nursing, Wannan Medical College, 22 Wenchang West Road, Higher Education Park, Wuhu City, An Hui Province, P.R.China

[4] Department of Surgical Nursing, School of Nursing, Wannan Medical College, 22 Wenchang West Road, Higher Education Park, Wuhu City, An Hui Province, P.R.China

[5] Department of Emergency and Critical Care Nursing, School of Nursing, Wannan Medical College, 22 Wenchang West Road, Higher Education Park, Wuhu City, An Hui Province, P.R.China

[6] Rehabilitation Nursing, School of Nursing, Wanna Medical college, 22 Wenchang West Road, Higher Education Park, Wuhu City, An Hui Province, P.R.China

[7] Department of nursing, Yijishan Hospital, the First Affiliated Hospital of Wannan Medical College, Zheshan West Road, Yijishan District, Wuhu City, Anhui Province

For peer review only

For peer review only - http://bmjopen.bmj.com/site/about/guidelines.xhtml

ABSTRACT

Objective We investigated the moderation/mediation between the age of menarche and obesity parameters in predicting blood pressure (BP) in middle-aged and elderly Chinese.

Design Cross-sectional study.

Setting Data were selected from the China Health and Retirement Longitudinal Study.

Participants The analytic sample included 4513 Individuals aged 45 to 96.

Main outcome measurements Data were selected from the China Health and Retirement Longitudinal Study (CHARLS), a cross-sectional study. Differences between baseline characteristics were evaluated using a t-test. The trend of related variables by baseline characteristics was also tested using contrast analysis, as appropriate. Then, correlations between baseline characteristics, independent, moderators, mediators and dependent variables were used by Spearman's correlation test and Pearson's correlation test. Finally, the mediation analysis was performed by model 4 in process v3.3, and the moderation analysis was used model 1 to assessment. All covariates were adjusted in the moderation or mediation models.

Results In correlation analysis, BMI and WC levels were positively correlated with both SBP and DBP in women. age of menarche was negatively correlated with DBP. However, the age of menarche was not significantly correlated with SBP. In the moderator analysis, after controlling for the total potential confounders, the interaction term of obesity parameters*age of menarche was not significant for predicting either DBP or SBP. All correlations were significant correlation between age of menarche, obesity parameters and BP except the path of the menarche age →BP (with the addition of the BMI or WC indicator) in model one. In general, after controlling for potential confounders. WC and BMI partly mediated the relationship between age of menarche and BP.

Conclusion The interaction term of obesity parameters*age of menarche was not significant for predicting either DBP or SBP in women. Moreover, obesity parameters partly mediated the relationship between age of menarche and BP.

Keywords age at menarche, blood pressure, body mass index, moderator, mediator, obesity parameters, waist circle

Strengths and limitations of the study

This is the first large population study to examine the moderation between the age of menarche and obesity parameters in predicting blood pressure (BP) in middle-aged and elderly Chinese, as

For peer review only - http://bmjopen.bmj.com/site/about/guidelines.xhtml

well as the mediation effects of obesity parameters on the relationship between age of menarche and BP.

This study included a large sample of 4513 middle-aged and older Chinese. The results provided baseline data that could be explored further in future prospective studies.

The main limitations of our research are related to the cross-sectional study and the self-reported method used for the assessment of age at menarche and most related confounders.

For peer review only

For peer review only - http://bmjopen.bmj.com/site/about/guidelines.xhtml

INTRODUCTION

Blood pressure is the force of circulating blood against the walls of the body's arteries, the major blood vessels in the body. Hypertension is diagnosed if blood pressure readings are 140-90 or above on two different days. As a chronic disease[1], hypertension or elevated blood pressure (BP) is a severe medical condition that significantly increases stroke, heart attack, blindness, Kidney failure, and other complications. Hypertension risk factors[2-6] include stress, harmful use of alcohol, salt consumption, low intake of fruits and vegetables, being overweight or obese, saturated fat and trans fats, tobacco use, low diet in vitamin D, lack of physical activity, family history, aged 65 years or over and co-existing diseases. The World Health Organization(WHO) said[7] an estimated 1.13 billion people worldwide have hypertension and most (two-thirds) living in low- and middle-income countries. It means that most people have high blood pressure. Compared to the global average, people in low- and middle-income countries may bear a disproportionate and heavier burden of the disease, owing to several factors such as the ongoing nutritional transition in dietary consumption and energy expenditure, increasing trends in sedentary lifestyle, and other modifiable risk factors, and inadequate health care systems[8]. As a developing country, China has shown a relatively high and stable prevalence of hypertension among adult, especially in middle-aged and older people. Mahajan S, Zhang D, He S, et al. [9] showed the prevalence of hypertension among adults in China aged 45 and above was approximately 40.78% in 2011, 43.25% in 2013 and 41.81% in 2015. However, the proportion of hypertension control was still below 30%. Recently, hypertension control has become one of the critical public health intervention[10]. Therefore, it emphasizes the promotion of public health strategy to prevent the significant hypertension risk factors and assure the fullest available accessibility and use of quality health control for people with risk factors or who develop subclinical or overt hypertension. These actions are integral to a comprehensive public health strategy for hypertension promotion and prevention[11-13]. Though the etiology of hypertension is complex, one of the strongest risk factors was overweight/obesity. Thus, increased body mass index (BMI) or centrally located body fat (especially waist circle [WC]) increases the risk of developing hypertension.

Several studies[14-18]have reported that early biological maturation has been associated with obesity and blood pressure in adolescents. Werneck AO et al showed that behavioral and hereditary variables are more related to BP in late-maturing adolescents in adolescents. Widen E, et al[15] found that earlier pubertal timing associated with higher adult BMI and diastolic blood pressure in

For peer review only - http://bmjopen.bmj.com/site/about/guidelines.xhtml

both sexes. Mueller NT, et al [16] showed that earlier menarche was associated with higher waist circumference and BMI measured. Dreyfus J, et al[17] found that earlier age at menarche was associated with higher mean body mass index among African American and White women. Cao M, et al[18] showed that earlier age at menarche was associated with decreased diastolic blood pressure. However, it is not completed understood whether the changes in biological processes in the early years can affect cardiovascular outcomes across the life span. As Important indicators of obesity, BMI/WC is directly associated with biological maturation (based on menarche age). Evidence from several studies[19-21] indicated that menarche was related to BMI in later life. Power C, et al[19] found that timing of puberty was strongly associated with BMI for the earlier matures at ages 7-33 years. Adair LS, et al[20] showed that overweight prevalence rates were significantly higher in early maturing adolescents. Must A, et al[21] showed that early maturation in adult female overweight is mostly a result of the influence of elevated relative weight on early maturation. It has been speculated that the association between biological maturation and hypertension risk factors (based on menarche age) can be mediated or moderated by obesity parameters. Although speculative, the mediating or moderating role of obesity on the effect of early maturation (based on menarche age) on BP in adulthood has not been classified. However, this is especially important given that the onset of biological maturation, especially the age of menarche, now occurs at a much earlier age than in previous studies. Moreover, fully understanding the mediating effect of obesity on the association between early maturation (based on menarche age) and BP in adulthood could help identify women group that are at risk (based on obesity parameters) and those that should be put forward designed to place the many diverse conceptual and practice approaches and accomplishments in the early intervention. The moderating effect between early maturation (based on menarche age) and risk of hypertension (obesity parameters) on BP may also be fully considered.

To date, few studies on the mediating and moderating analysis between early maturation (based on menarche age) and risk of hypertension (obesity parameters) were conducted in individuals aged ≥45 years. Thus, this study aimed to determine their association with age of menarche, obesity parameters, and other confounding factors using the large national cohort study (CHARLS) from individuals aged ≥45 years in China.

For peer review only - http://bmjopen.bmj.com/site/about/guidelines.xhtml

METHODS

Study design and setting

Data from a longitudinal study named the China Health and Retirement Longitudinal Study (CHARLS) were used in our research. The CHARLS was a nationally representative longitudinal study conducted by the China Centre for Economic Research at Peking University[22] from 2011. In the 2011 CHARLS Wave1, at baseline, 6883 individuals were recruited for a longitudinal study, 41 individuals were excluded because of the absence of medication history, a group of 1875 participants did not have their metabolic measures, and 65 individuals did not have their blood pressure. Additionally, 389 Individuals were excluded from those who used antihypertension drugs. Finally, 4513 individuals were included in the analyses.

For peer review only

For peer review only - http://bmjopen.bmj.com/site/about/guidelines.xhtml

Individuals

The Individuals of the study were selected from the China Health and Retirement Longitudinal Study (CHARLS), Wave 1 (2011) [22]. The CHARLS involved 8779 individuals aged ≥45 years were total women [mean ± standard deviation age=58.59 ± 9.31years, ranged from 45 to 96years]. The mean and standard deviation of menarche age were 16.27±2.18 years (ranged from 10 to 30 years).

Self-report of risk factors

Baseline variables including age, age of menarche (0= ≤14years, 1= 15years, 2= 16years, 3= 17years, 4= ≥18years), educational levels (0= Illiterate, 1= Less than elementary school, 2= High school, 3= Above vocational school), marital status(0= Single, 1= Married), place of residence(0= Rural, 1= Urban), smoking habits(0= No, 1= Former smoke, 2= Current smoke), alcohol consumption (0= No, 1= Less than once a month, 2= More than once a month), eating meals (0= ≤2 meals per day, 1=3 meals per day, 2= ≥4 meals per day), social and leisure activities (0= No, 1= Yes), experience of a traumatic event(0= No, 1= Yes), physical exercise habit(0= No, 1= Less than regular physical exercises, 2= Regular physical exercises), history of cardiovascular diseases (0= No, 1= Yes), history of liver disease (0= No, 1= Yes), antihyperlipidemic drugs (0= No, 1= Yes), and antidiabetic drugs (0= No, 1= Yes) were collected using a type of self-report method. Most variables were depending on our previous research studies[23-27].

Measurements

Triglycerides (TG) levels, low-density lipoprotein cholesterol (LDL-C), high-density lipoprotein cholesterol (HDL-C), and glycosylated hemoglobin (HbA1c) were analyzed by the enzymatic colorimetric tests, serum creatinine (Scr) and serum uric acid (SUA) levels were analyzed using the urinalysis (UA) plus method. The average value of blood pressure was measured by the mean of the 3-time measurements. BMI was calculated based on the measured weight and height of the participants. Tapeline was localized at navel levels to read the waist circle (WC) at the end of exhalation.

Statistical analysis

The data are presented as means and standard deviation (SD) unless indicated otherwise. Means and standard deviation (continuous data) were used to describe continuous variables (Age, age of menarche, LDL-C, HDL-C, TG, HbA1c, Scr, SUA, BMI, WC, DBP, and SBP levels), and number and percentage (categorical data) were used to assess the categorical variables (educational levels, marital status, residence places, alcohol drinking, smoking habit, eating meals, social and leisure

For peer review only - http://bmjopen.bmj.com/site/about/guidelines.xhtml

activities, the experience of a traumatic event, taking physical activity or exercise, history of CVDs, history of liver diseases, history of antilipidemic medication, anti-diabetic medication). Between-group differences according to the age of menarche (≤14years, 15years, 16years, 17years, ≥18years) were evaluated by the Student's t-test (continuous data) or chi-square test (categorical data). Differences between two categories of baseline characteristics (marital status, residence places, social and leisure activities, the experience of a traumatic event, taking physical activity or exercise, history of CVDs, history of antilipidemic medication, history of liver diseases, anti-diabetic medication) were also evaluated using t-test. However, the trend of related variables (LDL-C, HDL-C, TG, HbA1c, SUA, BMI, WC, DBP, and SBP levels) by above three categories of baseline characteristics (age of menarche levels, educational levels, alcohol drinking, smoking habit, eating meals, taking physical activity or exercise) was also tested using contrast analysis. Correlations between baseline characteristics and BP were used by Spearman's correlation test. However, relationships between continuous variables (Age, LDL-C, HDL-C, TG, HbA1c, Scr, SUA, BMI, WC and menarche age) and BP were used by Pearson's correlation test. The mediation analysis was performed by model 4 in process v3.3[28-30], and the moderation analysis was used model 1 to assessment. All covariates (including age, educational levels, marital status, place of residence, smoking habits, drinking habits, eating meals, social and leisure activities, the experience of a traumatic event, taking physical activity or exercise, history of cardiovascular diseases, hepatitis history, antihyperlipidemic drugs, antidiabetic drugs, LDL-C, HDL-C, TG, HbA1c, Scr, SUA,) were adjusted for in the mediation or moderation models. Analyses including the unstandardized (B), Standard Error (SE), or standardized coefficients (β) with 95% confidence intervals(95%CI) were calculated using the setting of modelling building in IBM SPSS22.0 software for Windows10 (IBM Corp., Armonk, NY, USA) using the bootstrap method, with a 5% significance level.

Patient and public involvement

No patients were involved in the development of the question, design or data interpretation.

.

For peer review only - http://bmjopen.bmj.com/site/about/guidelines.xhtml

RESULTS

The final sample consisted of 4513 women with an average age of 58.59 ± 9.31years (ranged from 45 to 96 years). The mean and standard deviation of menarche age were 16.27±2.18 years (ranged from 10 to 30 years). The mean and standard deviation of DBP and SBP level were 76.31±12.45mmHg (ranged from 39 to 136 mmHg), and 131.65±26.73 mmHg (ranged from 76 to 235 mmHg), respectively. The mean and standard deviation of BMI and WC level were 24.04±4.13 kg/m^2(ranged from 11.65 to 63.05 kg/m^2), and 84.67±12.83cm (ranged from 19.5 to 130.10 cm), respectively. Table 1 shows the baseline characteristics by the age of menarche. There were differences between the age of menarche in educational levels, place of residence, cigarette smoking, social events, and history of CVDs. However, between-group differences in the prevalence of marital status, drinking habits, eating habit, accidental injury history, physical exercises habit, hepatitis history, antilipidemic medication history, and antidiabetic medication history were not found. Women with a higher age of menarche had higher HCL-C level and age but had lower TG, SUA, BMI, WC, and DBP levels. Moreover, there were no differences in HbA1c, Scr, and SBP level between the age of menarche groups.

Table1 Baseline characteristics by age at menarche (N=4513)

Variables	≤14(years) N=946	15(years) N=671	16(years) N=930	17(years) N=702	≥18(years) N=1264	*P*
Age(years)	56.88±9.38	57.49±9.34	58.41±9.42	58.6±9.12	60.57±8.91	0.000[a]
Educational levels						
Illiterate	300(31.71）	233(34.72）	378(40.65）	314(44.73）	649(51.34）	0.000
Less than elementary school	527(55.71）	383(57.08）	486(52.26）	343(48.86）	572(45.25）	
High school	82(8.67）	42(6.26）	48(5.16）	34(4.84）	34(2.69）	
Above vocational school	37(3.91）	13(1.94）	18(1.94）	11(1.57）	9(0.71）	
Marital status						
Single	137(14.48）	79(11.77）	137(14.73）	98(13.96）	207(16.38）	0.101
Married	809(85.52）	592(88.23）	793(85.27）	604(86.04）	1057(83.62）	
Place of residence						
Rural	519(54.86）	426(63.49）	577(62.04）	454(64.67）	885(70.02）	0.000
Urban	427(45.14）	245(36.51）	353(37.96）	248(35.33）	379(29.98）	
Cigarette smoking						
NO	883(93.34）	629(93.74）	846(90.97）	646(92.02）	1156(91.46）	0.017
Former smoke	8(0.85）	11(1.64）	18(1.94）	13(1.85）	38(3.01）	
Current smoke	55(5.81）	31(4.62）	66(7.1）	43(6.13）	70(5.54）	
Alcohol habit						
NO	827(87.42）	607(90.46）	809(86.99）	620(88.32）	1100(87.03）	0.520
Less than once a month	45(4.76）	26(3.87）	48(5.16）	37(5.27）	65(5.14）	
More than once a month	74(7.82）	38(5.66）	73(7.85）	45(6.41）	99(7.83）	
Eating habit						
≤2 meals per day	131(13.85）	99(14.75）	123(13.23）	82(11.68）	172(13.61）	0.706
3 meals per day	804(84.99）	567(84.5）	794(85.38）	609(86.75）	1073(84.89）	
≥4 meals per day	11(1.16）	5(0.75）	13(1.40）	11(1.57）	19(1.50）	
Social events						
No	417(44.08）	335(49.93）	470(50.54）	372(52.99）	655(51.82）	0.002
Yes	529(55.92）	336(50.07）	460(49.46）	330(47.01）	609(48.18）	
History of accidental injury						
No	881(93.13）	630(93.89）	867(93.23）	652(92.88）	1169(92.48）	0.837
Yes	65(6.87）	41(6.11）	63(6.77）	50(7.12）	95(7.52）	
Physical exercises habit						
No physical exercise	587(62.05）	393(58.57）	556(59.78）	428(60.97）	771(61.00）	0.260
Less than regular physical exercises	178(18.82）	137(20.42）	171(18.39）	136(19.37）	271(21.44）	
Regular physical exercises	181(19.13）	141(21.01）	203(21.83）	138(19.66）	222(17.56）	
History of cardiovascular disease						
No	810(85.62）	590(87.93）	780(83.87）	605(86.18）	1115(88.21）	0.033
Yes	136(14.38）	81(12.07）	150(16.13）	97(13.82）	149(11.79）	

For peer review only - http://bmjopen.bmj.com/site/about/guidelines.xhtml

Hepatitis history						
No	910(96.19）	647(96.42）	885(95.16）	670(95.44）	1226(96.99）	0.205
Yes	36(3.81）	24(3.58）	45(4.84）	32(4.56）	38(3.01）	
History of antilipidemic medication						
No	884(93.45）	628(93.59）	884(95.05）	656(93.45）	1184(93.67）	0.566
Yes	62(6.55）	43(6.41）	46(4.95）	46(6.55）	80(6.33）	
History of antidiabetic medication						
No	910(96.19）	645(96.13）	883(94.95）	663(94.44）	1213(95.97）	0.309
Yes	36(3.81）	26(3.87）	47(5.05）	39(5.56）	51(4.03）	
LDL Cholesterol	119.66±35.04	118.04±35.61	120.62±34.81	121.79±36.79	121.45±35.27	0.065[a]
HDL Cholesterol	50.57±14.17	51.31±14.01	50.84±14.43	51.05±14.18	53.2±14.87	0.000[a]
Triglycerides	143.09±99.89	142.86±103.67	142.07±98.31	145.85±132.3	124.16±76.25	0.000[a]
HbA1c	5.31±0.83	5.25±0.86	5.31±0.89	5.32±0.84	5.27±0.76	0.594[a]
Scr	0.69±0.14	0.69±0.14	0.70±0.18	0.68±0.13	0.69±0.15	0.813[a]
SUA	4.09±1.10	3.99±1.09	4.04±1.07	4.01±1.01	3.97±1.05	0.021[a]
BMI	24.63±4.11	24.39±4.15	24.02±3.90	24.18±4.49	23.36±3.99	0.000[a]
WC	86.08±13.01	85.35±12.62	85.29±11.33	84.53±13.38	82.89±13.36	0.000[a]
DBP	77.07±12.07	77.28±12.53	77.01±12.51	76.31±12.80	74.72±25.82	0.000[a]
SBP	131.09±26.36	131.66±25.49	131.63±26.64	129.90±29.9	129.47±25.82	0.062[a]

[a]*P*-value for trend

Baseline characteristics, according to BMI and WC in women, are presented in Table 2. Firstly, women with more alcohol drinking, higher age of menarche had lower BMI and WC levels; Secondly, women with a history of cardiovascular diseases, antilipidemic medication, and antidiabetic medication had higher BMI and WC levels than women without medical illness. Thirdly, women with higher educational levels, being married, being current smoking, taking more eating meals, and taking social events had higher BML level. At last, women with accidental injury had higher WC level.

Table2 Baseline characteristics by age at menarche (N=4513)

Variables	BMI (cm)	WC (cm)	*P*-value for BMI	*P*-value for WC
Educational levels				
Illiterate	23.57±4.19	84.35±12.95	0.001[a]	0.971[a]
Less than elementary school	24.31±4.03	85.14±12.48		
High school	24.93±4.07	83.17±14.65		
Above vocational school	24.85±4.43	83.40±13.99		
Marital status				
Single	23.21±4.18	84.93±12.04	0.000	0.582
Married	24.19±4.10	84.63±12.97		
Place of residence				
Rural	23.64±3.96	83.95±12.42	0.000	0.000
Urban	24.75±4.32	85.94±13.43		
Cigarette smoking				
NO	24.11±4.12	84.73±12.77	0.000[a]	0.135[a]
Former smoke	23.79±4.14	86.83±10.36		
Current smoke	23.17±4.11	83.13±14.40		
Alcohol habit				
NO	24.10±4.14	85.02±12.5	0.011[a]	0.000[a]
Less than once a month	23.88±4.17	81.78±15.16		
More than once a month	23.51±3.94	82.49±14.62		
Eating habit				
≤2 meals per day	23.27±3.89	84.12±10.42	0.000[a]	0.734[a]
3 meals per day	24.18±4.14	84.82±13.18		
≥4 meals per day	23.10±4.69	80.82±12.01		
Social events				
No	23.64±4.09	84.34±12.08	0.000	0.085
Yes	24.45±4.13	85.00±13.53		
History of accidental injury				
No	24.06±4.14	84.82±12.57	0.474	0.004
Yes	23.88±3.94	82.64±15.81		
Physical exercises habit				
No physical exercise	24.05±4.29	84.79±12.74	0.400[a]	0.701[a]
Less than regular physical exercises	23.82±3.70	84.25±12.48		
Regular physical exercises	24.26±4.03	84.74±13.48		
History of cardiovascular disease				
No	23.92±4.09	84.26±12.63	0.000	0.000

For peer review only
For peer review only - http://bmjopen.bmj.com/site/about/guidelines.xhtml

Yes	24.83±4.28	87.25±13.89		
Hepatitis history				
No	24.04±4.15	84.66±12.79	0..871	0.948
Yes	24.09±3.47	84.73±14.37		
History of antilipidemic medication				
No	23.87±3.99	84.22±12.72	0.000	0.000
Yes	26.75±5.14	91.59±12.58		
History of antidiabetic medication				
No	23.95±4.01	84.42±12.81	0.000	0.000
Yes	26.13±5.81	90.18±12.20		
Menarche age(years)				
≤14	24.63±4.11	86.08±13.01	0.000 [a]	0.000 [a]
15	24.39±4.15	85.35±12.62		
16	24.02±3.90	85.29±11.33		
17	24.18±4.49	84.53±13.38		
≥18	23.36±13.36	82.89±13.36		

[a]*P*-value for trend

Baseline characteristics, according to DBP and SBP in women, are presented in Table 3. Firstly, women with more alcohol drinking had lower DBP and SBP levels; Secondly, women with a history of antilipidemic medication had higher DBP and SBP levels than women without medical illness. Thirdly, women with more eating meals, higher age of menarches had higher DBP level. At last, women with higher educational levels, being married, and more alcohol drinking had lower DBP level, but women with cardiovascular diseases and antidiabetic medication had higher SBP level than women without medical illness.

Table3 Baseline characteristics by age at menarche (N=4513)

Variables	DBP	SBP	*P*-value for DBP	*P*-value for SBP
Educational levels				
Illiterate	76.37±12.45	133.93±28.43	0.288[a]	0.000[a]
Less than elementary school	76.38±12.42	128.89±24.99		
High school	76.12±12.65	122.98±18.58		
Above vocational school	73.86±12.47	127.84±40.38		
Marital status				
Single	76.70±11.96	139.61±31.93	0.388	0.000
Married	76.25±12.53	129.12±25.42		
Place of residence				
Rural	76.20±12.72	130.18±25.92	0.435	0.121
Urban	76.50±11.96	131.46±28.06		
Cigarette smoking				
NO	76.32±12.45	130.43±26.97	0.673[a]	0.051[a]
Former smoke	73.60±11.41	132.13±22.83		
Current smoke	77.09±12.65	133.61±23.78		
Alcohol habit				
NO	76.53±12.38	131.27±27.27	0.003[a]	0.001[a]
Less than once a month	74.76±12.18	123.81±19.94		
More than once a month	74.73±13.26	127.77±23.06		
Eating habit				
≤2 meals per day	77.69±12.60	132.14±26.68	0.000 [a]	0.117 [a]
3 meals per day	76.17±12.41	130.44±26.37		
≥4 meals per day	71.56±11.46	128.63±44.41		
Social events				
No	76.08±12.60	130.66±26.33	0.216	0.975
Yes	76.54±12.30	130.64±27.12		
History of accidental injury				
No	76.38±12.47	130.84±27.12	0.219	0.075
Yes	75.48±12.14	128.05±20.66		
Physical exercises habit				
No physical exercise	76.34±12.46	131.47±28.25	0.873 [a]	0.024 [a]
Less than regular physical exercises	76.05±12.76	129.23±22.14		
Regular physical exercises	76.50±12.08	129.54±26.03		

For peer review only - http://bmjopen.bmj.com/site/about/guidelines.xhtml

History of cardiovascular disease				
No	76.11±12.44	130.02±26.29	0.021	0.000
Yes	77.39±12.40	134.50±29.28		
Hepatitis history				
No	76.36±12.45	130.76±26.50	0.017	0.144
Yes	74.10±11.56	127.76±32.79		
History of antilipidemic medication				
No	76.07±12.34	130.12±26.01	0.000	0.000
Yes	80.09±13.46	138.80±35.01		
History of antidiabetic medication				
No	76.25±12.47	130.17±25.46	0.100	0.000
Yes	77.73±11.83	140.96±45.17		
Menarche age(years)				
≤14				
15	77.07±12.07	131.09±26.36	0.000 [a]	0.062 [a]
16	77.28±12.53	131.66±25.49		
17	77.01±12.51	131.63±26.64		
≥18	76.31±12.80	129.90±29.90		

[a] *P*-value for trend

Correlations between baseline characteristics, independent, moderators, mediators and dependent variables, according to DBP and SBP, are presented in Table 4. Firstly, drinking, antilipidemic medication, LDL-C, HDL-C, TG, HbA_1c, Scr, SUA, BMI, and WC were significantly correlated with DBP and SBP in women; Secondly, eating meals, history of CVDs, history of liver diseases, and menarche age were significantly correlated with DBP; At last, age, educational levels, marital status, physical exercises, history of CVDs, and antidiabetic medication were significantly correlated with SBP.

Table4 Relationship between various characteristics and blood pressure status of participants (N=4513)

Variables	DBP r(*P*)	SBP r(*P*)
Age	-0.016(0.275)	0.259(0.000)
Education	-0.016(0.288)	-0.106(0.000)
Marital status	-0.013(0.388)	-0.139(0.000)
Current residence	0.012(0.435)	0.023(0.121)
Smoke	0.006(0.673)	0.029(0.051)
Drinking	-0.045(0.003)	-0.051(0.001)
Eating meals	-0.055(0.000)	-0.023(0.117)
Taking activities	0.018(0.216)	0.000(0.975)
Major accidental injury	-0.018(0.219)	-0.027(0.075)
Having regular physical exercises	0.002(0.873)	-0.034(0.024)
History of CVD	0.035(0.018)	0.058(0.000)
History of liver diseases	-0.035(0.018)	-0.022(0.146)
Antilipidemic medication	0.078(0.000)	0.078(0.000)
Antidiabetic medication	0.025(0.100)	0.083(0.000)
LDL Cholesterol	0.050(0.001)	0.060(0.000)
HDL Cholesterol	-0.099(0.000)	-0.062(0.000)
Triglycerides	0.100(0.000)	0.091(0.000)
HbA_1c	0.061(0.000)	0.066(0.000)
Scr	0.056(0.000)	0.076(0.000)

For peer review only - http://bmjopen.bmj.com/site/about/guidelines.xhtml

SUA	0.085(0.000)	0.111(0.000)
BMI	0.221(0.000)	0.129(0.000)
WC	0.183(0.000)	0.177(0.000)
Menarche age	-0.060(0.000)	-0.014(0.335)

In the moderator analysis, we tested the interaction between menarche age and obesity in predicting systolic and diastolic BP. The standard error, standardized coefficients and the adjusting associated 95% confidence intervals (CIs) are shown in Table 5. We controlled for socio-demographic characteristics, health behaviors, medical histories, and metabolic measures. After controlling for the total potential confounders, the moderating effect of obesity indicators on the relationship between age of menarche and BP was tested. The interaction term of obesity parameters*age of menarche was not significant for predicting either DBP (BMI, adjusted P= 0.2556; WC, adjusted P= 0.1833) or SBP (BMI, adjusted P= 0.8561; WC, adjusted P= 0.8427) in women.

For peer review only

Table5 Moderator effect of obesity on the relationship between menarche age and BP in women (N=4513)

Dependent	Variables	Model one[a]			Model two[b]			Model three[c]			Model four[d]			Model five[e]		
		SE	B (95%*CI*)	*P*	se	B (95%*CI*)	*P*	SE	B (95%*CI*)	*P*	SE	B (95%*CI*)	*P*	se	B (95%*CI*)	*P*
DBP	Menarche age	0.0835	-0.2027(-0.3665, -0.0390)	0.0153	0.0855	-0.2528(-0.4203, -0.0852)	0.0031	0.0853	-0.2411(-0.4084, -0.0738)	0.0047	0.0854	-0.2485(-0.4158, -0.0811)	0.0036	0.0973	-0.2162(-0.4069, -0.0254)	0.0263
	BMI	0.0440	0.6546(0.5684,0.7409)	0.0000	0.0447	0.6757(0.5881,0.7634)	0.0000	0.0449	0.6835(0.5955,0.7715)	0.0000	0.0458	0.6647(0.5750,0.7544)	0.0000	0.0556	0.5916(0.4827,0.7005)	0.0000
	BMI× Menarche age	0.0204	0.0279(-0.0121,0.0678)	0.1719	0.0204	0.0258(-0.0141,0.0658)	0.2046	0.0203	0.0247(-0.0152,0.0645)	0.2248	0.0203	0.0220(-0.0178,0.0618)	0.2790	0.0229	0.0260(-0.0189,0.071)	0.2556
SBP	Menarche age	0.1826	-0.0003(-0.3583,0.3576)	0.9986	0.1789	-0.5822(-0.9329, -0.2315)	0.0011	0.1788	-0.5682(-0.9187, -0.2177)	0.0015	0.1795	-0.5944(-0.9462, -0.2425)	0.0009	0.2141	-0.5084(-0.9282, -0.0886)	0.0176
	BMI	0.0964	0.8361(0.6470,1.0251)	0.0000	0.0938	1.0927(0.9088,1.2766)	0.0000	0.0942	1.0906(0.9058,1.2753)	0.0000	0.0965	1.0287(0.8396,1.2178)	0.0000	0.1223	0.8846(0.6448,1.1244)	0.0000
	BMI× Menarche age	0.0446	0.0178(-0.0696,0.1052)	0.6896	0.0426	0.0111(-0.0726,0.0947)	0.7955	0.0426	0.0113(-0.0722,0.0948)	0.7914	0.0427	0.0052(-0.0786,0.0889)	0.9038	0.0504	0.0091(-0.0897,0.108)	0.8561
DBP	Menarche age	0.0838	-0.2569(-0.4213, -0.0925)	0.0022	0.0861	-0.2588(-0.4277, -0.0900)	0.0027	0.0861	-0.2481(-0.4168, -0.0794)	0.0040	0.0861	-0.2561(-0.4248, -0.0873)	0.0029	0.0979	-0.2001(-0.3921, -0.0082)	0.0410
	WC	0.0142	0.1732(0.1452,0.2011)	0.0000	0.0143	0.1752(0.1471,0.2032)	0.0000	0.0143	0.1730(0.1449,0.2011)	0.0000	0.0145	0.1645(0.1361,0.193)	0.0000	0.0172	0.1472(0.1135,0.1809)	0.0000
	WC× Menarche age	0.0065	0.0058(-0.007,0.0186)	0.3758	0.0066	0.0054(-0.0075,0.0182)	0.4136	0.0065	0.005(-0.0079,0.0178)	0.4473	0.0065	0.0043(-0.0085,0.0171)	0.5075	0.0074	0.0099(-0.0047,0.0244)	0.1833
SBP	Menarche age	0.1777	-0.0009(-0.3492,0.3475)	0.9961	0.1761	-0.5656(-0.9107, -0.2204)	0.0013	0.1760	-0.5546(-0.8997, -0.2094)	0.0016	0.1766	-0.581(-0.9273, -0.2347)	0.0010	0.2096	-0.4548(-0.8658, -0.0438)	0.0301
	WC	0.0302	0.3637(0.3044,0.4229)	0.0000	0.0293	0.3228(0.2654,0.3802)	0.0000	0.0294	0.3153(0.2578,0.3729)	0.0000	0.0298	0.2944(0.236,0.3528)	0.0000	0.0368	0.2744(0.2022,0.3466)	0.0000
	WC× Menarche age	0.0139	-0.0156(-0.0428,0.0116)	0.2607	0.0134	-0.0061(-0.0323,0.0202)	0.6501	0.0134	-0.0063(-0.0326,0.0199)	0.6367	0.0134	-0.0081(-0.0344,0.0182)	0.5443	0.0159	-0.0032(-0.0343,0.028)	0.8427

BMI: body mass index; WC: waist circle; DBP: diastolic blood pressure; SUA: serum uric acid; SBP: systolic blood pressure; Un-standardized: B; Standard Error: SE.

[a] **Unadjusted;**

[b] **Adjusted for** age, educational levels, marital status, place of residence;

[c] **Adjusted for** age, educational levels, marital status, place of residence, smoking habits, drinking habits, eating meals, social and leisure activities, experience of a traumatic event, taking physical activity or exercise;

[d] **Adjusted for** age, educational levels, marital status, place of residence, smoking habits, drinking habits, eating meals, social and leisure activities, experience of a traumatic event, taking physical activity or exercise, history of cardiovascular disease, hepatitis history, antihyperlipidemic drugs, antidiabetic drugs;

[e] **Adjusted for** age, educational levels, marital status, place of residence, smoking habits, drinking habits, eating meals, social and leisure activities, experience of a traumatic event, taking physical activity or exercise, history of cardiovascular disease, hepatitis history, antihyperlipidemic drugs, , antidiabetic drugs, LDL Cholesterol, HDL Cholesterol, Triglycerides, HbA1c, serum creatinine, serum uric acid.

For peer review only

For peer review only - http://bmjopen.bmj.com/site/about/guidelines.xhtml

In the mediation analysis, we estimated the BP equation using bootstrap inference for model coefficients. The un-standardized, standardized coefficients and the adjusting associated 95% confidence intervals (CIs) are shown in Table 6. We controlled for socio-demographic characteristics, health behaviors, medical histories, and metabolic measures. After controlling for the total potential confounders, the mediating effect of obesity indicators on the relationship between age of menarche and BP was tested. All correlations were significant correlation between age of menarche, obesity parameters and BP except the path of the age of menarche→BP (with the addition of the BMI or WC indicator) in model one. In general, after controlling for potential confounders. WC and BMI partly mediated the relationship between age of menarche and BP.

For peer review only

For peer review only - http://bmjopen.bmj.com/site/about/guidelines.xhtml

Table6 Mediation effect of obesity on the relationship between age at menarche and BP in women (N=4513)

Path		Model one[a]			Model two[b]			Model three[c]			Model four[d]			Model five[e]		
		β	B (95%*CI*)	*P*	β	B (95%*CI*)	*P*	β	B (95%*CI*)	*P*	β	B (95%*CI*)	*P*	β	B (95%*CI*)	*P*
**Path1**	Menarche age→BMI	-0.1080	-0.2045(-0.2595, -0.1496)	0.0000	-0.0725	-0.1374(-0.1930, -0.0818)	0.0000	-0.0707	-0.1339(-0.1892, -0.0786)	0.0000	-0.0705	-0.1334(-0.1880, -0.0788)	0.0000	-0.0468	-0.0885(-0.1409, -0.0361)	0.0009
	Menarche age→DBP	-0.0367	-0.2096(-0.3731, -0.0461)	0.0120	-0.0454	-0.2596(-0.4268, -0.0924)	0.0024	-0.0433	-0.2475(-0.4145, -0.0806)	0.0037	-0.0447	-0.2542(-0.4213, -0.0872)	0.0029	-0.0379	-0.2154(-0.3833, -0.0474)	0.0120
	BMI→DBP	0.2171	0.6545(0.5682,0.7408)	0.0000	0.2241	0.6757(0.5881, 0.7634)	0.0000	0.2276	0.6835(0.5955, 0.7715)	0.0000	0.2208	0.6643(0.5746,0.7541)	0.0000	0.1986	0.5973(0.5026,0.6921)	0.0000
	Total effect	-0.0601	-0.3435(-0.5099, -0.177)	0.0001	-0.0617	-0.3524(-0.5234, -0.1815)	0.0001	-0.0593	-0.3390(-0.5098, -0.1683)	0.0001	-0.0602	-0.3429(-0.5133, -0.1724)	0.0001	-0.0471	-0.2682(-0.4388, -0.0976)	0.0021
**Path2**	Menarche age→BMI	0.1085	-0.2051(-0.2601, -0.1502)	0.0000	-0.0731	-0.1381(-0.1936, -0.0826)	0.0000	-0.0712	-0.1346(-0.1898, -0.0793)	0.0000	-0.0711	-0.1342(-0.1887, -0.0797)	0.0000	-0.0473	-0.0892(-0.1416, -0.0369)	0.0008
	Menarche age→SBP	-0.0004	-0.0046(-0.3619,0.3526)	0.9797	-0.0477	-0.585(-0.9350, -0.2351)	0.0011	-0.0466	-0.5711(-0.9209, -0.2213)	0.0014	-0.0486	-0.5957(-0.9468, -0.2446)	0.0009	-0.0446	-0.5479(-0.9025, -0.1932)	0.0025
	BMI→SBP	0.1289	0.8359(0.6469,1.0249)	0.0000	0.1685	1.0927(0.9088, 1.2766)	0.0000	0.1682	1.0905(0.9058,1.2753)	0.0000	0.1585	1.0286(0.8395,1.2176)	0.0000	0.1441	0.9366(0.7361,1.1371)	0.0000
	Total effect	-0.0144	-0.1761(-0.5342,0.182)	0.3350	-0.0601	-0.736(-1.0902, -0.3817)	0.0000	-0.0586	-0.7178(-1.0719, -0.3638)	0.0001	-0.0599	-0.7337(-1.0883, -0.3791)	0.0001	-0.0515	-0.6314(-0.9889, -0.2739)	0.0005
**Path3**	Menarche age→WC	-0.0837	-0.4928(-0.6642, -0.3213)	0.0000	-0.0910	-0.5355(-0.7107, -0.3602)	0.0000	-0.0896	-0.5275(-0.7024, -0.3526)	0.0000	-0.0898	-0.5285(-0.7026, -0.3545)	0.0000	-0.0696	-0.4085(-0.5778, -0.2392)	0.0000
	Menarche age→DBP	-0.0454	-0.2589(-0.4232, -0.0945)	0.0020	-0.0456	-0.26(-0.4288, -0.0912)	0.0025	-0.0437	-0.2491(-0.4178, -0.0805)	0.0038	-0.0452	-0.2571(-0.4257, -0.0884)	0.0028	-0.0373	-0.2118(-0.3811, -0.0424)	0.0143
	WC→DBP	0.1789	0.1733(0.1454, 0.2013)	0.0000	-0.1810	0.1754(0.1473, 0.2034)	0.0000	0.1787	0.1732(0.1451, 0.2013)	0.0000	0.1705	0.1647(0.1363, 0.1931)	0.0000	0.1445	0.1399(0.1104, 0.1695)	0.0000
	Total effect	-0.0604	-0.3443(-0.5107, -0.1779)	0.0001	-0.0620	-0.3539(-0.5248, -0.183)	0.0001	-0.0597	-0.3405(-0.5112, -0.1698)	0.0001	-0.0606	-0.3441(-0.5145, -0.1737)	0.0001	-0.0474	-0.2689(-0.4395, -0.0984)	0.0020
**Path4**	Menarche age→WC	-0.0849	-0.4944(-0.6658, -0.323)	0.0000	-0.0914	-0.5376(-0.7129, -0.3624)	0.0000	-0.0901	-0.5299(-0.7048, -0.355)	0.0000	-0.0904	-0.531(-0.7051, -0.357)	0.0000	-0.0702	-0.411(-0.5803, -0.2417)	0.0000
	Menarche age→SBP	0.0004	0.0044(-0.3439, 0.3526)	0.9804	-0.0468	-0.5643(-0.9094, -0.2192)	0.0014	-0.0459	-0.5533(-0.8983, -0.2082)	0.0017	-0.0481	-0.5792(-0.9255, -0.233)	0.0010	-0.0433	-0.5228(-0.8721, -0.1735)	0.0034
	WC→SBP	0.1772	0.3632(0.3040, 0.4224)	0.0000	0.1574	0.3226(0.2652,0.38)	0.0000	0.1538	0.3151(0.2576, 0.3727)	0.0000	0.1436	0.2942(0.2358, 0.3526)	0.0000	0.1290	0.2655(0.2045,0.3266)	0.0000
	Total effect	-0.0145	-0.1752(-0.5277, 0.1773)	0.3300	-0.0612	-0.7377(-1.086, -0.3894)	0.0000	-0.0598	-0.7202(-1.0683, -0.3722)	0.0001	-0.0611	-0.7354(-1.084, -0.3869)	0.0000	-0.0524	-0.632(-0.9832, -0.2807)	0.0004

Path1: Mediation effect of BMI on the relationship between age at menarche and DBP.
Path2: Mediation effect of BMI on the relationship between age at menarche and SBP.
Path3: Mediation effect of WC on the relationship between age at menarche and DBP.
Path4: Mediation effect of WC on the relationship between age at menarche and SBP.
Un-standardized: B, standardized coefficients, β.
[a] **Unadjusted;**
[b] **Adjusted for** age, educational levels, marital status, place of residence;
[c] **Adjusted for** age, educational levels, marital status, place of residence, cigarette smoking, alcohol habit, eating habit, social events, history of accidental injury, physical exercises habit;
[d] **Adjusted for** age, educational levels, marital status, place of residence, cigarette smoking, alcohol habit, eating habit, social events, history of accidental injury, physical exercises habit, history of cardiovascular disease, hepatitis history, history of antilipidemic medication, history of antidiabetic medication;
[e] **Adjusted for** age, educational levels, marital status, place of residence, cigarette smoking, alcohol habit, eating habit, social events, history of accidental injury, physical exercises habit, history of cardiovascular disease, hepatitis history, history of antilipidemic medication, , history of antidiabetic medication, LDL Cholesterol, HDL Cholesterol, Triglycerides, HbA1c, serum creatinine, serum uric acid.

For peer review only - http://bmjopen.bmj.com/site/about/guidelines.xhtml

For peer review only

DISCUSSION

In the study, we attempted to explore the association between age of menarche and blood pressure and examined the moderating and mediating effect of obesity parameters on the association. We have found that the interaction term of obesity parameters*age of menarche was not significant for predicting BP. It was also important to note that WC and BMI partly mediated the relationship between age of menarche and BP. The results of our study were partly constant with most previous studies, even better than the previous studies. And so previous studier had only focus on the relationship between earlier biological maturation and health outcomes in late adolescence and adulthood. Most of the previous studies meant exactly that: earlier biological maturation was at a higher risk for health outcomes in late adolescence and adulthood. Furthermore, most previous studies found that early biological maturation (age of menarche) or puberty was significantly related to obesity parameters[31-35], hypertension[35-38], and metabolic syndrome[39-47]. In general, our results were mostly consistent with the results of previous studies that suggest that earlier biological maturation is a risk factor for several negative health outcomes in late adolescence and adulthood.

Our study suggested the likelihood of a dose-response relationship between age of menarche and obesity parameters (BMI and WC). The relationship could be identified by the fact that early-maturing girls already have more adiposity before menarche, given that fatty tissue, through leptin release, has a crucial role in the initiation of the biological maturation process[48 49]. Early maturing girls could maintain their higher fatty tissue in adulthood[50]. Another mechanism may also be associated with the time of the maturation process. It has been reported that early maturing girls could experience a more accelerated maturation process than late maturing ones. Thus, more significant interference with biological maturation in girls can lead to increased more fatty tissue[51]. We also found a significantly negative relationship between age at menarche and BP in mid-aged and elderly Chinese women. However, this association did not exist between the age of menarche and SBP before adjusting the related confounders. This result consistent with previous findings[52-60] regarding the age of menarche as a cardiovascular risk factor. The weak association between age at menarche and BP in adulthood could, in part, be explained by the fact that early matures were younger than women with late menarche. This result could be an expression of the fact that BP is an outcome with a higher latency period than obesity. Moreover, among women who already entered menopause in our study, the effects of menarche could be even weaker, as results suggest, and obesity could have more effect on BP. Therefore, obesity in childhood/adolescence,

For peer review only - http://bmjopen.bmj.com/site/about/guidelines.xhtml

sociodemographic, or even behavioral factors through life (including age, educational levels, marital status, place of residence, smoking habits, drinking habits, eating meals, social and leisure activities, the experience of a traumatic event, taking physical activity or exercise) were potential mediators to target when interventions for BP control and management.

Though so many studies have explored the association analysis between the age of menarche or obesity parameters and BP, there were only two studies[61 62] explored the mediating effect of obesity parameters on the relation between age of menarche and BP. Zhang L, Li Y, Zhou W, et al [61] found that The relationship between age of menarche and DBP was partly mediated by the obesity parameter. In contrast, the relationship was fully mediated by the obesity parameter in women. Werneck AO, Oyeyemi AL, Cyrino ES, et al [62] showed that women with late menarche are less likely to have a risk of hypertension, and BMI was an important mediator of the age at the menarche-hypertension association. The interactions term of obesity parameters*age of menarche in the two studies were the same as our study. Interestingly, we found that obesity parameters partly mediated the relationship between age of menarche and BP in women. The difference between our research and others may due to the different population, different definitions of early and later menarche, and different confounding variables by controlling. The individuals in our study were mid-aged and elderly Chinese women, where the mean age at recruitment and age of menarche were older than in the Werneck AO, Oyeyemi AL, Cyrino ES, et al’ s study, and the level of socio-economic development also made some contribution to ontogenetic development. In addition, other studies and a meta-analysis[52 54 60 63-65] reported an inverse association between early age of menarche and hypertension, which was not observed in Zhang L, Li Y, Zhou W, et al’ study. They found no direct relationship between the age of menarche and hypertension, and we have found a direct relationship between age of menarche and BP. The participants were similar in China and its socio-economic background, this phenomenon could be explained by the cumulative effect, which showed a significantly negative association between age of menarche and BP over life-span.

In sum, the interaction term of obesity parameters*age of menarche was not significant for predicting either DBP or SBP in women. Moreover, obesity parameters partly mediated the relationship between age of menarche and BP.

For peer review only - http://bmjopen.bmj.com/site/about/guidelines.xhtml

CONCLUSIONS

The interaction term of obesity parameters*age of menarche was not significant for predicting either DBP or SBP in women. Moreover, Obesity parameters partly mediated the relationship between age of menarche and BP.

Author affiliations

[1] Department of Internal Medicine Nursing, School of Nursing, Wannan Medical College, 22 Wenchang West Road, Higher Education Park, Wuhu City, An Hui Province, P.R.China

[2] Obstetrics and Gynecology Nursing, School of Nursing, Wannan Medical College, 22 Wenchang West Road, Higher Education Park, Wuhu City, An Hui Province, P.R.China

[3]Department of Pediatric Nursing, School of Nursing, Wannan Medical College, 22 Wenchang West Road, Higher Education Park, Wuhu City, An Hui Province, P.R.China

[4] Department of Surgical Nursing, School of Nursing, Wannan Medical College, 22 Wenchang West Road, Higher Education Park, Wuhu City, An Hui Province, P.R.China

[5]Department of Emergency and Critical Care Nursing, School of Nursing, Wannan Medical College, 22 Wenchang West Road, Higher Education Park, Wuhu City, An Hui Province, P.R.China

[6]Rehabilitation Nursing, School of Nursing, Wanna Medical college, 22 Wenchang West Road, Higher Education Park, Wuhu City, An Hui Province, P.R.China

[7]Department of nursing, Yijishan Hospital, the First Affiliated Hospital of Wannan Medical College, Zheshan West Road, Yijishan District, Wuhu City, Anhui Province

For peer review only

For peer review only - http://bmjopen.bmj.com/site/about/guidelines.xhtml

Abbreviations

CHARLS, China Health and Retirement Longitudinal Study; World Health Organization, WHO; BMI, body mass index; waist circle, WC; BP, blood pressure; DBP, diastolic blood pressure; SBP, systolic blood pressure; SUA, serum uric acid; CVDs, cardiovascular diseases; M, mean; standard error, SE; unstandardized, B; standardized coefficients, β; CDC, Centers for Disease Control and Prevention; Scr, serum creatinine; SD, standard deviation; LDL, low-density lipoprotein; Hemoglobin A1C, HbA1c; confidence intervals, CIs; HDL, high-density lipoprotein; estimated glomerular filtration rate, eGFR; fasting plasma glucose, FPG; NSFC, The National Natural Science Foundation of China; National Institute on Aging, NIA; World Bank, WB; urinalysis, UA.

Acknowledgements

We thank the members of the research as well as all participants for their contribution.

Author contributions

Conceived and designed the research: LZ. Wrote the paper: LZ. Analyzed the data: LZ. Revised the paper: LZ, L Y, C-z W, TY, Dm Z, H-h W, JL, Y-x L, LS, X-p L, YH, H-y C, and Y-z L.

Funding

CHARLS was supported by the NSFC (70910107022, 71130002) and National Institute on Aging (R03-TW008358-01; R01-AG037031-03S1), and World Bank (7159234) and the Support Program for Outstanding Young Talents from the Universities and Colleges of Anhui Province for Lin Zhang.

Disclosure

All authors report no potential conflict of interest relevant to the research.

Patient consent

Completion of all authors declaration and consent to publish form is required.

Ethics approval and consent to participate

All data are openly published as microdata at http://charls.pku.edu.cn/index/zh-cn.html with no direct contact with all participants.

For peer review only - http://bmjopen.bmj.com/site/about/guidelines.xhtml

For peer review only

Data sharing

Data sharing statement Extra data can be accessed viahttp://charls.pku.edu.cn/index/zh-cn.html.

References

1. Tian S, Xu Y, Dong H. The effect of metabolic health and obesity phenotypes on risk of hypertension: A nationwide population-based study using 5 representative definitions of metabolic health. Medicine (Baltimore) 2018;**97**(38):e12425 doi: 10.1097/MD.0000000000012425[published Online First: Epub Date]|.
2. Abdulsalam S, Olugbenga-Bello A, Olarewaju O, et al. Sociodemographic correlates of modifiable risk factors for hypertension in a rural local government area of oyo state South west Nigeria. Int J Hypertens 2014;**2014**:842028 doi: 10.1155/2014/842028[published Online First: Epub Date]|.
3. Ke L, Ho J, Feng J, et al. Modifiable risk factors including sunlight exposure and fish consumption are associated with risk of hypertension in a large representative population from Macau. J Steroid Biochem Mol Biol 2014;**144 Pt A**:152-5 doi: 10.1016/j.jsbmb.2013.10.019[published Online First: Epub Date]|.
4. Ibekwe R. Modifiable Risk factors of Hypertension and Socio-demographic Profile in Oghara, Delta State; Prevalence and Correlates. Ann Med Health Sci Res 2015;**5**(1):71-7 doi: 10.4103/2141-9248.149793[published Online First: Epub Date]|.
5. Pilakkadavath Z, Shaffi M. Modifiable risk factors of hypertension: A hospital-based case-control study from Kerala, India. J Family Med Prim Care 2016;**5**(1):114-9 doi: 10.4103/2249-4863.184634[published Online First: Epub Date]|.
6. Mamudu HM, Paul TK, Wang L, et al. Association Between Multiple Modifiable Risk Factors of Cardiovascular Disease and Hypertension among Asymptomatic Patients in Central Appalachia. South Med J 2017;**110**(2):90-96 doi: 10.14423/SMJ.0000000000000602[published Online First: Epub Date]|.
7. Collaboration NCDRF. Worldwide trends in blood pressure from 1975 to 2015: a pooled analysis of 1479 population-based measurement studies with 19.1 million participants. Lancet 2017;**389**(10064):37-55 doi: 10.1016/S0140-6736(16)31919-5[published Online First: Epub Date]|.
8. Bromfield S, Muntner P. High blood pressure: the leading global burden of disease risk factor and the need for worldwide prevention programs. Curr Hypertens Rep 2013;**15**(3):134-6 doi: 10.1007/s11906-013-0340-9[published Online First: Epub Date]|.
9. Mahajan S, Zhang D, He S, et al. Prevalence, Awareness, and Treatment of Isolated Diastolic Hypertension: Insights From the China PEACE Million Persons Project. J Am Heart Assoc 2019;**8**(19):e012954 doi: 10.1161/JAHA.119.012954[published Online First: Epub Date]|.
10. Cloutier L, Morris D, Bruneau J, et al. World Health Organization celebrates World Health Day, April 7,2013--focusing on hypertension. Canadian journal of cardiovascular nursing = Journal canadien en soins infirmiers cardio-vasculaires 2013;**23**(2):9-11
11. Webb MS. Stress management strategies in hypertension control. Ethnicity & disease 2002;**12**(4):S3-95-100
12. Kotchen TA. The search for strategies to control hypertension. Circulation 2010;**122**(12):1141-3 doi: 10.1161/CIRCULATIONAHA.110.978759[published Online First: Epub Date]|.
13. Mancia G, Rea F, Cuspidi C, et al. Blood pressure control in hypertension. Pros and cons of available treatment strategies. Journal of hypertension 2017;**35**(2):225-33 doi: 10.1097/HJH.0000000000001181[published Online First: Epub Date]|.
14. Werneck AO, Silva DR, Souza MF, et al. Correlates of Blood Pressure According to Early, On Time, and Late Maturation in Adolescents. Journal of clinical hypertension 2016;**18**(5):424-30 doi: 10.1111/jch.12699[published Online First: Epub Date]|.
15. Widen E, Silventoinen K, Sovio U, et al. Pubertal timing and growth influences cardiometabolic risk factors in adult

For peer review only - http://bmjopen.bmj.com/site/about/guidelines.xhtml

males and females. Diabetes care 2012;**35**(4):850-6 doi: 10.2337/dc11-1365[published Online First: Epub Date]|.

16. Mueller NT, Duncan BB, Barreto SM, et al. Earlier age at menarche is associated with higher diabetes risk and cardiometabolic disease risk factors in Brazilian adults: Brazilian Longitudinal Study of Adult Health (ELSA-Brasil). Cardiovasc Diabetol 2014;**13**:22 doi: 10.1186/1475-2840-13-22[published Online First: Epub Date]|.
17. Dreyfus J, Jacobs DR, Jr., Mueller N, et al. Age at Menarche and Cardiometabolic Risk in Adulthood: The Coronary Artery Risk Development in Young Adults Study. J Pediatr 2015;**167**(2):344-52 e1 doi: 10.1016/j.jpeds.2015.04.032[published Online First: Epub Date]|.
18. Cao M, Cui B. Negative Effects of Age at Menarche on Risk of Cardiometabolic Diseases in Adulthood: A Mendelian Randomization Study. The Journal of clinical endocrinology and metabolism 2020;**105**(2) doi: 10.1210/clinem/dgz071[published Online First: Epub Date]|.
19. Power C, Lake JK, Cole TJ. Body mass index and height from childhood to adulthood in the 1958 British born cohort. The American journal of clinical nutrition 1997;**66**(5):1094-101 doi: 10.1093/ajcn/66.5.1094[published Online First: Epub Date]|.
20. Adair LS, Gordon-Larsen P. Maturational timing and overweight prevalence in US adolescent girls. American journal of public health 2001;**91**(4):642-4 doi: 10.2105/ajph.91.4.642[published Online First: Epub Date]|.
21. Must A, Naumova EN, Phillips SM, et al. Childhood overweight and maturational timing in the development of adult overweight and fatness: the Newton Girls Study and its follow-up. Pediatrics 2005;**116**(3):620-7 doi: 10.1542/peds.2004-1604[published Online First: Epub Date]|.
22. Li C, Liu T, Sun W, et al. Prevalence and risk factors of arthritis in a middle-aged and older Chinese population: the China health and retirement longitudinal study. Rheumatology 2015;**54**(4):697-706 doi: 10.1093/rheumatology/keu391[published Online First: Epub Date]|.
23. Zhang L, Liu K, Li H, et al. Relationship between body mass index and depressive symptoms: the "fat and jolly" hypothesis for the middle-aged and elderly in China. BMC public health 2016;**16**(1):1201 doi: 10.1186/s12889-016-3864-5[published Online First: Epub Date]|.
24. Zhang L, Li JL, Zhang LL, et al. Association and Interaction Analysis of Body Mass Index and Triglycerides Level with Blood Pressure in Elderly Individuals in China. BioMed research international 2018;**2018**:8934534 doi: 10.1155/2018/8934534[published Online First: Epub Date]|.
25. Zhang L, Li JL, Zhang LL, et al. No association between C-reactive protein and depressive symptoms among the middle-aged and elderly in China Evidence from the China Health and Retirement Longitudinal Study. Medicine 2018;**97**(38) doi: ARTN e12352

10.1097/MD.0000000000012352[published Online First: Epub Date]|.

26. Zhang L, Li JL, Zhang LL, et al. Relationship between adiposity parameters and cognition: the "fat and jolly" hypothesis in middle-aged and elderly people in China. Medicine 2019;**98**(10) doi: ARTN e14747

10.1097/MD.0000000000014747[published Online First: Epub Date]|.

27. Zhang L, Li JL, Zhang LL, et al. Body mass index and serum uric acid level: Individual and combined effects on blood pressure in middle-aged and older individuals in China. Medicine (Baltimore) 2020;**99**(9):e19418 doi: 10.1097/MD.0000000000019418[published Online First: Epub Date]|.
28. Hayes AF, Preacher KJ. Quantifying and Testing Indirect Effects in Simple Mediation Models When the Constituent Paths Are Nonlinear. Multivariate Behav Res 2010;**45**(4):627-60 doi: 10.1080/00273171.2010.498290[published Online First: Epub Date]|.
29. Hayes AF, Preacher KJ. Statistical mediation analysis with a multicategorical independent variable. Br J Math Stat Psychol 2014;**67**(3):451-70 doi: 10.1111/bmsp.12028[published Online First: Epub Date]|.
30. Hayes AF. An Index and Test of Linear Moderated Mediation. Multivariate Behav Res 2015;**50**(1):1-22 doi: 10.1080/00273171.2014.962683[published Online First: Epub Date]|.
31. Shrestha A, Olsen J, Ramlau-Hansen CH, et al. Obesity and age at menarche. Fertil Steril 2011;**95**(8):2732-4 doi:

For peer review only

For peer review only - http://bmjopen.bmj.com/site/about/guidelines.xhtml

10.1016/j.fertnstert.2011.02.020[published Online First: Epub Date]|.

32. Bralic I, Tahirovic H, Matanic D, et al. Association of early menarche age and overweight/obesity. J Pediatr Endocrinol Metab 2012;**25**(1-2):57-62 doi: 10.1515/jpem-2011-0277[published Online First: Epub Date]|.
33. Leitao RB, Rodrigues LP, Neves L, et al. Development of adiposity, obesity and age at menarche: an 8-year follow-up study in Portuguese schoolgirls. International journal of adolescent medicine and health 2013;**25**(1):55-63 doi: 10.1515/ijamh-2013-0007[published Online First: Epub Date]|.
34. Gallicchio L, Flaws JA, Smith RL. Age at menarche, androgen concentrations, and midlife obesity: findings from the Midlife Women's Health Study. Menopause 2016;**23**(11):1182-88 doi: 10.1097/GME.0000000000000691[published Online First: Epub Date]|.
35. Liu G, Yang Y, Huang W, et al. Association of age at menarche with obesity and hypertension among southwestern Chinese women: a new finding. Menopause 2018;**25**(5):546-53 doi: 10.1097/GME.0000000000001027[published Online First: Epub Date]|.
36. Zhou W, Wang T, Zhu L, et al. Association between Age at Menarche and Hypertension among Females in Southern China: A Cross-Sectional Study. Int J Hypertens 2019;**2019**:9473182 doi: 10.1155/2019/9473182[published Online First: Epub Date]|.
37. Shen L, Wang L, Hu Y, et al. Associations of the ages at menarche and menopause with blood pressure and hypertension among middle-aged and older Chinese women: a cross-sectional analysis of the baseline data of the China Health and Retirement Longitudinal Study. Hypertension research : official journal of the Japanese Society of Hypertension 2019;**42**(5):730-38 doi: 10.1038/s41440-019-0235-5[published Online First: Epub Date]|.
38. Guo L, Peng C, Xu H, et al. Age at menarche and prevention of hypertension through lifestyle in young Chinese adult women: result from project ELEFANT. BMC Womens Health 2018;**18**(1):182 doi: 10.1186/s12905-018-0677-y[published Online First: Epub Date]|.
39. Stockl D, Meisinger C, Peters A, et al. Age at menarche and its association with the metabolic syndrome and its components: results from the KORA F4 study. PloS one 2011;**6**(10):e26076 doi: 10.1371/journal.pone.0026076[published Online First: Epub Date]|.
40. Chang CJ, Lai MM, Lin CC, et al. Age at menarche and its association with the metabolic syndrome in Taiwan. Obesity research & clinical practice 2016;**10 Suppl 1**:S26-S34 doi: 10.1016/j.orcp.2015.10.003[published Online First: Epub Date]|.
41. Heys M, Schooling CM, Jiang C, et al. Age of menarche and the metabolic syndrome in China. Epidemiology 2007;**18**(6):740-6 doi: 10.1097/EDE.0b013e3181567faf[published Online First: Epub Date]|.
42. Yoo S, Hwang YS. Age-Group Related Cohort Effects on the Association between Age at Menarche and Metabolic Syndrome among Korean Premenopausal Women. Korean journal of family medicine 2019;**40**(4):280-81 doi: 10.4082/kjfm.19.0045[published Online First: Epub Date]|.
43. Akter S, Jesmin S, Islam M, et al. Association of age at menarche with metabolic syndrome and its components in rural Bangladeshi women. Nutr Metab (Lond) 2012;**9**(1):99 doi: 10.1186/1743-7075-9-99[published Online First: Epub Date]|.
44. Glueck CJ, Morrison JA, Wang P, et al. Early and late menarche are associated with oligomenorrhea and predict metabolic syndrome 26 years later. Metabolism: clinical and experimental 2013;**62**(11):1597-606 doi: 10.1016/j.metabol.2013.07.005[published Online First: Epub Date]|.
45. Kim Y, Je Y. Early Menarche and Risk of Metabolic Syndrome: A Systematic Review and Meta-Analysis. Journal of women's health 2019;**28**(1):77-86 doi: 10.1089/jwh.2018.6998[published Online First: Epub Date]|.
46. Farahmand M, Ramezani Tehrani F, Behboudi Gandevani S, et al. Is There any Association between Age at Menarche and Risk of Metabolic Syndrome? The Tehran Lipid & Glucose Study. Arch Iran Med 2019;**22**(9):495-500
47. Hwang YS, Park EJ, Choi JG, et al. Relationship between Age at Menarche and Metabolic Syndrome in Premenopausal Women: Korea National Health and Nutrition Examination Survey 2013-2014. Korean journal of family

For peer review only - http://bmjopen.bmj.com/site/about/guidelines.xhtml

medicine 2018;**39**(5):300-06 doi: 10.4082/kjfm.17.0022[published Online First: Epub Date]|.

48. Ahmed ML, Ong KK, Dunger DB. Childhood obesity and the timing of puberty. Trends Endocrinol Metab 2009;**20**(5):237-42 doi: 10.1016/j.tem.2009.02.004[published Online First: Epub Date]|.
49. Jasik CB, Lustig RH. Adolescent obesity and puberty: the "perfect storm". Ann N Y Acad Sci 2008;**1135**:265-79 doi: 10.1196/annals.1429.009[published Online First: Epub Date]|.
50. Garn SM. Continuities and changes in fatness from infancy through adulthood. Curr Probl Pediatr 1985;**15**(2):1-47 doi: 10.1016/0045-9380(85)90015-5[published Online First: Epub Date]|.
51. van Lenthe FJ, Kemper CG, van Mechelen W. Rapid maturation in adolescence results in greater obesity in adulthood: the Amsterdam Growth and Health Study. The American journal of clinical nutrition 1996;**64**(1):18-24 doi: 10.1093/ajcn/64.1.18[published Online First: Epub Date]|.
52. Remsberg KE, Demerath EW, Schubert CM, et al. Early menarche and the development of cardiovascular disease risk factors in adolescent girls: the Fels Longitudinal Study. The Journal of clinical endocrinology and metabolism 2005;**90**(5):2718-24 doi: 10.1210/jc.2004-1991[published Online First: Epub Date]|.
53. Feng Y, Hong X, Wilker E, et al. Effects of age at menarche, reproductive years, and menopause on metabolic risk factors for cardiovascular diseases. Atherosclerosis 2008;**196**(2):590-7 doi: 10.1016/j.atherosclerosis.2007.06.016[published Online First: Epub Date]|.
54. Won JC, Hong JW, Noh JH, et al. Association Between Age at Menarche and Risk Factors for Cardiovascular Diseases in Korean Women: The 2010 to 2013 Korea National Health and Nutrition Examination Survey. Medicine (Baltimore) 2016;**95**(18):e3580 doi: 10.1097/MD.0000000000003580[published Online First: Epub Date]|.
55. Au Yeung SL, Jiang C, Cheng KK, et al. Age at menarche and cardiovascular risk factors using Mendelian randomization in the Guangzhou Biobank Cohort Study. Preventive medicine 2017;**101**:142-48 doi: 10.1016/j.ypmed.2017.06.006[published Online First: Epub Date]|.
56. Ley SH, Li Y, Tobias DK, et al. Duration of Reproductive Life Span, Age at Menarche, and Age at Menopause Are Associated With Risk of Cardiovascular Disease in Women. J Am Heart Assoc 2017;**6**(11) doi: 10.1161/JAHA.117.006713[published Online First: Epub Date]|.
57. Luijken J, van der Schouw YT, Mensink D, et al. Association between age at menarche and cardiovascular disease: A systematic review on risk and potential mechanisms. Maturitas 2017;**104**:96-116 doi: 10.1016/j.maturitas.2017.07.009[published Online First: Epub Date]|.
58. Yang L, Li L, Millwood IY, et al. Age at menarche and risk of major cardiovascular diseases: Evidence of birth cohort effects from a prospective study of 300,000 Chinese women. International journal of cardiology 2017;**227**:497-502 doi: 10.1016/j.ijcard.2016.10.115[published Online First: Epub Date]|.
59. Chen X, Liu Y, Sun X, et al. Age at menarche and risk of all-cause and cardiovascular mortality: a systematic review and dose-response meta-analysis. Menopause 2018;**26**(6):670-76 doi: 10.1097/GME.0000000000001289[published Online First: Epub Date]|.
60. Lee JJ, Cook-Wiens G, Johnson BD, et al. Age at Menarche and Risk of Cardiovascular Disease Outcomes: Findings From the National Heart Lung and Blood Institute-Sponsored Women's Ischemia Syndrome Evaluation. J Am Heart Assoc 2019;**8**(12):e012406 doi: 10.1161/JAHA.119.012406[published Online First: Epub Date]|.
61. Zhang L, Li Y, Zhou W, et al. Mediation effect of BMI on the relationship between age at menarche and hypertension: The Henan Rural Cohort Study. Journal of human hypertension 2019 doi: 10.1038/s41371-019-0247-2[published Online First: Epub Date]|.
62. Werneck AO, Oyeyemi AL, Cyrino ES, et al. Association between age at menarche and blood pressure in adulthood: is obesity an important mediator? Hypertension research : official journal of the Japanese Society of Hypertension 2018;**41**(10):856-64 doi: 10.1038/s41440-018-0079-4[published Online First: Epub Date]|.
63. Lakshman R, Forouhi NG, Sharp SJ, et al. Early age at menarche associated with cardiovascular disease and mortality. The Journal of clinical endocrinology and metabolism 2009;**94**(12):4953-60 doi: 10.1210/jc.2009-1789[published Online First: Epub Date]|.

For peer review only - http://bmjopen.bmj.com/site/about/guidelines.xhtml

64. Charalampopoulos D, McLoughlin A, Elks CE, et al. Age at menarche and risks of all-cause and cardiovascular death: a systematic review and meta-analysis. American journal of epidemiology 2014;**180**(1):29-40 doi: 10.1093/aje/kwu113[published Online First: Epub Date]|.

65. Zheng Y, Zhang G, Chen Z, et al. Association between Age at Menarche and Cardiovascular Disease Risk Factors in China: A Large Population-Based Investigation. Cardiorenal Med 2016;**6**(4):307-16 doi: 10.1159/000445506[published Online First: Epub Date]|.

For peer review only

For peer review only - http://bmjopen.bmj.com/site/about/guidelines.xhtml

STROBE 2007 (v4) Statement—Checklist of items that should be included in reports of *cross-sectional studies*

Section/Topic	Item #	Recommendation	Reported on page #
**Title and abstract**	1	(*a*) Indicate the study's design with a commonly used term in the title or the abstract	1
		(*b*) Provide in the abstract an informative and balanced summary of what was done and what was found	2-3
**Introduction**			
Background/rationale	2	Explain the scientific background and rationale for the investigation being reported	4-5
Objectives	3	State specific objectives, including any prespecified hypotheses	5
**Methods**			
Study design	4	Present key elements of study design early in the paper	6
Setting	5	Describe the setting, locations, and relevant dates, including periods of recruitment, exposure, follow-up, and data collection	6
Participants	6	(*a*) Give the eligibility criteria, and the sources and methods of selection of participants	7
Variables	7	Clearly define all outcomes, exposures, predictors, potential confounders, and effect modifiers. Give diagnostic criteria, if applicable	7-8
Data sources/ measurement	8*	For each variable of interest, give sources of data and details of methods of assessment (measurement). Describe comparability of assessment methods if there is more than one group	8
Bias	9	Describe any efforts to address potential sources of bias	*8*
Study size	10	Explain how the study size was arrived at	7
Quantitative variables	11	Explain how quantitative variables were handled in the analyses. If applicable, describe which groupings were chosen and why	7
Statistical methods	12	(*a*) Describe all statistical methods, including those used to control for confounding	7-8
		(*b*) Describe any methods used to examine subgroups and interactions	7-8
		(*c*) Explain how missing data were addressed	7-8
		(*d*) If applicable, describe analytical methods taking account of sampling strategy	7-8
		(*e*) Describe any sensitivity analyses	7-8
**Results**			

For peer review only

Participants	13*	(a) Report numbers of individuals at each stage of study—eg numbers potentially eligible, examined for eligibility, confirmed eligible, included in the study, completing follow-up, and analysed	9-10
		(b) Give reasons for non-participation at each stage	9-10
		(c) Consider use of a flow diagram	9-10
Descriptive data	14*	(a) Give characteristics of study participants (eg demographic, clinical, social) and information on exposures and potential confounders	10-13
		(b) Indicate number of participants with missing data for each variable of interest	10-13
Outcome data	15*	Report numbers of outcome events or summary measures	10-13
Main results	16	(*a*) Give unadjusted estimates and, if applicable, confounder-adjusted estimates and their precision (eg, 95% confidence interval). Make clear which confounders were adjusted for and why they were included	13-16
		(*b*) Report category boundaries when continuous variables were categorized	13-16
		(*c*) If relevant, consider translating estimates of relative risk into absolute risk for a meaningful time period	13-16
Other analyses	17	Report other analyses done—eg analyses of subgroups and interactions, and sensitivity analyses	
**Discussion**			
Key results	18	Summarise key results with reference to study objectives	17-19
Limitations	19	Discuss limitations of the study, taking into account sources of potential bias or imprecision. Discuss both direction and magnitude of any potential bias	17-19
Interpretation	20	Give a cautious overall interpretation of results considering objectives, limitations, multiplicity of analyses, results from similar studies, and other relevant evidence	17-19
Generalisability	21	Discuss the generalisability (external validity) of the study results	17-19
**Other information**			
Funding	22	Give the source of funding and the role of the funders for the present study and, if applicable, for the original study on which the present article is based	20

*Give information separately for cases and controls in case-control studies and, if applicable, for exposed and unexposed groups in cohort and cross-sectional studies.

Note: An Explanation and Elaboration article discusses each checklist item and gives methodological background and published examples of transparent reporting. The STROBE checklist is best used in conjunction with this article (freely available on the Web sites of PLoS Medicine at http://www.plosmedicine.org/, Annals of Internal Medicine at http://www.annals.org/, and Epidemiology at http://www.epidem.com/). Information on the STROBE Initiative is available at www.strobe-statement.org.

For peer review only

BMJ Open

Mediator or moderator? The role of obesity in the association between age at menarche and blood pressure in mid-aged and elderly Chinese: a population-based cross-sectional study

Journal:	*BMJ Open*
Manuscript ID	bmjopen-2021-051486.R1
Article Type:	Original research
Date Submitted by the Author:	07-Dec-2021
Complete List of Authors:	zhang, lin; Wannan Medical College, Department of Internal Medicine Nursing, School of Nursing, Wannan Medical College, 22 Wenchang West Road, Higher Education Park, Wuhu City, An Hui Province, P.R.China<br>Yang, Liu; Wannan Medical College, Department of Internal Medicine Nursing, School of Nursing, Wannan Medical College, 22 Wenchang West Road, Higher Education Park, Wuhu City, An Hui Province, P.R.China<br>Wang, Congzhi; Wannan Medical College, Department of Internal Medicine Nursing, School of Nursing, Wannan Medical College, 22 Wenchang West Road, Higher Education Park, Wuhu City, An Hui Province, P.R.China<br>Yuan, Ting; Wannan Medical College, Obstetrics and Gynecology Nursing, School of Nursing, Wannan Medical College, 22 Wenchang West Road, Higher Education Park, Wuhu City, An Hui Province, P.R.China<br>Zhang, Dongmei; Wannan Medical College, Department of Pediatric Nursing, School of Nursing, Wannan Medical College, 22 Wenchang West Road, Higher Education Park, Wuhu City, An Hui Province, P.R.China<br>Wei, Huanhuan; Wannan Medical College, Obstetrics and Gynecology Nursing, School of Nursing, Wannan Medical College, 22 Wenchang West Road, Higher Education Park, Wuhu City, An Hui Province, P.R.China<br>Li, Jing; Wannan Medical College, Department of Surgical Nursing, School of Nursing, Wannan Medical College, 22 Wenchang West Road, Higher Education Park, Wuhu City, An Hui Province, P.R.China<br>Lei, Yunxiao; Wannan Medical College, Obstetrics and Gynecology Nursing, School of Nursing, Wannan Medical College, 22 Wenchang West Road, Higher Education Park, Wuhu City, An Hui Province, P.R.China; Henan University of Science and Technology, School of Nursing<br>Sun, Lu; Wannan Medical College, Department of Emergency and Critical Care Nursing, School of Nursing, Wannan Medical College, 22 Wenchang West Road, Higher Education Park, Wuhu City, An Hui Province, P.R.China<br>Li, Xiaoping; Wannan Medical College, Department of Emergency and Critical Care Nursing, School of Nursing, Wannan Medical College, 22 Wenchang West Road, Higher Education Park, Wuhu City, An Hui Province, P.R.China<br>Hua, Ying; Wannan Medical College, Rehabilitation Nursing, School of Nursing, Wanna Medical college, 22 Wenchang West Road, Higher Education Park, Wuhu City, An Hui Province, P.R.China<br>Che, Hengying; Yijishan Hospital of Wannan Medical College, Department

	of nursing, Yijishan Hospital, the First Affiliated Hospital of Wannan Medical College, Zheshan West Road, Yijishan District, Wuhu City, Anhui Province<br>Li, Yuanzhen; Wannan Medical College, Department of Emergency and Critical Care Nursing, School of Nursing, Wannan Medical College, 22 Wenchang West Road, Higher Education Park, Wuhu City, An Hui Province, P.R.China
**Primary Subject Heading**:	Geriatric medicine
Secondary Subject Heading:	Public health
Keywords:	Hypertension < CARDIOLOGY, Risk management < HEALTH SERVICES ADMINISTRATION & MANAGEMENT, Nutrition < TROPICAL MEDICINE

I, the Submitting Author has the right to grant and does grant on behalf of all authors of the Work (as defined in the below author licence), an exclusive licence and/or a non-exclusive licence for contributions from authors who are: i) UK Crown employees; ii) where BMJ has agreed a CC-BY licence shall apply, and/or iii) in accordance with the terms applicable for US Federal Government officers or employees acting as part of their official duties; on a worldwide, perpetual, irrevocable, royalty-free basis to BMJ Publishing Group Ltd ("BMJ") its licensees and where the relevant Journal is co-owned by BMJ to the co-owners of the Journal, to publish the Work in this journal and any other BMJ products and to exploit all rights, as set out in our licence.

The Submitting Author accepts and understands that any supply made under these terms is made by BMJ to the Submitting Author unless you are acting as an employee on behalf of your employer or a postgraduate student of an affiliated institution which is paying any applicable article publishing charge ("APC") for Open Access articles. Where the Submitting Author wishes to make the Work available on an Open Access basis (and intends to pay the relevant APC), the terms of reuse of such Open Access shall be governed by a Creative Commons licence – details of these licences and which Creative Commons licence will apply to this Work are set out in our licence referred to above.

Other than as permitted in any relevant BMJ Author's Self Archiving Policies, I confirm this Work has not been accepted for publication elsewhere, is not being considered for publication elsewhere and does not duplicate material already published. I confirm all authors consent to publication of this Work and authorise the granting of this licence.

For peer review only

Mediator or moderator? The role of obesity in the association between age at menarche and blood pressure in mid-aged and elderly Chinese: a population-based cross-sectional study

Lin Zhang[1], Liu Yang[1], Congzhi Wang[1], Ting Yuan[2], Dongmei Zhang[3], Huanhuan Wei[2], Jing Li[4], Yunxiao Lei[2], Lu Sun[5], Xiaoping Li[5], Ying Hua[6], Hengying Che[7*], Yuanzhen Li[5*]

*Correspondence: Hengying Che: 2722659079@qq.com and Yuanzhen Li: yuanzhen.li@wnmc.edu.cn

Yuanzhen Li and Hengying Che contributed equally to this work.

Lin Zhang: yaoran2008@163.com

Liu Yang: zqzqylyl5858@163.com

Congzhi Wang: 289957420@qq.com

Ting Yuan: yuanting@wnmc.edu.cn

Dongmei Zhang: dongmei.0715@163.com

Huanhuan Wei: whh0702@163.com

Jing Li: 475366616@qq.com

Yunxiao Lei: lyxheart@163.com

Lu Sun: 565196200@qq.com

Xiaoping Li：349274808@qq.com

Ying Hua: 1542997737@qq.com

Hengying Che: 2722659079@qq.com

Yuanzhen Li: yuanzhen.li@wnmc.edu.cn

[1] Department of Internal Medicine Nursing, School of Nursing, Wannan Medical College, 22 Wenchang West Road, Higher Education Park, Wuhu City, An Hui Province, P.R.China

[2] Obstetrics and Gynecology Nursing, School of Nursing, Wannan Medical College, 22 Wenchang West Road, Higher Education Park, Wuhu City, An Hui Province, P.R.China

[3] Department of Pediatric Nursing, School of Nursing, Wannan Medical College, 22 Wenchang West Road, Higher Education Park, Wuhu City, An Hui Province, P.R.China

[4] Department of Surgical Nursing, School of Nursing, Wannan Medical College, 22 Wenchang West Road, Higher Education Park, Wuhu City, An Hui Province, P.R.China

[5] Department of Emergency and Critical Care Nursing, School of Nursing, Wannan Medical College, 22 Wenchang West Road, Higher Education Park, Wuhu City, An Hui Province, P.R.China

[6] Rehabilitation Nursing, School of Nursing, Wanna Medical college, 22 Wenchang West Road, Higher Education Park, Wuhu City, An Hui Province, P.R.China

[7] Department of nursing, Yijishan Hospital, the First Affiliated Hospital of Wannan Medical College, Zheshan West Road, Yijishan District, Wuhu City, Anhui Province

For peer review only - http://bmjopen.bmj.com/site/about/guidelines.xhtml

For peer review only

ABSTRACT

Objective We investigated the moderation/mediation between the age of menarche and obesity parameters in predicting blood pressure (BP) in middle-aged and elderly Chinese.

Design Our study is a population-based cross-sectional study.

Setting Participants in this study came from the China Health and Retirement Longitudinal Study (CHARLS).

Participants The analytic sample included 4513 participants aged 45 to 96 years.

Main outcome measurements Data were selected from the CHARLS, a cross-sectional study. Between-group differences were evaluated using chi-square, t-test and one-way ANOVA. The trend of related variables by characteristics was also tested using contrast analysis, as appropriate. Then, correlations between characteristics, moderator, mediator, independent, and dependent variables were used by Spearman's correlation test and Pearson's correlation test. Finally, the mediation analysis was performed by model 4 in process v3.3, and the moderation analysis was used model 1 to assess. All covariates were adjusted in the moderation or mediation models.

Results In correlation analysis, body mass index (BMI) and waist circle (WC) level were positively correlated with both systolic blood pressure (SBP) and diastolic blood pressure (DBP) in women (BMI and DBP: r =0.221, *P* <0.001; WC and DBP: r =0.183, *P* <0.001; BMI and SBP: r =0.129, *P* <0.001; WC and SBP: r =0.177, *P* <0.001). Age of menarche was negatively correlated with DBP (r =-0.060, *P* <0.001). However, the age of menarche was not significantly correlated with SBP (r =-0.014, *P* =0.335). In the moderator analysis, after controlling for the potential confounders, the interaction term of obesity parameters*age of menarche was not significant for predicting either DBP (BMI: B = 0.0260, SE = 0.0229, *P* =0.2556, 95% CI [-0.0189,0.071]); WC: B = 0.0099, SE =0.0074, *P* =0.1833, 95% CI [-0.0047,0.0244]) or SBP (BMI: B =0.0091, SE =0.0504, *P* =0.8561, 95% CI [-0.0897,0.108]); WC: B = -0.0032, SE =0.0159, *P* =0.8427, 95% CI [-0.0343,0.028]). All correlations were significant correlation between age of menarche, obesity parameters and BP except the path of the menarche age →SBP (with the addition of the BMI indicator: β =-0.0004, B =-0.0046, *P* =0.9797, 95% CI [-0.3619,0.3526]); with the addition of the WC indicator: β = 0.0004, B =0.0044, *P* =0.9804, 95% CI [-0.3439, 0.3526]) in crude model. In general, after controlling for potential confounders. BMI (DBP: β = -0.0471, B= -0.2682, *P* =0.0021, 95% CI [-0.4388, -0.0976]; SBP: β =-0.0515, B =-0.6314, *P* <0.001, 95% CI [-0.9889, -0.2739]) and WC (DBP: β = -0.0474, B= -0.2689, *P* <0.001, 95% CI [-0.4395, -0.0984]; SBP: β =-0.0524, B =-0.632, *P* <0.001, 95% CI [-0.9832, -0.2807]) partly

For peer review only - http://bmjopen.bmj.com/site/about/guidelines.xhtml

mediated the relationship between age of menarche and BP.

Conclusion The interaction term of obesity parameters*age of menarche was not significant for predicting either DBP or SBP in women. Moreover, obesity parameters partly mediated the relationship between the age of menarche and BP.

Keywords age at menarche, blood pressure, body mass index, moderator, mediator, obesity parameters, waist circle

Strengths and limitations of the study

This is the first large population study to examine the moderation between the age of menarche and obesity parameters in predicting blood pressure (BP) in middle-aged and elderly Chinese, as well as the mediation effects of obesity parameters on the relationship between age of menarche and BP.

This study included a large sample of 4513 middle-aged and older Chinese. The results presented in this article represent the baseline data that could be explored further in prospective cohort studies.

The main limitations of our research are related to the cross-sectional study and the self-reported method used for the assessment of age at menarche and most related confounders.

Blood pressure in our study was measured at home by professionally trained volunteers, and the next step is the use of clinical blood pressure and ambulatory blood pressure.

For peer review only

For peer review only - http://bmjopen.bmj.com/site/about/guidelines.xhtml

INTRODUCTION

Blood pressure (BP) is the force of circulating blood against the walls of the body's arteries, the major blood vessels in the body. Hypertension is diagnosed if BP readings are 140/90 mmHg or above on two different days. As a chronic disease[1], hypertension or elevated BP is a severe medical condition that significantly increases heart attack, stroke, blindness, kidney failure, and other complications. Hypertension risk factors[2-6] include stress, salt consumption, harmful use of alcohol, low intake of fruits and vegetables, saturated fat and trans fats, being overweight or obese, tobacco use, low diet in vitamin D, lack of physical activity, family history, aged 65 years or over and other co-existing diseases. The World Health Organization(WHO) said[7] an estimated 1.13 billion people worldwide have hypertension and most (two-thirds) living in low- and middle-income countries. It means that most people have high BP. Compared to the global average, people in low- and middle-income countries may bear a disproportionate and heavier burden of the disease, owing to several factors such as the ongoing nutritional transition in dietary consumption and energy expenditure, increasing trends in sedentary lifestyle, and other modifiable risk factors, and inadequate health care systems[8]. As a developing country, China has shown a relatively high and stable prevalence of hypertension among adults, especially in middle-aged and older people. Mahajan S, *et al.* [9] showed the prevalence of hypertension among middle-aged and elderly Chinese was approximately 41.81% in 2015. However, the rate control was still below 30%. Recently, hypertension control has become one of the critical public health interventions[10]. Therefore, it emphasizes the promotion of public health strategy to prevent the significant hypertension risk factors and assure the fullest available accessibility and use of quality health control for people with risk factors or who develop subclinical or overt hypertension. These actions are integral to a comprehensive public health strategy for hypertension promotion and prevention[11-13]. Though there is still much uncertainty about the etiology of hypertension, one of the strongest risk factors was overweight/obesity. Thus, increased body mass index (BMI) or centrally located body fat (especially waist circle [WC]) increases the risk of developing hypertension.

Several studies[14-18]have reported that early biological maturation has been associated with obesity and BP in adolescents. Werneck AO *et al.* [14] showed that behavioral and hereditary variables are more related to BP in late-maturing adolescents in adolescents. Widen E, *et al.*[15] found that earlier pubertal timing was associated with higher adult BMI and diastolic blood pressure (DBP) in both sexes. Mueller NT, *et al.* [16] showed that earlier menarche was associated with higher waist circumference and BMI measured. Dreyfus J, *et al.*[17] found that earlier age at menarche was

For peer review only - http://bmjopen.bmj.com/site/about/guidelines.xhtml

associated with higher mean BMI among African American and White women. Cao M, *et al.*[18] found that earlier age at menarche was associated with decreased DBP. However, it is not completed understood whether the changes in biological processes in the early years can affect cardiovascular outcomes across the life span. As important indicators of obesity, BMI/WC is directly associated with biological maturation (based on menarche age). Evidence from several studies[19-21] indicated that menarche was related to BMI in later life. Power C, *et al.*[19] found that timing of puberty was strongly associated with BMI for the earlier matures at ages 7-33 years. Adair LS, *et al.*[20] showed that overweight prevalence rates were significantly higher in early maturing adolescents. Must A, *et al.*[21] showed that early maturation in adult females overweight is mostly a result of the influence of elevated relative weight on early maturation. It has been speculated that the association between biological maturation and hypertension risk factors (based on menarche age) can be mediated or moderated by obesity parameters. Although speculative, the mediating or moderating role of obesity on the effect of early maturation (based on menarche age) on BP in adulthood has not been classified. However, this is especially important given that the onset of biological maturation, especially the age of menarche, now occurs at a much earlier age than in previous studies. Moreover, fully understanding the mediating effect of obesity on the association between early maturation (based on menarche age) and BP in adulthood could help identify women group that are at risk (based on obesity parameters) and those that should be put forward designed to place the many diverse conceptual and practice approaches and accomplishments in the early intervention. The moderating effect between early maturation (based on menarche age) and risk of hypertension (obesity parameters) on BP may also be fully considered.

To date, few studies on the mediating and moderating analysis between early maturation (based on menarche age) and risk of hypertension (obesity parameters) were conducted in individuals aged ≥45 years. Thus, this study aimed to determine their association with age of menarche, obesity parameters, and other confounding factors using the China Health and Retirement Longitudinal Study (CHARLS) from participants aged ≥45 years in China.

METHODS

Study design and setting

Data from CHARLS Wave 1 (2011) were used in our research. The CHARLS is an ongoing national longitudinal study administered by the National School for Development (China Center for Economic

For peer review only - http://bmjopen.bmj.com/site/about/guidelines.xhtml

Research) [22] from 2011. The study was approved by the institutional ethical committees of Peking University. All participants provided informed consent.

Individuals

At baseline[22], 6883 women were recruited for a longitudinal study, 1875 individuals were excluded because the absence of metabolic measures, a group of 41 participants did not have medication history, and 65 individuals did not have their blood pressure, 389 individuals used antihypertension drugs. Finally, 4513 individuals were included in the analyses. Figure 1 summarized the selection of participants. Our study involved 4513 participants aged ≥45 years were total women [mean ± standard deviation age=58.59 ± 9.31years, ranged from 45 to 96years]. The mean and standard deviation of menarche age were 16.27±2.18 years (ranged from 10 to 30 years).

Self-report of risk factors

We employed a general sociodemographic questionnaire that included the age, age of menarche (≤14years, 15years, 16years, 17years, and ≥18years), educational attainment (illiterate, less than elementary school, high school, and above vocational school), marital status (single and married), and region (rural and urban). The lifestyle questionnaire included smoking status (never smoker, previous smoker, and current smoker), alcohol consumption (never drinker, less than once a month and more than once a month), eating habit (≤2 meals per day, 3 meals per day, and ≥4 meals per day), entertainment, the experience of a traumatic event (no and yes), and physical exercise habit (no, less than regular physical exercises, and regular physical exercises). History of cardiovascular diseases and liver disease, antihyperlipidemic drugs, and antidiabetic drugs were categorized in 'yes' or 'no'. Most variables were used in our previous studies[23-29].

Measurements

Triglycerides (TG) level, low-density lipoprotein cholesterol (LDL-C), high-density lipoprotein cholesterol (HDL-C), and glycosylated hemoglobin (HbA1c) were analyzed by the enzymatic colorimetric tests, serum creatinine (Scr) and serum uric acid (SUA) level were analyzed using the urinalysis (UA) plus method. The average value of BP was measured by the mean of the 3-time measurements. Height was measured using a height measurement instrument (Omron hem-7200 sphygmomanometer). Participants were asked to stand barefoot on the base plate of the height meter

For peer review only - http://bmjopen.bmj.com/site/about/guidelines.xhtml

in the upright position, with the back against the vertical backplate of the measuring instrument, with eyes open, arms alongside the body and heels were closed and the toes were separated by 60 degrees. Anyone who appeared to unable to complete measurement in time due to health reasons such as hunchback and inability to stand was excluded from our study. The method was used in our previous study[29]. BMI was calculated as body weight (kg) divided by the square of the subject's height (m). WC was measured midway between spina iliaca superior and the lower rib margin at the end of exhalation.

Statistical analysis

The data were presented as means(M) and standard deviation (SD) unless indicated otherwise. Mean and standard deviation were used to describe continuous variables (age, age of menarche, LDL-C, HDL-C, TG, HbA1c, Scr, SUA, BMI, WC, DBP, and SBP level), and number and percentage were used to assess the categorical variables (educational attainment, marital status, region, alcohol consumption, smoking status, eating habits, entertainment, the experience of a traumatic event, physical activity, history of CVDs, history of liver diseases, history of antilipidemic medication, anti-diabetic medication). Between-group differences according to the age of menarche (≤14years, 15years, 16years, 17years, ≥18years) were evaluated by the ANOVA (continuous data) or chi-square test (categorical data). Differences between two categories of characteristics (marital status, region, entertainment, the experience of a traumatic event, history of CVDs, history of antilipidemic medication, history of liver diseases, anti-diabetic medication) were also evaluated using t-test. However, the trend of related variables (LDL-C, HDL-C, LDL-C/HDL-C, TG, HbA1c, Scr, SUA, BMI, WC, DBP, and SBP level) by above three categories of characteristics (age of menarche levels, educational attainment, alcohol consumption, smoking status, eating habits, physical activity) was also tested using contrast analysis. Correlations between characteristics and BP were used by Spearman's correlation test. However, relationships between continuous variables (age, LDL-C, HDL-C, LDL-C/HDL-C, TG, HbA1c, Scr, SUA, BMI, WC and menarche age) and BP were used by Pearson's correlation test. The mediation analysis was performed by model 4 in process v3.3[30-32], and the moderation analysis was used model 1 to assess. All covariates (including age, educational attainment, marital status, region, smoking status, alcohol consumption, eating habits, entertainment, the experience of a traumatic event, physical activity, history of cardiovascular diseases, hepatitis history, antihyperlipidemic drugs, antidiabetic drugs, LDL-C, HDL-C, TG, HbA1c, Scr, SUA) were adjusted for in the mediation or moderation models. Analyses including the unstandardized (B),

For peer review only - http://bmjopen.bmj.com/site/about/guidelines.xhtml

Standard Error (SE), or standardized coefficients (β) with 95% confidence intervals(95%CI) were calculated using the setting of modelling building in IBM SPSS22.0 software for Windows10 (IBM Corp., Armonk, NY, USA) using the bootstrap method, with a 5% significance level.

Patient and public involvement

No patients were involved in the development of the question, design or data interpretation.

For peer review only

For peer review only - http://bmjopen.bmj.com/site/about/guidelines.xhtml

RESULTS

The final sample consisted of 4513 women with an average age of 58.59 ± 9.31years (ranged from 45 to 96 years). The mean and standard deviation of menarche age were 16.27±2.18 years (ranged from 10 to 30 years). The mean and standard deviation of DBP and SBP level were 76.31±12.45mmHg (ranged from 39 to 136 mmHg), and 131.65±26.73 mmHg (ranged from 76 to 235 mmHg), respectively. The mean and standard deviation of BMI and WC level were 24.04±4.13 kg/m^2(ranged from 11.65 to 63.05 kg/m^2), and 84.67±12.83cm (ranged from 19.5 to 130.10 cm), respectively. Table 1 showed the characteristics by the age of menarche. There were differences between the age of menarche in educational attainment, region, cigarette smoking, entertainment, and history of CVDs. However, between-group differences in the prevalence of marital status, alcohol consumption, eating habit, the experience of a traumatic event, physical activity, hepatitis history, antilipidemic medication history, and antidiabetic medication history were not found. Women with a higher age of menarche had higher HDL-C level and age but had lower TG, SUA, BMI, WC, and DBP level. Moreover, there were no differences in LDL-C, LDL-C/HDL-C, HbA1c, Scr, and SBP level between the age of menarche groups.

Table1 Characteristics by age at menarche (N=4513)

Variables	≤14(years) N=946	15(years) N=671	16(years) N=930	17(years) N=702	≥18(years) N=1264	*P*
Age(years)	56.88±9.38	57.49±9.34	58.41±9.42	58.6±9.12	60.57±8.91	<0.001[a]
Educational attainment						
Illiterate	300(31.71)	233(34.72)	378(40.65)	314(44.73)	649(51.34)	<0.001
Less than elementary school	527(55.71)	383(57.08)	486(52.26)	343(48.86)	572(45.25)	
High school	82(8.67)	42(6.26)	48(5.16)	34(4.84)	34(2.69)	
Above vocational school	37(3.91)	13(1.94)	18(1.94)	11(1.57)	9(0.71)	
Marital status						
Single	137(14.48)	79(11.77)	137(14.73)	98(13.96)	207(16.38)	0.101
Married	809(85.52)	592(88.23)	793(85.27)	604(86.04)	1057(83.62)	
Region						
Rural	519(54.86)	426(63.49)	577(62.04)	454(64.67)	885(70.02)	<0.001
Urban	427(45.14)	245(36.51)	353(37.96)	248(35.33)	379(29.98)	
Cigarette smoking						
Never smoker	883(93.34)	629(93.74)	846(90.97)	646(92.02)	1156(91.46)	0.017
Previous smoker	8(0.85)	11(1.64)	18(1.94)	13(1.85)	38(3.01)	
Current smoker	55(5.81)	31(4.62)	66(7.1)	43(6.13)	70(5.54)	
Alcohol consumption						
Never drinker	827(87.42)	607(90.46)	809(86.99)	620(88.32)	1100(87.03)	0.520
Less than once a month	45(4.76)	26(3.87)	48(5.16)	37(5.27)	65(5.14)	
More than once a month	74(7.82)	38(5.66)	73(7.85)	45(6.41)	99(7.83)	
Eating habit						
≤2 meals per day	131(13.85)	99(14.75)	123(13.23)	82(11.68)	172(13.61)	0.706
3 meals per day	804(84.99)	567(84.5)	794(85.38)	609(86.75)	1073(84.89)	
≥4 meals per day	11(1.16)	5(0.75)	13(1.40)	11(1.57)	19(1.50)	
Entertainment						
No	417(44.08)	335(49.93)	470(50.54)	372(52.99)	655(51.82)	0.002
Yes	529(55.92)	336(50.07)	460(49.46)	330(47.01)	609(48.18)	
The experience of a traumatic event						
No	881(93.13)	630(93.89)	867(93.23)	652(92.88)	1169(92.48)	0.837
Yes	65(6.87)	41(6.11)	63(6.77)	50(7.12)	95(7.52)	
Physical activity						
No physical exercise	587(62.05)	393(58.57)	556(59.78)	428(60.97)	771(61.00)	0.260
Less than regular physical exercises	178(18.82)	137(20.42)	171(18.39)	136(19.37)	271(21.44)	
Regular physical exercises	181(19.13)	141(21.01)	203(21.83)	138(19.66)	222(17.56)	
History of CVDs						
No	810(85.62)	590(87.93)	780(83.87)	605(86.18)	1115(88.21)	0.033
Yes	136(14.38)	81(12.07)	150(16.13)	97(13.82)	149(11.79)	

For peer review only - http://bmjopen.bmj.com/site/about/guidelines.xhtml

Hepatitis history						
No	910(96.19)	647(96.42)	885(95.16)	670(95.44)	1226(96.99)	0.205
Yes	36(3.81)	24(3.58)	45(4.84)	32(4.56)	38(3.01)	
History of antilipidemic medication						
No	884(93.45)	628(93.59)	884(95.05)	656(93.45)	1184(93.67)	0.566
Yes	62(6.55)	43(6.41)	46(4.95)	46(6.55)	80(6.33)	
History of antidiabetic medication						
No	910(96.19)	645(96.13)	883(94.95)	663(94.44)	1213(95.97)	0.309
Yes	36(3.81)	26(3.87)	47(5.05)	39(5.56)	51(4.03)	
LDL-C (mg/dL)	119.66±35.04	118.04±35.61	120.62±34.81	121.79±36.79	121.45±35.27	0.065 [a]
HDL-C (mg/dL)	50.57±14.17	51.31±14.01	50.84±14.43	51.05±14.18	53.2±14.87	<0.001 [a]
LDL-C/HDL-C	2.50±0.90	2.42±0.89	2.52±0.91	2.51±0.92	2.43±0.92	0.242 [a]
TG (mg/dL)	143.09±99.89	142.86±103.67	142.07±98.31	145.85±132.3	124.16±76.25	<0.001 [a]
HbA1c (%)	5.31±0.83	5.25±0.86	5.31±0.89	5.32±0.84	5.27±0.76	0.594 [a]
Scr (mg/dL)	0.69±0.14	0.69±0.14	0.70±0.18	0.68±0.13	0.69±0.15	0.813 [a]
SUA (mg/dL)	4.09±1.10	3.99±1.09	4.04±1.07	4.01±1.01	3.97±1.05	0.021 [a]
BMI (kg/m^2)	24.63±4.11	24.39±4.15	24.02±3.90	24.18±4.49	23.36±3.99	<0.001 [a]
WC (cm)	86.08±13.01	85.35±12.62	85.29±11.33	84.53±13.38	82.89±13.36	<0.001 [a]
DBP (mmHg)	77.07±12.07	77.28±12.53	77.01±12.51	76.31±12.80	74.72±25.82	<0.001 [a]
SBP (mmHg)	131.09±26.36	131.66±25.49	131.63±26.64	129.90±29.9	129.47±25.82	0.062 [a]

CVDs: cardiovascular diseases; LDL-C: low-density lipoprotein cholesterol; HDL-C: high-density lipoprotein cholesterol; TG: triglycerides; HbA1c: Hemoglobin A1C; Scr: serum creatinine; SUA: serum uric acid; BMI: body mass index; WC: waist circle; DBP: diastolic blood pressure; SBP: systolic blood pressure.

[a]*P*-value for trend

Characteristics, according to BMI and WC in women, were presented in Table 2. Firstly, women with the place in rural, more alcohol consumption, higher age of menarche had lower BMI and WC level; Secondly, women with a history of CVDs, antilipidemic medication, and antidiabetic medication had higher BMI and WC level than women without medical illness. Thirdly, women with higher educational attainment, being married, being never smoking, taking more eating meals, and taking entertainment had higher BMI level. At last, women with the experience of a traumatic event had lower WC level.

Table2 Characteristics of the participants with BMI and WC (N=4513)

Variables	BMI (cm)	WC (cm)	*P*-value for BMI	*P*-value for WC
Educational attainment				
Illiterate	23.57±4.19	84.35±12.95	0.001 [a]	0.971 [a]
Less than elementary school	24.31±4.03	85.14±12.48		
High school	24.93±4.07	83.17±14.65		
Above vocational school	24.85±4.43	83.40±13.99		
Marital status				
Single	23.21±4.18	84.93±12.04	<0.001	0.582
Married	24.19±4.10	84.63±12.97		
Region				
Rural	23.64±3.96	83.95±12.42	<0.001	<0.001
Urban	24.75±4.32	85.94±13.43		
Cigarette smoking				
Never smoker	24.11±4.12	84.73±12.77	<0.001 [a]	0.135 [a]
Previous smoker	23.79±4.14	86.83±10.36		
Current smoker	23.17±4.11	83.13±14.40		
Alcohol consumption				
Never drinker	24.10±4.14	85.02±12.5	0.011 [a]	<0.001 [a]
Less than once a month	23.88±4.17	81.78±15.16		
More than once a month	23.51±3.94	82.49±14.62		
Eating habit				
≤2 meals per day	23.27±3.89	84.12±10.42	<0.001 [a]	0.734 [a]
3 meals per day	24.18±4.14	84.82±13.18		
≥4 meals per day	23.10±4.69	80.82±12.01		
Entertainment				
No	23.64±4.09	84.34±12.08	<0.001	0.085
Yes	24.45±4.13	85.00±13.53		
The experience of a traumatic event				
No	24.06±4.14	84.82±12.57	0.474	0.004
Yes	23.88±3.94	82.64±15.81		
Physical activity				
No physical exercise	24.05±4.29	84.79±12.74	0.400 [a]	0.701 [a]
Less than regular physical exercises	23.82±3.70	84.25±12.48		
Regular physical exercises	24.26±4.03	84.74±13.48		

For peer review only

For peer review only - http://bmjopen.bmj.com/site/about/guidelines.xhtml

History of CVDs				
No	23.92±4.09	84.26±12.63	<0.001	<0.001
Yes	24.83±4.28	87.25±13.89		
Hepatitis history				
No	24.04±4.15	84.66±12.79	0..871	0.948
Yes	24.09±3.47	84.73±14.37		
History of antilipidemic medication				
No	23.87±3.99	84.22±12.72	<0.001	<0.001
Yes	26.75±5.14	91.59±12.58		
History of antidiabetic medication				
No	23.95±4.01	84.42±12.81	<0.001	<0.001
Yes	26.13±5.81	90.18±12.20		
Menarche age(years)				
≤14	24.63±4.11	86.08±13.01	<0.001 [a]	<0.001 [a]
15	24.39±4.15	85.35±12.62		
16	24.02±3.90	85.29±11.33		
17	24.18±4.49	84.53±13.38		
≥18	23.36±13.36	82.89±13.36		

CVDs: cardiovascular diseases; BMI: body mass index; WC: waist circle.

[a]*P*-value for trend

Characteristics, according to DBP and SBP in women, were presented in Table 3. Firstly, women with more alcohol consumption had lower DBP and SBP level; Secondly, women with a history of CVDs and antilipidemic medication had higher DBP and SBP level than women without medical illness. Thirdly, women with more eating meals, higher age of menarches had lower DBP level. At last, women with higher educational attainment, being married, and having regular physical exercises had lower SBP level, but women with antidiabetic medication had higher SBP level than women without medical illness.

Table3 Table2 Characteristics of the participants with DBP and SBP (N=4513)

**Variables**	**DBP(mmHg)**	**SBP(mmHg)**	***P*-value for DBP**	***P*-value for SBP**
Educational attainment				
Illiterate	76.37±12.45	133.93±28.43	0.288[a]	<0.001[a]
Less than elementary school	76.38±12.42	128.89±24.99		
High school	76.12±12.65	122.98±18.58		
Above vocational school	73.86±12.47	127.84±40.38		
Marital status				
Single	76.70±11.96	139.61±31.93	0.388	<0.001
Married	76.25±12.53	129.12±25.42		
Region				
Rural	76.20±12.72	130.18±25.92	0.435	0.121
Urban	76.50±11.96	131.46±28.06		
Cigarette smoking				
Never smoker	76.32±12.45	130.43±26.97	0.673[a]	0.051[a]
Previous smoker	73.60±11.41	132.13±22.83		
Current smoker	77.09±12.65	133.61±23.78		
Alcohol consumption				
Never drinker	76.53±12.38	131.27±27.27	0.003[a]	0.001[a]
Less than once a month	74.76±12.18	123.81±19.94		
More than once a month	74.73±13.26	127.77±23.06		
Eating habits				
≤2 meals per day	77.69±12.60	132.14±26.68	<0.001 [a]	0.117 [a]
3 meals per day	76.17±12.41	130.44±26.37		
≥4 meals per day	71.56±11.46	128.63±44.41		
Entertainment				
No	76.08±12.60	130.66±26.33	0.216	0.975
Yes	76.54±12.30	130.64±27.12		
The experience of a traumatic event				
No	76.38±12.47	130.84±27.12	0.219	0.075
Yes	75.48±12.14	128.05±20.66		
Physical activity				
No physical exercise	76.34±12.46	131.47±28.25	0.873 [a]	0.024 [a]
Less than regular physical exercises	76.05±12.76	129.23±22.14		
Regular physical exercises	76.50±12.08	129.54±26.03		
History of CVDs				
No	76.11±12.44	130.02±26.29	0.021	<0.001

For peer review only

For peer review only - http://bmjopen.bmj.com/site/about/guidelines.xhtml

Yes	77.39±12.40	134.50±29.28		
Hepatitis history				
No	76.36±12.45	130.76±26.50	0.017	0.144
Yes	74.10±11.56	127.76±32.79		
History of antilipidemic medication				
No	76.07±12.34	130.12±26.01	<0.001	<0.001
Yes	80.09±13.46	138.80±35.01		
History of antidiabetic medication				
No	76.25±12.47	130.17±25.46	0.100	<0.001
Yes	77.73±11.83	140.96±45.17		
Menarche age(years)				
≤14	77.07±12.07	131.09±26.36		
15	77.28±12.53	131.66±25.49	<0.001 [a]	0.062 [a]
16	77.01±12.51	131.63±26.64		
17	76.31±12.80	129.90±29.90		
≥18	74.72±12.31	129.47±25.82		

CVDs: cardiovascular diseases; DBP: diastolic blood pressure; SBP: systolic blood pressure.

[a]*P*-value for trend

Correlations between characteristics, independent, moderators, mediators and dependent variables, according to DBP and SBP, were presented in Table 4. Firstly, drinking, history of CVDs, antilipidemic medication, LDL-C, HDL-C, LDL-C/HDL-C, TG, HbA_1c, Scr, SUA, BMI, and WC were significantly correlated with DBP and SBP in women; Secondly, eating habits, history of liver diseases, and menarche age were significantly correlated with DBP; At last, age, educational attainment, marital status, physical exercises, and antidiabetic medication were significantly correlated with SBP.

Table4 Relationship between various characteristics and blood pressure status of participants (N=4513)

Variables	DBP r(*P*)	SBP r(*P*)
Age	-0.016(0.275)	0.259(<0.001)
Educational attainment	-0.016(0.288)	-0.106(<0.001)
Marital status	-0.013(0.388)	-0.139(<0.001)
Current residence	0.012(0.435)	0.023(0.121)
Smoking status	0.006(0.673)	0.029(0.051)
Drinking	-0.045(0.003)	-0.051(0.001)
Eating habits	-0.055(<0.001)	-0.023(0.117)
Entertainment	0.018(0.216)	0.000(0.975)
The experience of a traumatic event	-0.018(0.219)	-0.027(0.075)
Physical exercises	0.002(0.873)	-0.034(0.024)
History of CVDs	0.035(0.018)	0.058(<0.001)
History of liver diseases	-0.035(0.018)	-0.022(0.146)
Antilipidemic medication	0.078(<0.001)	0.078(<0.001)
Antidiabetic medication	0.025(0.100)	0.083(<0.001)
LDL-C	0.050(0.001)	0.060(<0.001)
HDL-C	-0.099(<0.001)	-0.062(<0.001)
LDL-C/HDL-C	0.106(<0.001)	0.094(<0.001)
TG	0.100(<0.001)	0.091(<0.001)
HbA_1c	0.061(<0.001)	0.066(<0.001)
Scr	0.056(<0.001)	0.076(<0.001)

For peer review only - http://bmjopen.bmj.com/site/about/guidelines.xhtml

SUA	0.085(<0.001)	0.111(<0.001)
BMI	0.221(<0.001)	0.129(<0.001)
WC	0.183(<0.001)	0.177(<0.001)
Menarche age	-0.060(<0.001)	-0.014(0.335)

CVDs: cardiovascular diseases; LDL-C: low-density lipoprotein cholesterol; HDL-C: high-density lipoprotein cholesterol; TG: triglycerides; HbA1c: Hemoglobin A1C; Scr: serum creatinine; SUA: serum uric acid; BMI: body mass index; WC: waist circle; DBP: diastolic blood pressure; SBP: systolic blood pressure.

In the moderator analysis, we tested the interaction between menarche age and obesity in predicting systolic and diastolic BP. The standard error, standardized coefficients and the adjusting associated 95% confidence intervals (CIs) are shown in Table 5. We controlled for socio-demographic characteristics, health behaviors, medical histories, and metabolic measures. After controlling for the total potential confounders, the moderating effect of obesity indicators on the relationship between age of menarche and BP was tested. The interaction term of obesity parameters*age of menarche was not significant for predicting either DBP (BMI, adjusted P= 0.2556; WC, adjusted P= 0.1833) or SBP (BMI, adjusted P= 0.8561; WC, adjusted P= 0.8427) in women.

For peer review only - http://bmjopen.bmj.com/site/about/guidelines.xhtml

For peer review only

Table5 Moderator effect of obesity on the relationship between menarche age and BP in women (N=4513)

Dependent	Variables	Model one[a]			Model two[b]			Model three[c]			Model four[d]			Model five[e]		
		SE	B (95%*CI*)	*P*	se	B (95%*CI*)	*P*	SE	B (95%*CI*)	*P*	SE	B (95%*CI*)	*P*	se	B (95%*CI*)	*P*
DBP	Menarche age	0.0835	-0.2027(-0.3665, -0.0390)	0.0153	0.0855	-0.2528(-0.4203, -0.0852)	0.0031	0.0853	-0.2411(-0.4084, -0.0738)	0.0047	0.0854	-0.2485(-0.4158, -0.0811)	0.0036	0.0973	-0.2162(-0.4069, -0.0254)	0.0263
	BMI	0.0440	0.6546(0.5684,0.7409)	<0.001	0.0447	0.6757(0.5881,0.7634)	<0.001	0.0449	0.6835(0.5955,0.7715)	<0.001	0.0458	0.6647(0.5750,0.7544)	<0.001	0.0556	0.5916(0.4827,0.7005)	<0.001
	BMI× Menarche age	0.0204	0.0279(-0.0121,0.0678)	0.1719	0.0204	0.0258(-0.0141,0.0658)	0.2046	0.0203	0.0247(-0.0152,0.0645)	0.2248	0.0203	0.0220(-0.0178,0.0618)	0.2790	0.0229	0.0260(-0.0189,0.071)	0.2556
SBP	Menarche age	0.1826	-0.0003(-0.3583,0.3576)	0.9986	0.1789	-0.5822(-0.9329, -0.2315)	0.0011	0.1788	-0.5682(-0.9187, -0.2177)	0.0015	0.1795	-0.5944(-0.9462, -0.2425)	<0.001	0.2141	-0.5084(-0.9282, -0.0886)	0.0176
	BMI	0.0964	0.8361(0.6470,1.0251)	<0.001	0.0938	1.0927(0.9088,1.2766)	<0.001	0.0942	1.0906(0.9058,1.2753)	<0.001	0.0965	1.0287(0.8396,1.2178)	<0.001	0.1223	0.8846(0.6448,1.1244)	<0.001
	BMI× Menarche age	0.0446	0.0178(-0.0696,0.1052)	0.6896	0.0426	0.0111(-0.0726,0.0947)	0.7955	0.0426	0.0113(-0.0722,0.0948)	0.7914	0.0427	0.0052(-0.0786,0.0889)	0.9038	0.0504	0.0091(-0.0897,0.108)	0.8561
DBP	Menarche age	0.0838	-0.2569(-0.4213, -0.0925)	0.0022	0.0861	-0.2588(-0.4277, -0.0900)	0.0027	0.0861	-0.2481(-0.4168, -0.0794)	0.0040	0.0861	-0.2561(-0.4248, -0.0873)	0.0029	0.0979	-0.2001(-0.3921, -0.0082)	0.0410
	WC	0.0142	0.1732(0.1452,0.2011)	<0.001	0.0143	0.1752(0.1471,0.2032)	<0.001	0.0143	0.1730(0.1449,0.2011)	<0.001	0.0145	0.1645(0.1361,0.193)	<0.001	0.0172	0.1472(0.1135,0.1809)	<0.001
	WC× Menarche age	0.0065	0.0058(-0.007,0.0186)	0.3758	0.0066	0.0054(-0.0075,0.0182)	0.4136	0.0065	0.005(-0.0079,0.0178)	0.4473	0.0065	0.0043(-0.0085,0.0171)	0.5075	0.0074	0.0099(-0.0047,0.0244)	0.1833
SBP	Menarche age	0.1777	-0.0009(-0.3492,0.3475)	0.9961	0.1761	-0.5656(-0.9107, -0.2204)	0.0013	0.1760	-0.5546(-0.8997, -0.2094)	0.0016	0.1766	-0.581(-0.9273, -0.2347)	0.0010	0.2096	-0.4548(-0.8658, -0.0438)	0.0301
	WC	0.0302	0.3637(0.3044,0.4229)	<0.001	0.0293	0.3228(0.2654,0.3802)	<0.001	0.0294	0.3153(0.2578,0.3729)	<0.001	0.0298	0.2944(0.236,0.3528)	<0.001	0.0368	0.2744(0.2022,0.3466)	<0.001
	WC× Menarche age	0.0139	-0.0156(-0.0428,0.0116)	0.2607	0.0134	-0.0061(-0.0323,0.0202)	0.6501	0.0134	-0.0063(-0.0326,0.0199)	0.6367	0.0134	-0.0081(-0.0344,0.0182)	0.5443	0.0159	-0.0032(-0.0343,0.028)	0.8427

CVDs: cardiovascular diseases; LDL-C: low-density lipoprotein cholesterol; HDL-C: high-density lipoprotein cholesterol; TG: triglycerides; HbA1c: Hemoglobin A1C; Scr: serum creatinine; SUA: serum uric acid; BMI: body mass index; WC: waist circle; DBP: diastolic blood pressure; SBP: systolic blood pressure; Un-standardized: B; Standard Error: SE.

[a] **Unadjusted;**

[b] **Adjusted for** age, educational attainment, marital status, region;

[c] **Adjusted for** age, educational attainment, marital status, region, smoking status, alcohol consumption, eating habits, entertainment, experience of a traumatic event, physical activity;

[d] **Adjusted for** age, educational attainment, marital status, region, smoking status, alcohol consumption, eating habits, entertainment, experience of a traumatic event, physical activity, history of CVDs, hepatitis history, antihyperlipidemic drugs, antidiabetic drugs;

[e] **Adjusted for** age, educational attainment, marital status, region, smoking status, alcohol consumption, eating habits, entertainment, experience of a traumatic event, physical activity, history of CVDs, hepatitis history, antihyperlipidemic drugs, antidiabetic drugs, LDL-C, HDL-C, TG, HbA1c, Scr, SUA.

For peer review only - http://bmjopen.bmj.com/site/about/guidelines.xhtml

For peer review only

In the mediation analysis, we estimated the BP equation using bootstrap inference for model coefficients. The un-standardized, standardized coefficients and the adjusting associated 95% confidence intervals (CIs) are shown in Table 6. We controlled for socio-demographic characteristics, health behaviors, medical histories, and metabolic measures. After controlling for the total potential confounders, the mediating effect of obesity indicators on the relationship between age of menarche and BP was tested. All correlations were significant correlation between age of menarche, obesity parameters and BP except the path of the age of menarche→SBP (with the addition of the BMI or WC indicator) in model one. In general, after controlling for potential confounders. WC and BMI partly mediated the relationship between the age of menarche and BP.

For peer review only

For peer review only - http://bmjopen.bmj.com/site/about/guidelines.xhtml

Table6 Mediation effect of obesity on the relationship between age at menarche and BP in women (N=4513)

Path		Model one[a]			Model two[b]			Model three[c]			Model four[d]			Model five[e]		
		β	Β (95%*CI*)	*P*	β	Β (95%*CI*)	*P*	β	Β (95%*CI*)	*P*	β	Β (95%*CI*)	*P*	β	Β (95%*CI*)	*P*
**Path1**	Menarche age→BMI	-0.1080	-0.2045(-0.2595, -0.1496)	<0.001	-0.0725	-0.1374(-0.1930, -0.0818)	<0.001	-0.0707	-0.1339(-0.1892, -0.0786)	<0.001	-0.0705	-0.1334(-0.1880, -0.0788)	<0.001	-0.0468	-0.0885(-0.1409, -0.0361)	<0.001
	Menarche age→DBP	-0.0367	-0.2096(-0.3731, -0.0461)	0.0120	-0.0454	-0.2596(-0.4268, -0.0924)	0.0024	-0.0433	-0.2475(-0.4145, -0.0806)	0.0037	-0.0447	-0.2542(-0.4213, -0.0872)	0.0029	-0.0379	-0.2154(-0.3833, -0.0474)	0.0120
	BMI→DBP	0.2171	0.6545(0.5682,0.7408)	<0.001	0.2241	0.6757(0.5881, 0.7634)	<0.001	0.2276	0.6835(0.5955, 0.7715)	<0.001	0.2208	0.6643(0.5746,0.7541)	<0.001	0.1986	0.5973(0.5026,0.6921)	<0.001
	Total effect	-0.0601	-0.3435(-0.5099, -0.177)	<0.001	-0.0617	-0.3524(-0.5234, -0.1815)	<0.001	-0.0593	-0.3390(-0.5098, -0.1683)	<0.001	-0.0602	-0.3429(-0.5133, -0.1724)	<0.001	-0.0471	-0.2682(-0.4388, -0.0976)	0.0021
**Path2**	Menarche age→BMI	0.1085	-0.2051(-0.2601, -0.1502)	<0.001	-0.0731	-0.1381(-0.1936, -0.0826)	<0.001	-0.0712	-0.1346(-0.1898, -0.0793)	<0.001	-0.0711	-0.1342(-0.1887, -0.0797)	<0.001	-0.0473	-0.0892(-0.1416, -0.0369)	<0.001
	Menarche age→SBP	-0.0004	-0.0046(-0.3619,0.3526)	0.9797	-0.0477	-0.585(-0.9350, -0.2351)	0.0011	-0.0466	-0.5711(-0.9209, -0.2213)	0.0014	-0.0486	-0.5957(-0.9468, -0.2446)	<0.001	-0.0446	-0.5479(-0.9025, -0.1932)	0.0025
	BMI→SBP	0.1289	0.8359(0.6469,1.0249)	<0.001	0.1685	1.0927(0.9088, 1.2766)	<0.001	0.1682	1.0905(0.9058,1.2753)	<0.001	0.1585	1.0286(0.8395,1.2176)	<0.001	0.1441	0.9366(0.7361,1.1371)	<0.001
	Total effect	-0.0144	-0.1761(-0.5342,0.182)	0.3350	-0.0601	-0.736(-1.0902, -0.3817)	<0.001	-0.0586	-0.7178(-1.0719, -0.3638)	<0.001	-0.0599	-0.7337(-1.0883, -0.3791)	<0.001	-0.0515	-0.6314(-0.9889, -0.2739)	<0.001
**Path3**	Menarche age→WC	-0.0837	-0.4928(-0.6642, -0.3213)	<0.001	-0.0910	-0.5355(-0.7107, -0.3602)	<0.001	-0.0896	-0.5275(-0.7024, -0.3526)	<0.001	-0.0898	-0.5285(-0.7026, -0.3545)	<0.001	-0.0696	-0.4085(-0.5778, -0.2392)	<0.001
	Menarche age→DBP	-0.0454	-0.2589(-0.4232, -0.0945)	0.0020	-0.0456	-0.26(-0.4288, -0.0912)	0.0025	-0.0437	-0.2491(-0.4178, -0.0805)	0.0038	-0.0452	-0.2571(-0.4257, -0.0884)	0.0028	-0.0373	-0.2118(-0.3811, -0.0424)	0.0143
	WC→DBP	0.1789	0.1733(0.1454, 0.2013)	<0.001	-0.1810	0.1754(0.1473, 0.2034)	<0.001	0.1787	0.1732(0.1451, 0.2013)	<0.001	0.1705	0.1647(0.1363, 0.1931)	<0.001	0.1445	0.1399(0.1104, 0.1695)	<0.001
	Total effect	-0.0604	-0.3443(-0.5107, -0.1779)	<0.001	-0.0620	-0.3539(-0.5248, -0.183)	<0.001	-0.0597	-0.3405(-0.5112, -0.1698)	<0.001	-0.0606	-0.3441(-0.5145, -0.1737)	<0.001	-0.0474	-0.2689(-0.4395, -0.0984)	0.0020
**Path4**	Menarche age→WC	-0.0849	-0.4944(-0.6658, -0.323)	<0.001	-0.0914	-0.5376(-0.7129, -0.3624)	<0.001	-0.0901	-0.5299(-0.7048, -0.355)	<0.001	-0.0904	-0.531(-0.7051, -0.357)	<0.001	-0.0702	-0.411(-0.5803, -0.2417)	<0.001
	Menarche age→SBP	0.0004	0.0044(-0.3439, 0.3526)	0.9804	-0.0468	-0.5643(-0.9094, -0.2192)	0.0014	-0.0459	-0.5533(-0.8983, -0.2082)	0.0017	-0.0481	-0.5792(-0.9255, -0.233)	0.0010	-0.0433	-0.5228(-0.8721, -0.1735)	0.0034
	WC→SBP	0.1772	0.3632(0.3040, 0.4224)	<0.001	0.1574	0.3226(0.2652,0.38)	<0.001	0.1538	0.3151(0.2576, 0.3727)	<0.001	0.1436	0.2942(0.2358, 0.3526)	<0.001	0.1290	0.2655(0.2045,0.3266)	<0.001
	Total effect	-0.0145	-0.1752(-0.5277, 0.1773)	0.3300	-0.0612	-0.7377(-1.086, -0.3894)	<0.001	-0.0598	-0.7202(-1.0683, -0.3722)	<0.001	-0.0611	-0.7354(-1.084, -0.3869)	<0.001	-0.0524	-0.632(-0.9832, -0.2807)	<0.001

CVDs: cardiovascular diseases; LDL-C: low-density lipoprotein cholesterol; HDL-C: high-density lipoprotein cholesterol; TG: triglycerides; HbA1c: Hemoglobin A1C; Scr: serum creatinine; SUA: serum uric acid; BMI: body mass index; WC: waist circle; DBP: diastolic blood pressure; SBP: systolic blood pressure; Un-standardized: B, standardized coefficients, β.

Path1: Mediation effect of BMI on the relationship between age at menarche and DBP.

Path2: Mediation effect of BMI on the relationship between age at menarche and SBP.

Path3: Mediation effect of WC on the relationship between age at menarche and DBP.

Path4: Mediation effect of WC on the relationship between age at menarche and SBP.

[a] **Unadjusted;**

[b] **Adjusted for** age, educational attainment, marital status, region;

[c] **Adjusted for** age, educational attainment, marital status, region, cigarette smoking, alcohol consumption, eating habits, entertainment, the experience of a traumatic event, physical activity;

[d] **Adjusted for** age, educational attainment, marital status, region, cigarette smoking, alcohol consumption, eating habits, entertainment, the experience of a traumatic event, physical activity, history of CVDs, hepatitis history, history of antilipidemic medication, history of antidiabetic medication;

[e] **Adjusted for** age, educational attainment, marital status, region, cigarette smoking, alcohol consumption, eating habits, entertainment, the experience of a traumatic event, physical activity, history of CVDs, hepatitis history, history of antilipidemic medication, history of antidiabetic medication, LDL-C, HDL-C, TG, HbA1c, Scr, SUA.

For peer review only

For peer review only - http://bmjopen.bmj.com/site/about/guidelines.xhtml

DISCUSSION

In the study, we attempted to explore the association between age of menarche and BP and examined the moderating and mediating effect of obesity parameters (BMI and WC) on the association. We have found that the interaction term of obesity parameters*age of menarche was not significant for predicting BP. It was also important to note that WC and BMI partly mediated the association between age of menarche and BP. The results of our study were partly constant with most previous studies. And so previous studier had only focus on the relationship between earlier biological maturation and health outcomes in late adolescence and adulthood. Most of the previous studies meant exactly that: earlier biological maturation was at a higher risk for health outcomes in late adolescence and adulthood. Furthermore, most previous studies found that early biological maturation (age of menarche) or puberty was significantly related to obesity parameters[33-37], hypertension[37-40], and metabolic syndrome[41-49]. In general, our results were mostly consistent with the results of previous studies that suggest that earlier biological maturation is a risk factor for several negative health outcomes in late adolescence and adulthood.

Our study suggested the likelihood of a dose-response relationship between age of menarche and obesity parameters (BMI and WC). The relationship could be identified by the fact that early-maturing girls already have more adiposity before menarche, given that fatty tissue, through leptin release, has a crucial role in the initiation of the biological maturation process[50 51]. Early maturing girls could maintain their higher fatty tissue in adulthood[52]. Another mechanism may also be associated with the time of the maturation process. It has been reported that early maturing girls could experience a more accelerated maturation process than late maturing ones. Thus, more significant interference with biological maturation in girls can lead to increased more fatty tissue[53]. We also found a significantly negative relationship between age at menarche and BP in mid-aged and elderly Chinese women. However, this association did not exist between the age of menarche and SBP before adjusting the related confounders. This result is consistent with previous findings[54-62] regarding the age of menarche as a cardiovascular risk factor. The weak association between age at menarche and BP in adulthood could, in part, be explained by the fact that early matures were younger than women with late menarche. This result could be an expression of the fact that BP is an outcome with a higher latency period than obesity. Moreover, among women who already entered menopause in our study, the effects of menarche could be even weaker, as results suggest, and obesity could have more effect on BP. We also found that women with a higher age of menarche had higher HDL-C

For peer review only - http://bmjopen.bmj.com/site/about/guidelines.xhtml

level but had lower DBP level. The results of the current study were quite consistent with those of [55] Feng Y, *et al.* Increased body fat, especially abdominal fat, partially explained the increased insulin resistance and dyslipidemia in women with early menarche age. Therefore, obesity in adolescence, sociodemographic, or even behavioral factors through life (including age, educational attainment, marital status, region, smoking status, alcohol consumption, eating habits, entertainment, the experience of a traumatic event, physical activity) were potential mediators to target when interventions for BP control and management.

Though so many studies have explored the association analysis between the age of menarche or obesity parameters and BP, there were only two studies[63 64] that explored the mediating effect of obesity parameters on the relation between age of menarche and BP. Zhang L, *et al.* [63] found that the relationship between age of menarche and DBP was partly mediated by WC. In contrast, the relationship between age of menarche and SBP was fully mediated by WC in women. Werneck AO, *et al.* [64] showed that women with late menarche are less likely to have a risk of hypertension, and BMI was an important mediator of the age at the menarche-hypertension association. Interestingly, we found that obesity parameters partly mediated the relationship between the age of menarche and BP in women. The difference between our research and others may due to the different populations, different definitions of early and later menarche, and different confounding variables by controlling. The individuals in our study were mid-aged and elderly Chinese women, where the mean age at recruitment and age of menarche were older than in the Werneck AO, *et al*' s study[64], and the level of socio-economic development also made some contribution to ontogenetic development. In addition, other studies and a meta-analysis[54 56 62 65-67] reported an inverse association between early age of menarche and hypertension, which was not observed in Zhang L, *et al*' study[63]. They found no direct relationship between the age of menarche and DBP, and we have found a direct relationship between the age of menarche and DBP. The participants were similar in China and its socio-economic background, this phenomenon could be explained by the cumulative effect, which showed a significantly negative association between age of menarche and DBP over life-span.

There were several limitations to the study. The main limitations of our research are related to the cross-sectional study and the self-reported method used for the assessment of age at menarche and most related confounders. BP in our study was measured at home by professionally trained volunteers, and the next step is the use of clinical BP and ambulatory blood pressure. However, this is the first

For peer review only - http://bmjopen.bmj.com/site/about/guidelines.xhtml

large population study to examine the moderation between the age of menarche and obesity parameters in predicting BP in middle-aged and elderly Chinese, as well as the mediation effects of obesity parameters on the relationship between age of menarche and BP. Moreover, a significant strength of the study is a large sample of 4513 middle-aged and older Chinese. The results presented in this article represent the baseline data that could be explored further in prospective cohort studies.

In sum, the interaction term of obesity parameters*age of menarche was not significant for predicting either DBP or SBP in women. Moreover, obesity parameters partly mediated the relationship between the age of menarche and BP.

CONCLUSIONS

The interaction term of obesity parameters*age of menarche was not significant for predicting either DBP or SBP in women. Moreover, Obesity parameters partly mediated the relationship between age of menarche and BP.

Author affiliations

[1] Department of Internal Medicine Nursing, School of Nursing, Wannan Medical College, 22 Wenchang West Road, Higher Education Park, Wuhu City, An Hui Province, P.R.China

[2] Obstetrics and Gynecology Nursing, School of Nursing, Wannan Medical College, 22 Wenchang West Road, Higher Education Park, Wuhu City, An Hui Province, P.R.China

[3]Department of Pediatric Nursing, School of Nursing, Wannan Medical College, 22 Wenchang West Road, Higher Education Park, Wuhu City, An Hui Province, P.R.China

[4] Department of Surgical Nursing, School of Nursing, Wannan Medical College, 22 Wenchang West Road, Higher Education Park, Wuhu City, An Hui Province, P.R.China

[5]Department of Emergency and Critical Care Nursing, School of Nursing, Wannan Medical College, 22 Wenchang West Road, Higher Education Park, Wuhu City, An Hui Province, P.R.China

[6]Rehabilitation Nursing, School of Nursing, Wanna Medical college, 22 Wenchang West Road, Higher Education Park, Wuhu City, An Hui Province, P.R.China

[7]Department of nursing, Yijishan Hospital, the First Affiliated Hospital of Wannan Medical College, Zheshan West Road, Yijishan District, Wuhu City, Anhui Province

Abbreviations

BMI, body mass index; BP, blood pressure; Confidence intervals, CIs; CDC, Centers for Disease Control and Prevention; CHARLS, China Health and Retirement Longitudinal Study; CVDs,

For peer review only

For peer review only - http://bmjopen.bmj.com/site/about/guidelines.xhtml

cardiovascular diseases; DBP, diastolic blood pressure; HDL, high-density lipoprotein; Hemoglobin A1C, HbA1c; LDL, low-density lipoprotein; M, mean; NSFC, The National Natural Science Foundation of China; National Institute on Aging, NIA; SBP, systolic blood pressure; SUA, serum uric acid; standard error, SE; unstandardized, B; standardized coefficients, β; Scr, serum creatinine; SD, standard deviation; TG, triglycerides; urinalysis, UA; World Bank, WB; waist circle, WC; World Health Organization, WHO.

Acknowledgements

We are grateful to the China Center for Economic Research, and we also thank all participants for their contribution and members of the research for collecting the data.

Author contributions

Conceived and designed the research: LZ. Wrote the paper: LZ. Analyzed the data: LZ. Revised the paper: LZ, L Y, C-z W, TY, Dm Z, H-h W, JL, Y-x L, LS, X-p L, YH, H-y C, and Y-z L.

Funding

CHARLS was supported by the NSFC (70910107022, 71130002) and National Institute on Aging (R03-TW008358-01; R01-AG037031-03S1), and World Bank (7159234) and the publication fee was supported by the Support Program for Outstanding Young Talents from the Universities and Colleges of Anhui Province for Lin Zhang(gxyqZD2021118).

Disclosure

The authors declare that there is no potential conflict of interest relevant to the research.

Patient consent

Completion of all authors declaration and consent to publish form is required.

Ethics approval and consent to participate

Ethics approval for the research was granted by the Ethics Review Committee of Peking University, and all participants provided informed consent. Approval for this study was given by the medical ethics committee of Wannan medical college (approval number 2021–3).

Data sharing

Data sharing statement Extra data can be accessed via http://charls.pku.edu.cn/pages/data/111/zh-cn.html.

For peer review only - http://bmjopen.bmj.com/site/about/guidelines.xhtml

For peer review only

References

1. Tian S, Xu Y, Dong H. The effect of metabolic health and obesity phenotypes on risk of hypertension: A nationwide population-based study using 5 representative definitions of metabolic health. Medicine (Baltimore) 2018;**97**(38):e12425 doi: 10.1097/MD.0000000000012425[published Online First: Epub Date]|.
2. Abdulsalam S, Olugbenga-Bello A, Olarewaju O, et al. Sociodemographic correlates of modifiable risk factors for hypertension in a rural local government area of oyo state South west Nigeria. Int J Hypertens 2014;**2014**:842028 doi: 10.1155/2014/842028[published Online First: Epub Date]|.
3. Ke L, Ho J, Feng J, et al. Modifiable risk factors including sunlight exposure and fish consumption are associated with risk of hypertension in a large representative population from Macau. J Steroid Biochem Mol Biol 2014;**144 Pt A**:152-5 doi: 10.1016/j.jsbmb.2013.10.019[published Online First: Epub Date]|.
4. Ibekwe R. Modifiable Risk factors of Hypertension and Socio-demographic Profile in Oghara, Delta State; Prevalence and Correlates. Ann Med Health Sci Res 2015;**5**(1):71-7 doi: 10.4103/2141-9248.149793[published Online First: Epub Date]|.
5. Pilakkadavath Z, Shaffi M. Modifiable risk factors of hypertension: A hospital-based case-control study from Kerala, India. J Family Med Prim Care 2016;**5**(1):114-9 doi: 10.4103/2249-4863.184634[published Online First: Epub Date]|.
6. Mamudu HM, Paul TK, Wang L, et al. Association Between Multiple Modifiable Risk Factors of Cardiovascular Disease and Hypertension among Asymptomatic Patients in Central Appalachia. South Med J 2017;**110**(2):90-96 doi: 10.14423/SMJ.0000000000000602[published Online First: Epub Date]|.
7. Collaboration NCDRF. Worldwide trends in blood pressure from 1975 to 2015: a pooled analysis of 1479 population-based measurement studies with 19.1 million participants. Lancet 2017;**389**(10064):37-55 doi: 10.1016/S0140-6736(16)31919-5[published Online First: Epub Date]|.
8. Bromfield S, Muntner P. High blood pressure: the leading global burden of disease risk factor and the need for worldwide prevention programs. Curr Hypertens Rep 2013;**15**(3):134-6 doi: 10.1007/s11906-013-0340-9[published Online First: Epub Date]|.
9. Mahajan S, Zhang D, He S, et al. Prevalence, Awareness, and Treatment of Isolated Diastolic Hypertension: Insights From the China PEACE Million Persons Project. J Am Heart Assoc 2019;**8**(19):e012954 doi: 10.1161/JAHA.119.012954[published Online First: Epub Date]|.
10. Cloutier L, Morris D, Bruneau J, et al. World Health Organization celebrates World Health Day, April 7,2013--focusing on hypertension. Canadian journal of cardiovascular nursing = Journal canadien en soins infirmiers cardio-vasculaires 2013;**23**(2):9-11
11. Webb MS. Stress management strategies in hypertension control. Ethnicity & disease 2002;**12**(4):S3-95-100
12. Kotchen TA. The search for strategies to control hypertension. Circulation 2010;**122**(12):1141-3 doi: 10.1161/CIRCULATIONAHA.110.978759[published Online First: Epub Date]|.
13. Mancia G, Rea F, Cuspidi C, et al. Blood pressure control in hypertension. Pros and cons of available treatment strategies. Journal of hypertension 2017;**35**(2):225-33 doi: 10.1097/HJH.0000000000001181[published Online First: Epub Date]|.
14. Werneck AO, Silva DR, Souza MF, et al. Correlates of Blood Pressure According to Early, On Time, and Late Maturation in Adolescents. Journal of clinical hypertension 2016;**18**(5):424-30 doi: 10.1111/jch.12699[published Online First: Epub Date]|.
15. Widen E, Silventoinen K, Sovio U, et al. Pubertal timing and growth influences cardiometabolic risk factors in adult males and females. Diabetes care 2012;**35**(4):850-6 doi: 10.2337/dc11-1365[published Online First: Epub Date]|.
16. Mueller NT, Duncan BB, Barreto SM, et al. Earlier age at menarche is associated with higher diabetes risk and

For peer review only

cardiometabolic disease risk factors in Brazilian adults: Brazilian Longitudinal Study of Adult Health (ELSA-Brasil). Cardiovasc Diabetol 2014;**13**:22 doi: 10.1186/1475-2840-13-22[published Online First: Epub Date]|.

17. Dreyfus J, Jacobs DR, Jr., Mueller N, et al. Age at Menarche and Cardiometabolic Risk in Adulthood: The Coronary Artery Risk Development in Young Adults Study. J Pediatr 2015;**167**(2):344-52 e1 doi: 10.1016/j.jpeds.2015.04.032[published Online First: Epub Date]|.
18. Cao M, Cui B. Negative Effects of Age at Menarche on Risk of Cardiometabolic Diseases in Adulthood: A Mendelian Randomization Study. The Journal of clinical endocrinology and metabolism 2020;**105**(2) doi: 10.1210/clinem/dgz071[published Online First: Epub Date]|.
19. Power C, Lake JK, Cole TJ. Body mass index and height from childhood to adulthood in the 1958 British born cohort. The American journal of clinical nutrition 1997;**66**(5):1094-101 doi: 10.1093/ajcn/66.5.1094[published Online First: Epub Date]|.
20. Adair LS, Gordon-Larsen P. Maturational timing and overweight prevalence in US adolescent girls. American journal of public health 2001;**91**(4):642-4 doi: 10.2105/ajph.91.4.642[published Online First: Epub Date]|.
21. Must A, Naumova EN, Phillips SM, et al. Childhood overweight and maturational timing in the development of adult overweight and fatness: the Newton Girls Study and its follow-up. Pediatrics 2005;**116**(3):620-7 doi: 10.1542/peds.2004-1604[published Online First: Epub Date]|.
22. Li C, Liu T, Sun W, et al. Prevalence and risk factors of arthritis in a middle-aged and older Chinese population: the China health and retirement longitudinal study. Rheumatology 2015;**54**(4):697-706 doi: 10.1093/rheumatology/keu391[published Online First: Epub Date]|.
23. Zhang L, Yang L, Wang C, et al. Individual and combined association analysis of famine exposure and serum uric acid with hypertension in the mid-aged and older adult: a population-based cross-sectional study. BioMed research international 2021;**21**(1):420 doi: 10.1155/2021/5594718
10.1186/s12872-021-02230-z[published Online First: Epub Date]|.
24. Zhang L, Yang L, Wang C, et al. Combined Effect of Famine Exposure and Obesity Parameters on Hypertension in the Midaged and Older Adult: A Population-Based Cross-Sectional Study. 2021;**2021**:5594718 doi: 10.1155/2021/5594718[published Online First: Epub Date]|.
25. Zhang L, Li JL, Zhang LL, et al. Body mass index and serum uric acid level: Individual and combined effects on blood pressure in middle-aged and older individuals in China. Medicine (Baltimore) 2020;**99**(9):e19418 doi: 10.1097/MD.0000000000019418[published Online First: Epub Date]|.
26. Zhang L, Li JL, Zhang LL, et al. Relationship between adiposity parameters and cognition: the "fat and jolly" hypothesis in middle-aged and elderly people in China. Medicine 2019;**98**(10) doi: ARTN e14747
10.1097/MD.0000000000014747[published Online First: Epub Date]|.
27. Zhang L, Li JL, Zhang LL, et al. Association and Interaction Analysis of Body Mass Index and Triglycerides Level with Blood Pressure in Elderly Individuals in China. BioMed research international 2018;**2018**:8934534 doi: 10.1155/2018/8934534[published Online First: Epub Date]|.
28. Zhang L, Li JL, Zhang LL, et al. No association between C-reactive protein and depressive symptoms among the middle-aged and elderly in China Evidence from the China Health and Retirement Longitudinal Study. Medicine 2018;**97**(38) doi: ARTN e12352
10.1097/MD.0000000000012352[published Online First: Epub Date]|.
29. Zhang L, Liu K, Li H, et al. Relationship between body mass index and depressive symptoms: the "fat and jolly" hypothesis for the middle-aged and elderly in China. BMC public health 2016;**16**(1):1201 doi: 10.1186/s12889-016-3864-5[published Online First: Epub Date]|.
30. Hayes AF, Preacher KJ. Quantifying and Testing Indirect Effects in Simple Mediation Models When the Constituent Paths Are Nonlinear. Multivariate Behav Res 2010;**45**(4):627-60 doi: 10.1080/00273171.2010.498290[published Online First: Epub Date]|.
31. Hayes AF, Preacher KJ. Statistical mediation analysis with a multicategorical independent variable. Br J Math Stat

For peer review only - http://bmjopen.bmj.com/site/about/guidelines.xhtml

Psychol 2014;**67**(3):451-70 doi: 10.1111/bmsp.12028[published Online First: Epub Date]|.
32. Hayes AF. An Index and Test of Linear Moderated Mediation. Multivariate Behav Res 2015;**50**(1):1-22 doi: 10.1080/00273171.2014.962683[published Online First: Epub Date]|.
33. Shrestha A, Olsen J, Ramlau-Hansen CH, et al. Obesity and age at menarche. Fertil Steril 2011;**95**(8):2732-4 doi: 10.1016/j.fertnstert.2011.02.020[published Online First: Epub Date]|.
34. Bralic I, Tahirovic H, Matanic D, et al. Association of early menarche age and overweight/obesity. J Pediatr Endocrinol Metab 2012;**25**(1-2):57-62 doi: 10.1515/jpem-2011-0277[published Online First: Epub Date]|.
35. Leitao RB, Rodrigues LP, Neves L, et al. Development of adiposity, obesity and age at menarche: an 8-year follow-up study in Portuguese schoolgirls. International journal of adolescent medicine and health 2013;**25**(1):55-63 doi: 10.1515/ijamh-2013-0007[published Online First: Epub Date]|.
36. Gallicchio L, Flaws JA, Smith RL. Age at menarche, androgen concentrations, and midlife obesity: findings from the Midlife Women's Health Study. Menopause 2016;**23**(11):1182-88 doi: 10.1097/GME.0000000000000691[published Online First: Epub Date]|.
37. Liu G, Yang Y, Huang W, et al. Association of age at menarche with obesity and hypertension among southwestern Chinese women: a new finding. Menopause 2018;**25**(5):546-53 doi: 10.1097/GME.0000000000001027[published Online First: Epub Date]|.
38. Zhou W, Wang T, Zhu L, et al. Association between Age at Menarche and Hypertension among Females in Southern China: A Cross-Sectional Study. Int J Hypertens 2019;**2019**:9473182 doi: 10.1155/2019/9473182[published Online First: Epub Date]|.
39. Shen L, Wang L, Hu Y, et al. Associations of the ages at menarche and menopause with blood pressure and hypertension among middle-aged and older Chinese women: a cross-sectional analysis of the baseline data of the China Health and Retirement Longitudinal Study. Hypertension research : official journal of the Japanese Society of Hypertension 2019;**42**(5):730-38 doi: 10.1038/s41440-019-0235-5[published Online First: Epub Date]|.
40. Guo L, Peng C, Xu H, et al. Age at menarche and prevention of hypertension through lifestyle in young Chinese adult women: result from project ELEFANT. BMC Womens Health 2018;**18**(1):182 doi: 10.1186/s12905-018-0677-y[published Online First: Epub Date]|.
41. Stockl D, Meisinger C, Peters A, et al. Age at menarche and its association with the metabolic syndrome and its components: results from the KORA F4 study. PloS one 2011;**6**(10):e26076 doi: 10.1371/journal.pone.0026076[published Online First: Epub Date]|.
42. Chang CJ, Lai MM, Lin CC, et al. Age at menarche and its association with the metabolic syndrome in Taiwan. Obesity research & clinical practice 2016;**10 Suppl 1**:S26-S34 doi: 10.1016/j.orcp.2015.10.003[published Online First: Epub Date]|.
43. Heys M, Schooling CM, Jiang C, et al. Age of menarche and the metabolic syndrome in China. Epidemiology 2007;**18**(6):740-6 doi: 10.1097/EDE.0b013e3181567faf[published Online First: Epub Date]|.
44. Yoo S, Hwang YS. Age-Group Related Cohort Effects on the Association between Age at Menarche and Metabolic Syndrome among Korean Premenopausal Women. Korean journal of family medicine 2019;**40**(4):280-81 doi: 10.4082/kjfm.19.0045[published Online First: Epub Date]|.
45. Akter S, Jesmin S, Islam M, et al. Association of age at menarche with metabolic syndrome and its components in rural Bangladeshi women. Nutr Metab (Lond) 2012;**9**(1):99 doi: 10.1186/1743-7075-9-99[published Online First: Epub Date]|.
46. Glueck CJ, Morrison JA, Wang P, et al. Early and late menarche are associated with oligomenorrhea and predict metabolic syndrome 26 years later. Metabolism: clinical and experimental 2013;**62**(11):1597-606 doi: 10.1016/j.metabol.2013.07.005[published Online First: Epub Date]|.
47. Kim Y, Je Y. Early Menarche and Risk of Metabolic Syndrome: A Systematic Review and Meta-Analysis. Journal of women's health 2019;**28**(1):77-86 doi: 10.1089/jwh.2018.6998[published Online First: Epub Date]|.

For peer review only - http://bmjopen.bmj.com/site/about/guidelines.xhtml

48. Farahmand M, Ramezani Tehrani F, Behboudi Gandevani S, et al. Is There any Association between Age at Menarche and Risk of Metabolic Syndrome? The Tehran Lipid & Glucose Study. Arch Iran Med 2019;**22**(9):495-500
49. Hwang YS, Park EJ, Choi JG, et al. Relationship between Age at Menarche and Metabolic Syndrome in Premenopausal Women: Korea National Health and Nutrition Examination Survey 2013-2014. Korean journal of family medicine 2018;**39**(5):300-06 doi: 10.4082/kjfm.17.0022[published Online First: Epub Date]|.
50. Ahmed ML, Ong KK, Dunger DB. Childhood obesity and the timing of puberty. Trends Endocrinol Metab 2009;**20**(5):237-42 doi: 10.1016/j.tem.2009.02.004[published Online First: Epub Date]|.
51. Jasik CB, Lustig RH. Adolescent obesity and puberty: the "perfect storm". Ann N Y Acad Sci 2008;**1135**:265-79 doi: 10.1196/annals.1429.009[published Online First: Epub Date]|.
52. Garn SM. Continuities and changes in fatness from infancy through adulthood. Curr Probl Pediatr 1985;**15**(2):1-47 doi: 10.1016/0045-9380(85)90015-5[published Online First: Epub Date]|.
53. van Lenthe FJ, Kemper CG, van Mechelen W. Rapid maturation in adolescence results in greater obesity in adulthood: the Amsterdam Growth and Health Study. The American journal of clinical nutrition 1996;**64**(1):18-24 doi: 10.1093/ajcn/64.1.18[published Online First: Epub Date]|.
54. Remsberg KE, Demerath EW, Schubert CM, et al. Early menarche and the development of cardiovascular disease risk factors in adolescent girls: the Fels Longitudinal Study. The Journal of clinical endocrinology and metabolism 2005;**90**(5):2718-24 doi: 10.1210/jc.2004-1991[published Online First: Epub Date]|.
55. Feng Y, Hong X, Wilker E, et al. Effects of age at menarche, reproductive years, and menopause on metabolic risk factors for cardiovascular diseases. Atherosclerosis 2008;**196**(2):590-7 doi: 10.1016/j.atherosclerosis.2007.06.016[published Online First: Epub Date]|.
56. Won JC, Hong JW, Noh JH, et al. Association Between Age at Menarche and Risk Factors for Cardiovascular Diseases in Korean Women: The 2010 to 2013 Korea National Health and Nutrition Examination Survey. Medicine (Baltimore) 2016;**95**(18):e3580 doi: 10.1097/MD.0000000000003580[published Online First: Epub Date]|.
57. Au Yeung SL, Jiang C, Cheng KK, et al. Age at menarche and cardiovascular risk factors using Mendelian randomization in the Guangzhou Biobank Cohort Study. Preventive medicine 2017;**101**:142-48 doi: 10.1016/j.ypmed.2017.06.006[published Online First: Epub Date]|.
58. Ley SH, Li Y, Tobias DK, et al. Duration of Reproductive Life Span, Age at Menarche, and Age at Menopause Are Associated With Risk of Cardiovascular Disease in Women. J Am Heart Assoc 2017;**6**(11) doi: 10.1161/JAHA.117.006713[published Online First: Epub Date]|.
59. Luijken J, van der Schouw YT, Mensink D, et al. Association between age at menarche and cardiovascular disease: A systematic review on risk and potential mechanisms. Maturitas 2017;**104**:96-116 doi: 10.1016/j.maturitas.2017.07.009[published Online First: Epub Date]|.
60. Yang L, Li L, Millwood IY, et al. Age at menarche and risk of major cardiovascular diseases: Evidence of birth cohort effects from a prospective study of 300,000 Chinese women. International journal of cardiology 2017;**227**:497-502 doi: 10.1016/j.ijcard.2016.10.115[published Online First: Epub Date]|.
61. Chen X, Liu Y, Sun X, et al. Age at menarche and risk of all-cause and cardiovascular mortality: a systematic review and dose-response meta-analysis. Menopause 2018;**26**(6):670-76 doi: 10.1097/GME.0000000000001289[published Online First: Epub Date]|.
62. Lee JJ, Cook-Wiens G, Johnson BD, et al. Age at Menarche and Risk of Cardiovascular Disease Outcomes: Findings From the National Heart Lung and Blood Institute-Sponsored Women's Ischemia Syndrome Evaluation. J Am Heart Assoc 2019;**8**(12):e012406 doi: 10.1161/JAHA.119.012406[published Online First: Epub Date]|.
63. Zhang L, Li Y, Zhou W, et al. Mediation effect of BMI on the relationship between age at menarche and hypertension: The Henan Rural Cohort Study. Journal of human hypertension 2019 doi: 10.1038/s41371-019-0247-2[published Online First: Epub Date]|.
64. Werneck AO, Oyeyemi AL, Cyrino ES, et al. Association between age at menarche and blood pressure in adulthood: is obesity an important mediator? Hypertension research : official journal of the Japanese Society of

For peer review only

For peer review only - http://bmjopen.bmj.com/site/about/guidelines.xhtml

Hypertension 2018;**41**(10):856-64 doi: 10.1038/s41440-018-0079-4[published Online First: Epub Date]|.
65. Lakshman R, Forouhi NG, Sharp SJ, et al. Early age at menarche associated with cardiovascular disease and mortality. The Journal of clinical endocrinology and metabolism 2009;**94**(12):4953-60 doi: 10.1210/jc.2009-1789[published Online First: Epub Date]|.
66. Charalampopoulos D, McLoughlin A, Elks CE, et al. Age at menarche and risks of all-cause and cardiovascular death: a systematic review and meta-analysis. American journal of epidemiology 2014;**180**(1):29-40 doi: 10.1093/aje/kwu113[published Online First: Epub Date]|.
67. Zheng Y, Zhang G, Chen Z, et al. Association between Age at Menarche and Cardiovascular Disease Risk Factors in China: A Large Population-Based Investigation. Cardiorenal Med 2016;**6**(4):307-16 doi: 10.1159/000445506[published Online First: Epub Date]|.

For peer review only

For peer review only - http://bmjopen.bmj.com/site/about/guidelines.xhtml

Fig. 1 Selection of participants

For peer review only

For peer review only - http://bmjopen.bmj.com/site/about/guidelines.xhtml

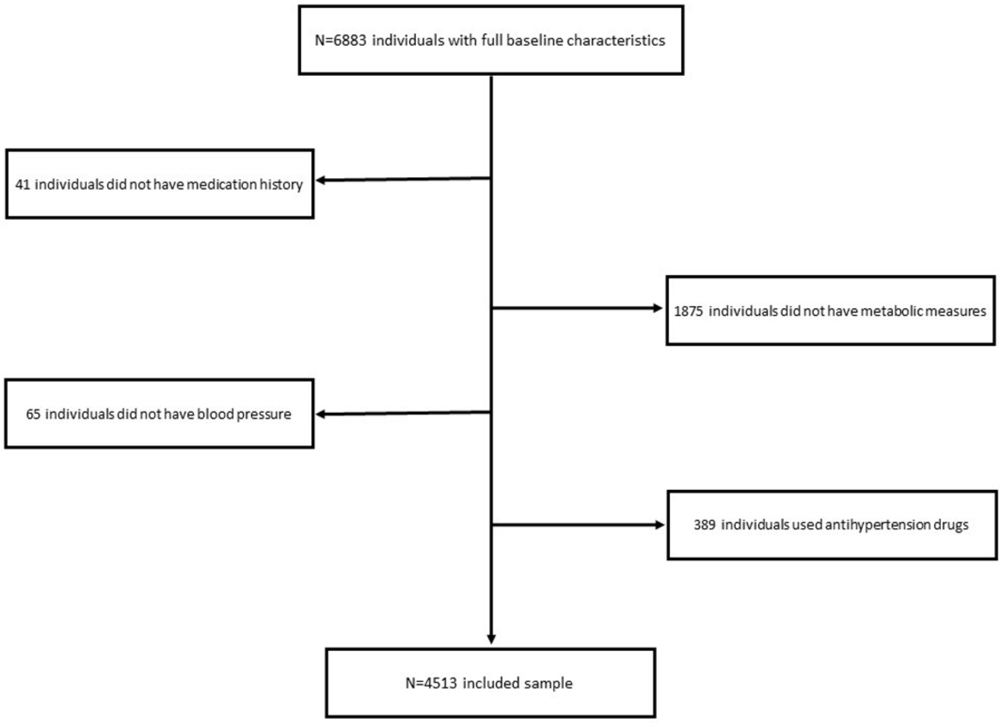

90x90mm (300 x 300 DPI)

STROBE 2007 (v4) Statement—Checklist of items that should be included in reports of *cross-sectional studies*

Section/Topic	Item #	Recommendation	Reported on page #
**Title and abstract**	1	(*a*) Indicate the study's design with a commonly used term in the title or the abstract	1
		(*b*) Provide in the abstract an informative and balanced summary of what was done and what was found	2-3
**Introduction**			
Background/rationale	2	Explain the scientific background and rationale for the investigation being reported	4-5
Objectives	3	State specific objectives, including any prespecified hypotheses	5
**Methods**			
Study design	4	Present key elements of study design early in the paper	6
Setting	5	Describe the setting, locations, and relevant dates, including periods of recruitment, exposure, follow-up, and data collection	6
Participants	6	(*a*) Give the eligibility criteria, and the sources and methods of selection of participants	7
Variables	7	Clearly define all outcomes, exposures, predictors, potential confounders, and effect modifiers. Give diagnostic criteria, if applicable	7-8
Data sources/ measurement	8*	For each variable of interest, give sources of data and details of methods of assessment (measurement). Describe comparability of assessment methods if there is more than one group	8
Bias	9	Describe any efforts to address potential sources of bias	*8*
Study size	10	Explain how the study size was arrived at	7
Quantitative variables	11	Explain how quantitative variables were handled in the analyses. If applicable, describe which groupings were chosen and why	7
Statistical methods	12	(*a*) Describe all statistical methods, including those used to control for confounding	7-8
		(*b*) Describe any methods used to examine subgroups and interactions	7-8
		(*c*) Explain how missing data were addressed	7-8
		(*d*) If applicable, describe analytical methods taking account of sampling strategy	7-8
		(*e*) Describe any sensitivity analyses	7-8
**Results**			

For peer review only

Participants	13*	(a) Report numbers of individuals at each stage of study—eg numbers potentially eligible, examined for eligibility, confirmed eligible, included in the study, completing follow-up, and analysed	9-10
		(b) Give reasons for non-participation at each stage	9-10
		(c) Consider use of a flow diagram	9-10
Descriptive data	14*	(a) Give characteristics of study participants (eg demographic, clinical, social) and information on exposures and potential confounders	10-13
		(b) Indicate number of participants with missing data for each variable of interest	10-13
Outcome data	15*	Report numbers of outcome events or summary measures	10-13
Main results	16	(*a*) Give unadjusted estimates and, if applicable, confounder-adjusted estimates and their precision (eg, 95% confidence interval). Make clear which confounders were adjusted for and why they were included	13-16
		(*b*) Report category boundaries when continuous variables were categorized	13-16
		(*c*) If relevant, consider translating estimates of relative risk into absolute risk for a meaningful time period	13-16
Other analyses	17	Report other analyses done—eg analyses of subgroups and interactions, and sensitivity analyses	
**Discussion**			
Key results	18	Summarise key results with reference to study objectives	17-19
Limitations	19	Discuss limitations of the study, taking into account sources of potential bias or imprecision. Discuss both direction and magnitude of any potential bias	17-19
Interpretation	20	Give a cautious overall interpretation of results considering objectives, limitations, multiplicity of analyses, results from similar studies, and other relevant evidence	17-19
Generalisability	21	Discuss the generalisability (external validity) of the study results	17-19
**Other information**			
Funding	22	Give the source of funding and the role of the funders for the present study and, if applicable, for the original study on which the present article is based	20

*Give information separately for cases and controls in case-control studies and, if applicable, for exposed and unexposed groups in cohort and cross-sectional studies.

Note: An Explanation and Elaboration article discusses each checklist item and gives methodological background and published examples of transparent reporting. The STROBE checklist is best used in conjunction with this article (freely available on the Web sites of PLoS Medicine at http://www.plosmedicine.org/, Annals of Internal Medicine at http://www.annals.org/, and Epidemiology at http://www.epidem.com/). Information on the STROBE Initiative is available at www.strobe-statement.org.

For peer review only

BMJ Open

Mediator or moderator? The role of obesity in the association between age at menarche and blood pressure in mid-aged and elderly Chinese: a population-based cross-sectional study

Journal:	*BMJ Open*
Manuscript ID	bmjopen-2021-051486.R2
Article Type:	Original research
Date Submitted by the Author:	12-Apr-2022
Complete List of Authors:	zhang, lin; Wannan Medical College, Department of Internal Medicine Nursing, School of Nursing, Wannan Medical College, 22 Wenchang West Road, Higher Education Park, Wuhu City, An Hui Province, P.R.China<br>Yang, Liu; Wannan Medical College, Department of Internal Medicine Nursing, School of Nursing, Wannan Medical College, 22 Wenchang West Road, Higher Education Park, Wuhu City, An Hui Province, P.R.China<br>Wang, Congzhi; Wannan Medical College, Department of Internal Medicine Nursing, School of Nursing, Wannan Medical College, 22 Wenchang West Road, Higher Education Park, Wuhu City, An Hui Province, P.R.China<br>Yuan, Ting; Wannan Medical College, Obstetrics and Gynecology Nursing, School of Nursing, Wannan Medical College, 22 Wenchang West Road, Higher Education Park, Wuhu City, An Hui Province, P.R.China<br>Zhang, Dongmei; Wannan Medical College, Department of Pediatric Nursing, School of Nursing, Wannan Medical College, 22 Wenchang West Road, Higher Education Park, Wuhu City, An Hui Province, P.R.China<br>Wei, Huanhuan; Wannan Medical College, Obstetrics and Gynecology Nursing, School of Nursing, Wannan Medical College, 22 Wenchang West Road, Higher Education Park, Wuhu City, An Hui Province, P.R.China<br>Li, Jing; Wannan Medical College, Department of Surgical Nursing, School of Nursing, Wannan Medical College, 22 Wenchang West Road, Higher Education Park, Wuhu City, An Hui Province, P.R.China<br>Lei, Yunxiao; Wannan Medical College, Obstetrics and Gynecology Nursing, School of Nursing, Wannan Medical College, 22 Wenchang West Road, Higher Education Park, Wuhu City, An Hui Province, P.R.China; Henan University of Science and Technology, School of Nursing<br>Sun, Lu; Wannan Medical College, Department of Emergency and Critical Care Nursing, School of Nursing, Wannan Medical College, 22 Wenchang West Road, Higher Education Park, Wuhu City, An Hui Province, P.R.China<br>Li, Xiaoping; Wannan Medical College, Department of Emergency and Critical Care Nursing, School of Nursing, Wannan Medical College, 22 Wenchang West Road, Higher Education Park, Wuhu City, An Hui Province, P.R.China<br>Hua, Ying; Wannan Medical College, Rehabilitation Nursing, School of Nursing, Wanna Medical college, 22 Wenchang West Road, Higher Education Park, Wuhu City, An Hui Province, P.R.China<br>Che, Hengying; Yijishan Hospital of Wannan Medical College, Department

	of nursing, Yijishan Hospital, the First Affiliated Hospital of Wannan Medical College, Zheshan West Road, Yijishan District, Wuhu City, Anhui Province<br>Li, Yuanzhen; Wannan Medical College, Department of Emergency and Critical Care Nursing, School of Nursing, Wannan Medical College, 22 Wenchang West Road, Higher Education Park, Wuhu City, An Hui Province, P.R.China
**Primary Subject Heading**:	Geriatric medicine
Secondary Subject Heading:	Public health
Keywords:	Hypertension < CARDIOLOGY, Risk management < HEALTH SERVICES ADMINISTRATION & MANAGEMENT, Nutrition < TROPICAL MEDICINE

I, the Submitting Author has the right to grant and does grant on behalf of all authors of the Work (as defined in the below author licence), an exclusive licence and/or a non-exclusive licence for contributions from authors who are: i) UK Crown employees; ii) where BMJ has agreed a CC-BY licence shall apply, and/or iii) in accordance with the terms applicable for US Federal Government officers or employees acting as part of their official duties; on a worldwide, perpetual, irrevocable, royalty-free basis to BMJ Publishing Group Ltd ("BMJ") its licensees and where the relevant Journal is co-owned by BMJ to the co-owners of the Journal, to publish the Work in this journal and any other BMJ products and to exploit all rights, as set out in our licence.

The Submitting Author accepts and understands that any supply made under these terms is made by BMJ to the Submitting Author unless you are acting as an employee on behalf of your employer or a postgraduate student of an affiliated institution which is paying any applicable article publishing charge ("APC") for Open Access articles. Where the Submitting Author wishes to make the Work available on an Open Access basis (and intends to pay the relevant APC), the terms of reuse of such Open Access shall be governed by a Creative Commons licence – details of these licences and which Creative Commons licence will apply to this Work are set out in our licence referred to above.

Other than as permitted in any relevant BMJ Author's Self Archiving Policies, I confirm this Work has not been accepted for publication elsewhere, is not being considered for publication elsewhere and does not duplicate material already published. I confirm all authors consent to publication of this Work and authorise the granting of this licence.

For peer review only

Mediator or moderator? The role of obesity in the association between age at menarche and blood pressure in mid-aged and elderly Chinese: a population-based cross-sectional study

Lin Zhang[1], Liu Yang[1], Congzhi Wang[1], Ting Yuan[2], Dongmei Zhang[3], Huanhuan Wei[2], Jing Li[4], Yunxiao Lei[2], Lu Sun[5], Xiaoping Li[5], Ying Hua[6], Hengying Che[7*], Yuanzhen Li[5]

*Correspondence: Hengying Che: 2722659079@qq.com

Lin Zhang: yaoran2008@163.com

Liu Yang: zqzqylyl5858@163.com

Congzhi Wang: 289957420@qq.com

Ting Yuan: yuanting@wnmc.edu.cn

Dongmei Zhang: dongmei.0715@163.com

Huanhuan Wei: whh0702@163.com

Jing Li: 475366616@qq.com

Yunxiao Lei: lyxheart@163.com

Lu Sun: 565196200@qq.com

Xiaoping Li：349274808@qq.com

Ying Hua: 1542997737@qq.com

Hengying Che: 2722659079@qq.com

Yuanzhen Li: yuanzhen.li@wnmc.edu.cn

[1] Department of Internal Medicine Nursing, School of Nursing, Wannan Medical College, 22 Wenchang West Road, Higher Education Park, Wuhu City, An Hui Province, P.R.China

[2] Obstetrics and Gynecology Nursing, School of Nursing, Wannan Medical College, 22 Wenchang West Road, Higher Education Park, Wuhu City, An Hui Province, P.R.China

[3]Department of Pediatric Nursing, School of Nursing, Wannan Medical College, 22 Wenchang West Road, Higher Education Park, Wuhu City, An Hui Province, P.R.China

[4] Department of Surgical Nursing, School of Nursing, Wannan Medical College, 22 Wenchang West Road, Higher Education Park, Wuhu City, An Hui Province, P.R.China

[5]Department of Emergency and Critical Care Nursing, School of Nursing, Wannan Medical College, 22 Wenchang West Road, Higher Education Park, Wuhu City, An Hui Province, P.R.China

[6]Rehabilitation Nursing, School of Nursing, Wanna Medical college, 22 Wenchang West Road, Higher Education Park, Wuhu City, An Hui Province, P.R.China

[7]Department of nursing, Yijishan Hospital, the First Affiliated Hospital of Wannan Medical College, Zheshan West Road, Yijishan District, Wuhu City, Anhui Province

For peer review only

For peer review only - http://bmjopen.bmj.com/site/about/guidelines.xhtml

ABSTRACT

Objective We investigated the moderation/mediation between the age of menarche and obesity parameters in predicting blood pressure (BP) in middle-aged and elderly Chinese.

Design Our study is a population-based cross-sectional study.

Setting Participants in this study came from the China Health and Retirement Longitudinal Study (CHARLS).

Participants The analytic sample included 4513 participants aged 45 to 96 years.

Main outcome measurements Data were selected from the CHARLS, a cross-sectional study. Between-group differences were evaluated using chi-square, t-test and one-way ANOVA. The trend of related variables by characteristics was also tested using contrast analysis, as appropriate. Then, correlations between characteristics, moderator, mediator, independent, and dependent variables were used by Spearman's correlation test and Pearson's correlation test. Finally, the mediation analysis was performed by model 4 in process v3.3, and the moderation analysis was used model 1 to assess. All covariates were adjusted in the moderation or mediation models.

Results In correlation analysis, body mass index (BMI) and waist circle (WC) level were positively correlated with both systolic blood pressure (SBP) and diastolic blood pressure (DBP) in women (BMI and DBP: r =0.221, *P* <0.001; WC and DBP: r =0.183, *P* <0.001; BMI and SBP: r =0.129, *P* <0.001; WC and SBP: r =0.177, *P* <0.001). Age of menarche was negatively correlated with DBP (r =-0.060, *P* <0.001). However, the age of menarche was not significantly correlated with SBP (r =-0.014, *P* =0.335). In the moderator analysis, after controlling for the potential confounders, the interaction term of obesity parameters*age of menarche was not significant for predicting either DBP (BMI: B = 0.0260, SE = 0.0229, *P* =0.2556, 95% CI [-0.0189,0.071]); WC: B = 0.0099, SE =0.0074, *P* =0.1833, 95% CI [-0.0047,0.0244]) or SBP (BMI: B =0.0091, SE =0.0504, *P* =0.8561, 95% CI [-0.0897,0.108]); WC: B = -0.0032, SE =0.0159, *P* =0.8427, 95% CI [-0.0343,0.028]). All correlations were significant correlation between age of menarche, obesity parameters and BP except the path of the menarche age →SBP (with the addition of the BMI indicator: β =-0.0004, B =-0.0046, *P* =0.9797, 95% CI [-0.3619,0.3526]); with the addition of the WC indicator: β = 0.0004, B =0.0044, *P* =0.9804, 95% CI [-0.3439, 0.3526]) in crude model. In general, after controlling for potential confounders. BMI (DBP: β = -0.0471, B= -0.2682, *P* =0.0021, 95% CI [-0.4388, -0.0976]; SBP: β =-0.0515, B =-0.6314, *P* <0.001, 95% CI [-0.9889, -0.2739]) and WC (DBP: β = -0.0474, B= -0.2689, *P* <0.001, 95% CI [-0.4395, -0.0984]; SBP: β =-0.0524, B =-0.6320, *P* <0.001, 95% CI [-0.9832, -0.2807]) partly

For peer review only - http://bmjopen.bmj.com/site/about/guidelines.xhtml

mediated the relationship between age of menarche and BP.

Conclusion The interaction term of obesity parameters*age of menarche was not significant for predicting either DBP or SBP in women. Moreover, obesity parameters partly mediated the relationship between the age of menarche and BP.

Keywords age at menarche, blood pressure, body mass index, moderator, mediator, obesity parameters, waist circle

Strengths and limitations of the study

This is the first large population study to examine the moderation between the age of menarche and obesity parameters in predicting blood pressure (BP) in middle-aged and elderly Chinese, as well as the mediation effects of obesity parameters on the relationship between age of menarche and BP.

This study included a large sample of 4513 middle-aged and older Chinese. The results presented in this article could be explored further in prospective cohort studies.

The main limitations of our research are related to the cross-sectional study and the self-reported method used for the assessment of age at menarche and most related confounders.

Blood pressure in our study was measured at home by professionally trained volunteers, and the next step is the use of clinical blood pressure and ambulatory blood pressure.

For peer review only

INTRODUCTION

Blood pressure (BP) is the force of circulating blood against the walls of the body's arteries, the major blood vessels in the body. Hypertension is diagnosed if BP readings are 140/90 mmHg or above on two different days. As a chronic disease[1], hypertension or elevated BP is a severe medical condition that significantly increases heart attack, stroke, blindness, kidney failure, and other complications. Hypertension risk factors[2-6] include stress, salt consumption, harmful use of alcohol, low intake of fruits and vegetables, saturated fat and trans fats, being overweight or obese, tobacco use, low diet in vitamin D, lack of physical activity, family history, aged 65 years or over and other co-existing diseases. The World Health Organization(WHO) said[7] an estimated 1.13 billion people worldwide have hypertension and most (two-thirds) living in low- and middle-income countries. It means that most people have high BP. Compared to the global average, people in low- and middle-income countries may bear a disproportionate and heavier burden of the disease, owing to several factors such as the ongoing nutritional transition in dietary consumption and energy expenditure, increasing trends in sedentary lifestyle, and other modifiable risk factors, and inadequate health care systems[8]. As a developing country, China has shown a relatively high and stable prevalence of hypertension among adults, especially in middle-aged and older people. Mahajan S, *et al.* [9] showed the prevalence of hypertension among middle-aged and elderly Chinese was approximately 41.81% in 2015. However, the rate control was still below 30%. Recently, hypertension control has become one of the critical public health interventions[10]. Therefore, it emphasizes the promotion of public health strategy to prevent the significant hypertension risk factors and assure the fullest available accessibility and use of quality health control for people with risk factors or who develop subclinical or overt hypertension. These actions are integral to a comprehensive public health strategy for hypertension promotion and prevention[11-13]. Though there is still much uncertainty about the etiology of hypertension, one of the strongest risk factors was overweight/obesity. Thus, increased body mass index (BMI) or centrally located body fat (especially waist circle [WC]) increases the risk of developing hypertension.

Several studies[14-18]have reported that early biological maturation has been associated with obesity and BP in adolescents. Werneck AO *et al.* [14] showed that behavioral and hereditary variables are more related to BP in late-maturing adolescents in adolescents. Widen E, *et al.*[15] found that earlier pubertal timing was associated with higher adult BMI and diastolic blood pressure (DBP) in both sexes. Mueller NT, *et al.* [16] showed that earlier menarche was associated with higher waist

For peer review only - http://bmjopen.bmj.com/site/about/guidelines.xhtml

circumference and BMI measured. Dreyfus J, *et al.*[17] found that earlier age at menarche was associated with higher mean BMI among African American and White women. Cao M, *et al.*[18] found that earlier age at menarche was associated with decreased DBP. However, it is not completed understood whether the changes in biological processes in the early years can affect cardiovascular outcomes across the life span. As important indicators of obesity, BMI/WC is directly associated with biological maturation (based on menarche age). Evidence from several studies[19-21] indicated that menarche was related to BMI in later life. Power C, *et al.*[19] found that timing of puberty was strongly associated with BMI for the earlier matures at ages 7-33 years. Adair LS, *et al.*[20] showed that overweight prevalence rates were significantly higher in early maturing adolescents. Must A, *et al.*[21] showed that early maturation in adult females overweight is mostly a result of the influence of elevated relative weight on early maturation. It has been speculated that the association between biological maturation and hypertension risk factors (based on menarche age) can be mediated or moderated by obesity parameters. Although speculative, the mediating or moderating role of obesity on the effect of early maturation (based on menarche age) on BP in adulthood has not been classified. However, this is especially important given that the onset of biological maturation, especially the age of menarche, now occurs at a much earlier age than in previous studies. Moreover, fully understanding the mediating effect of obesity on the association between early maturation (based on menarche age) and BP in adulthood could help identify women group that are at risk (based on obesity parameters) and those that should be put forward designed to place the many diverse conceptual and practice approaches and accomplishments in the early intervention. The moderating effect between early maturation (based on menarche age) and risk of hypertension (obesity parameters) on BP may also be fully considered.

To date, few studies on the mediating and moderating analysis between early maturation (based on menarche age) and risk of hypertension (obesity parameters) were conducted in individuals aged ≥45 years. Thus, this study aimed to determine their association with age of menarche, obesity parameters, and other confounding factors using the China Health and Retirement Longitudinal Study (CHARLS) from participants aged ≥45 years in China.

METHODS

Study design and setting

Data from CHARLS Wave 1 (2011) were used in our research. The CHARLS is an ongoing national

For peer review only - http://bmjopen.bmj.com/site/about/guidelines.xhtml

longitudinal study administered by the National School for Development (China Center for Economic Research)[22] from 2011. The study was approved by the institutional ethical committees of Peking University. All participants provided informed consent.

Individuals

At baseline[23], 6883 women were recruited for a longitudinal study, 2370 individuals were excluded (i.e., absence of metabolic measures, and/or absence of medication history, and/or having no blood pressure, and/or using antihypertension drugs). Finally, 4513 individuals were included in the analyses. Figure 1 summarized the selection of participants. Our study involved 4513 participants aged ≥45 years were total women [mean ± standard deviation age=58.59 ± 9.31years, ranged from 45 to 96years]. The mean and standard deviation of menarche age were 16.27±2.18 years (ranged from 10 to 30 years).

Self-report of risk factors

We employed a general sociodemographic questionnaire that included the age, age of menarche (≤14years, 15years, 16years, 17years, and ≥18years), educational attainment (illiterate, less than elementary school, high school, and above vocational school), marital status (single and married), and region (rural and urban). The lifestyle questionnaire included smoking status (never smoker, previous smoker, and current smoker), alcohol consumption (never drinker, less than once a month and more than once a month), eating habits (≤2 meals per day, 3 meals per day, and ≥4 meals per day), entertainment, the experience of a traumatic event (no and yes), and physical exercise habit (no, less than regular physical exercises, and regular physical exercises). History of cardiovascular diseases and liver disease, antihyperlipidemic drugs, and antidiabetic drugs were categorized in 'yes' or 'no'. Most variables were used in our previous studies[24-30].

Measurements

Triglycerides (TG) level, low-density lipoprotein cholesterol (LDL-C), high-density lipoprotein cholesterol (HDL-C), and glycosylated hemoglobin (HbA1c) were analyzed by the enzymatic colorimetric tests, serum creatinine (Scr) and serum uric acid (SUA) level were analyzed using the urinalysis (UA) plus method. The average value of BP was measured by the mean of the 3-time measurements using Omron hem-7200 sphygmomanometer. Height was measured by the height

For peer review only - http://bmjopen.bmj.com/site/about/guidelines.xhtml

For peer review only

measurement instrument. Participants were asked to stand barefoot on the base plate of the height meter in the upright position, with the back against the vertical backplate of the measuring instrument, with eyes open, arms alongside the body and heels were closed and the toes were separated by 60 degrees. Anyone who appeared to unable to complete measurement in time due to health reasons such as hunchback and inability to stand was excluded from our study. The method was used in our previous study[30]. BMI was calculated as body weight (kg) divided by the square of the subject's height (m). WC was measured midway between spina iliaca superior and the lower rib margin at the end of exhalation.

Statistical analysis

The data were presented as means(M) and standard deviation (SD) unless indicated otherwise. Mean and standard deviation were used to describe continuous variables (age, age of menarche, LDL-C, HDL-C, TG, HbA1c, Scr, SUA, BMI, WC, DBP, and SBP level), and number and percentage were used to assess the categorical variables (educational attainment, marital status, region, alcohol consumption, smoking status, eating habits, entertainment, the experience of a traumatic event, physical activity, history of CVDs, history of liver diseases, history of antilipidemic medication, anti-diabetic medication). Between-group differences according to the age of menarche (≤14years, 15years, 16years, 17years, ≥18years) were evaluated by the ANOVA (continuous data) or chi-square test (categorical data). Differences between two categories of characteristics (marital status, region, entertainment, the experience of a traumatic event, history of CVDs, history of antilipidemic medication, history of liver diseases, anti-diabetic medication) were also evaluated using t-test. However, the trend of related variables (LDL-C, HDL-C, LDL-C/HDL-C, TG, HbA1c, Scr, SUA, BMI, WC, DBP, and SBP level) by above three categories of characteristics (age of menarche levels, educational attainment, alcohol consumption, smoking status, eating habits, physical activity) was also tested using contrast analysis. Correlations between characteristics and BP were used by Spearman's correlation test. However, relationships between continuous variables (age, LDL-C, HDL-C, LDL-C/HDL-C, TG, HbA1c, Scr, SUA, BMI, WC and menarche age) and BP were used by Pearson's correlation test. The mediation analysis was performed by model 4 in process v3.3[31-33], and the moderation analysis was used model 1 to assess. All covariates (including age, educational attainment, marital status, region, smoking status, alcohol consumption, eating habits, entertainment, the experience of a traumatic event, physical activity, history of cardiovascular diseases, hepatitis history, antihyperlipidemic drugs, antidiabetic drugs, LDL-C, HDL-C, TG, HbA1c, Scr, SUA) were

For peer review only - http://bmjopen.bmj.com/site/about/guidelines.xhtml

adjusted for in the mediation or moderation models. Analyses including the unstandardized (B), Standard Error (SE), or standardized coefficients (β) with 95% confidence intervals(95%CI) were calculated using the setting of modelling building in IBM SPSS22.0 software for Windows10 (IBM Corp., Armonk, NY, USA) using the bootstrap method, with a 5% significance level.

Patient and public involvement

No patients were involved in the development of the question, design or data interpretation.

For peer review only

For peer review only - http://bmjopen.bmj.com/site/about/guidelines.xhtml

RESULTS

The final sample consisted of 4513 women with an average age of 58.59 ± 9.31years (ranged from 45 to 96 years). The mean and standard deviation of menarche age were 16.27±2.18 years (ranged from 10 to 30 years). The mean and standard deviation of DBP and SBP level were 76.31±12.45mmHg (ranged from 39 to 136 mmHg), and 131.65±26.73 mmHg (ranged from 76 to 235 mmHg), respectively. The mean and standard deviation of BMI and WC level were 24.04±4.13 kg/m^2(ranged from 11.65 to 63.05 kg/m^2), and 84.67±12.83cm (ranged from 19.5 to 130.10 cm), respectively. Table 1 showed the characteristics by the age of menarche. There were differences between the age of menarche in educational attainment, region, cigarette smoking, entertainment, and history of CVDs. However, between-group differences in the prevalence of marital status, alcohol consumption, eating habits, the experience of a traumatic event, physical activity, hepatitis history, antilipidemic medication history, and antidiabetic medication history were not found. Women with a higher age of menarche had higher HDL-C level and age but had lower TG, SUA, BMI, WC, and DBP level. Moreover, there were no differences in LDL-C, LDL-C/HDL-C, HbA1c, Scr, and SBP level between the age of menarche groups.

Table1 Characteristics by age at menarche (N=4513)

Variables	≤14(years) N=946	15(years) N=671	16(years) N=930	17(years) N=702	≥18(years) N=1264	*P*
Age(years)	56.88±9.38	57.49±9.34	58.41±9.42	58.6±9.12	60.57±8.91	<0.001[a]
Educational attainment						
Illiterate	300(31.71)	233(34.72)	378(40.65)	314(44.73)	649(51.34)	<0.001
Less than elementary school	527(55.71)	383(57.08)	486(52.26)	343(48.86)	572(45.25)	
High school	82(8.67)	42(6.26)	48(5.16)	34(4.84)	34(2.69)	
Above vocational school	37(3.91)	13(1.94)	18(1.94)	11(1.57)	9(0.71)	
Marital status						
Single	137(14.48)	79(11.77)	137(14.73)	98(13.96)	207(16.38)	0.101
Married	809(85.52)	592(88.23)	793(85.27)	604(86.04)	1057(83.62)	
Region						
Rural	519(54.86)	426(63.49)	577(62.04)	454(64.67)	885(70.02)	<0.001
Urban	427(45.14)	245(36.51)	353(37.96)	248(35.33)	379(29.98)	
Cigarette smoking						
Never smoker	883(93.34)	629(93.74)	846(90.97)	646(92.02)	1156(91.46)	0.017
Previous smoker	8(0.85)	11(1.64)	18(1.94)	13(1.85)	38(3.01)	
Current smoker	55(5.81)	31(4.62)	66(7.1)	43(6.13)	70(5.54)	
Alcohol consumption						
Never drinker	827(87.42)	607(90.46)	809(86.99)	620(88.32)	1100(87.03)	0.520
Less than once a month	45(4.76)	26(3.87)	48(5.16)	37(5.27)	65(5.14)	
More than once a month	74(7.82)	38(5.66)	73(7.85)	45(6.41)	99(7.83)	
Eating habits						
≤2 meals per day	131(13.85)	99(14.75)	123(13.23)	82(11.68)	172(13.61)	0.706
3 meals per day	804(84.99)	567(84.5)	794(85.38)	609(86.75)	1073(84.89)	
≥4 meals per day	11(1.16)	5(0.75)	13(1.40)	11(1.57)	19(1.50)	
Entertainment						
No	417(44.08)	335(49.93)	470(50.54)	372(52.99)	655(51.82)	0.002
Yes	529(55.92)	336(50.07)	460(49.46)	330(47.01)	609(48.18)	
The experience of a traumatic event						
No	881(93.13)	630(93.89)	867(93.23)	652(92.88)	1169(92.48)	0.837
Yes	65(6.87)	41(6.11)	63(6.77)	50(7.12)	95(7.52)	
Physical activity						
No physical exercise	587(62.05)	393(58.57)	556(59.78)	428(60.97)	771(61.00)	0.260
Less than regular physical exercises	178(18.82)	137(20.42)	171(18.39)	136(19.37)	271(21.44)	
Regular physical exercises	181(19.13)	141(21.01)	203(21.83)	138(19.66)	222(17.56)	
History of CVDs						
No	810(85.62)	590(87.93)	780(83.87)	605(86.18)	1115(88.21)	0.033
Yes	136(14.38)	81(12.07)	150(16.13)	97(13.82)	149(11.79)	

For peer review only - http://bmjopen.bmj.com/site/about/guidelines.xhtml

Hepatitis history						
No	910(96.19)	647(96.42)	885(95.16)	670(95.44)	1226(96.99)	0.205
Yes	36(3.81)	24(3.58)	45(4.84)	32(4.56)	38(3.01)	
History of antilipidemic medication						
No	884(93.45)	628(93.59)	884(95.05)	656(93.45)	1184(93.67)	0.566
Yes	62(6.55)	43(6.41)	46(4.95)	46(6.55)	80(6.33)	
History of antidiabetic medication						
No	910(96.19)	645(96.13)	883(94.95)	663(94.44)	1213(95.97)	0.309
Yes	36(3.81)	26(3.87)	47(5.05)	39(5.56)	51(4.03)	
LDL-C (mg/dL)	119.66±35.04	118.04±35.61	120.62±34.81	121.79±36.79	121.45±35.27	0.065 [a]
HDL-C (mg/dL)	50.57±14.17	51.31±14.01	50.84±14.43	51.05±14.18	53.2±14.87	<0.001 [a]
LDL-C/HDL-C	2.50±0.90	2.42±0.89	2.52±0.91	2.51±0.92	2.43±0.92	0.242 [a]
TG (mg/dL)	143.09±99.89	142.86±103.67	142.07±98.31	145.85±132.3	124.16±76.25	<0.001 [a]
HbA1c (%)	5.31±0.83	5.25±0.86	5.31±0.89	5.32±0.84	5.27±0.76	0.594 [a]
Scr (mg/dL)	0.69±0.14	0.69±0.14	0.70±0.18	0.68±0.13	0.69±0.15	0.813 [a]
SUA (mg/dL)	4.09±1.10	3.99±1.09	4.04±1.07	4.01±1.01	3.97±1.05	0.021 [a]
BMI (kg/m^2)	24.63±4.11	24.39±4.15	24.02±3.90	24.18±4.49	23.36±3.99	<0.001 [a]
WC (cm)	86.08±13.01	85.35±12.62	85.29±11.33	84.53±13.38	82.89±13.36	<0.001 [a]
DBP (mmHg)	77.07±12.07	77.28±12.53	77.01±12.51	76.31±12.80	74.72±25.82	<0.001 [a]
SBP (mmHg)	131.09±26.36	131.66±25.49	131.63±26.64	129.90±29.9	129.47±25.82	0.062 [a]

CVDs: cardiovascular diseases; LDL-C: low-density lipoprotein cholesterol; HDL-C: high-density lipoprotein cholesterol; TG: triglycerides; HbA1c: Hemoglobin A1C; Scr: serum creatinine; SUA: serum uric acid; BMI: body mass index; WC: waist circle; DBP: diastolic blood pressure; SBP: systolic blood pressure.

[a]*P*-value for trend

Characteristics, according to BMI and WC in women, were presented in Table 2. Firstly, women with the place in rural, more alcohol consumption, higher age of menarche had lower BMI and WC level; Secondly, women with a history of CVDs, antilipidemic medication, and antidiabetic medication had higher BMI and WC level than women without medical illness. Thirdly, women with higher educational attainment, being married, being never smoking, taking more eating meals, and taking entertainment had higher BMI level. At last, women with the experience of a traumatic event had lower WC level.

Table2 Characteristics of the participants with BMI and WC (N=4513)

Variables	BMI (cm)	WC (cm)	*P*-value for BMI	*P*-value for WC
Educational attainment				
Illiterate	23.57±4.19	84.35±12.95	0.001 [a]	0.971 [a]
Less than elementary school	24.31±4.03	85.14±12.48		
High school	24.93±4.07	83.17±14.65		
Above vocational school	24.85±4.43	83.40±13.99		
Marital status				
Single	23.21±4.18	84.93±12.04	<0.001	0.582
Married	24.19±4.10	84.63±12.97		
Region				
Rural	23.64±3.96	83.95±12.42	<0.001	<0.001
Urban	24.75±4.32	85.94±13.43		
Cigarette smoking				
Never smoker	24.11±4.12	84.73±12.77	<0.001 [a]	0.135 [a]
Previous smoker	23.79±4.14	86.83±10.36		
Current smoker	23.17±4.11	83.13±14.40		
Alcohol consumption				
Never drinker	24.10±4.14	85.02±12.5	0.011 [a]	<0.001 [a]
Less than once a month	23.88±4.17	81.78±15.16		
More than once a month	23.51±3.94	82.49±14.62		
Eating habits				
≤2 meals per day	23.27±3.89	84.12±10.42	<0.001 [a]	0.734 [a]
3 meals per day	24.18±4.14	84.82±13.18		
≥4 meals per day	23.10±4.69	80.82±12.01		
Entertainment				
No	23.64±4.09	84.34±12.08	<0.001	0.085
Yes	24.45±4.13	85.00±13.53		
The experience of a traumatic event				
No	24.06±4.14	84.82±12.57	0.474	0.004
Yes	23.88±3.94	82.64±15.81		
Physical activity				
No physical exercise	24.05±4.29	84.79±12.74	0.400 [a]	0.701 [a]
Less than regular physical exercises	23.82±3.70	84.25±12.48		
Regular physical exercises	24.26±4.03	84.74±13.48		

For peer review only - http://bmjopen.bmj.com/site/about/guidelines.xhtml

History of CVDs				
No	23.92±4.09	84.26±12.63	<0.001	<0.001
Yes	24.83±4.28	87.25±13.89		
Hepatitis history				
No	24.04±4.15	84.66±12.79	0..871	0.948
Yes	24.09±3.47	84.73±14.37		
History of antilipidemic medication				
No	23.87±3.99	84.22±12.72	<0.001	<0.001
Yes	26.75±5.14	91.59±12.58		
History of antidiabetic medication				
No	23.95±4.01	84.42±12.81	<0.001	<0.001
Yes	26.13±5.81	90.18±12.20		
Menarche age(years)				
≤14	24.63±4.11	86.08±13.01	<0.001 [a]	<0.001 [a]
15	24.39±4.15	85.35±12.62		
16	24.02±3.90	85.29±11.33		
17	24.18±4.49	84.53±13.38		
≥18	23.36±13.36	82.89±13.36		

CVDs: cardiovascular diseases; BMI: body mass index; WC: waist circle.

[a]*P*-value for trend

Characteristics, according to DBP and SBP in women, were presented in Table 3. Firstly, women with more alcohol consumption had lower DBP and SBP level; Secondly, women with a history of CVDs and antilipidemic medication had higher DBP and SBP level than women without medical illness. Thirdly, women with more eating meals, higher age of menarches had lower DBP level. At last, women with higher educational attainment, being married, and having regular physical exercises had lower SBP level, but women with antidiabetic medication had higher SBP level than women without medical illness.

Table3 Table2 Characteristics of the participants with DBP and SBP (N=4513)

Variables	DBP(mmHg)	SBP(mmHg)	*P*-value for DBP	*P*-value for SBP
Educational attainment				
Illiterate	76.37±12.45	133.93±28.43	0.288[a]	<0.001[a]
Less than elementary school	76.38±12.42	128.89±24.99		
High school	76.12±12.65	122.98±18.58		
Above vocational school	73.86±12.47	127.84±40.38		
Marital status				
Single	76.70±11.96	139.61±31.93	0.388	<0.001
Married	76.25±12.53	129.12±25.42		
Region				
Rural	76.20±12.72	130.18±25.92	0.435	0.121
Urban	76.50±11.96	131.46±28.06		
Cigarette smoking				
Never smoker	76.32±12.45	130.43±26.97	0.673[a]	0.051[a]
Previous smoker	73.60±11.41	132.13±22.83		
Current smoker	77.09±12.65	133.61±23.78		
Alcohol consumption				
Never drinker	76.53±12.38	131.27±27.27	0.003[a]	0.001[a]
Less than once a month	74.76±12.18	123.81±19.94		
More than once a month	74.73±13.26	127.77±23.06		
Eating habits				
≤2 meals per day	77.69±12.60	132.14±26.68	<0.001 [a]	0.117 [a]
3 meals per day	76.17±12.41	130.44±26.37		
≥4 meals per day	71.56±11.46	128.63±44.41		
Entertainment				
No	76.08±12.60	130.66±26.33	0.216	0.975
Yes	76.54±12.30	130.64±27.12		
The experience of a traumatic event				
No	76.38±12.47	130.84±27.12	0.219	0.075
Yes	75.48±12.14	128.05±20.66		
Physical activity				
No physical exercise	76.34±12.46	131.47±28.25	0.873 [a]	0.024 [a]
Less than regular physical exercises	76.05±12.76	129.23±22.14		
Regular physical exercises	76.50±12.08	129.54±26.03		
History of CVDs				
No	76.11±12.44	130.02±26.29	0.021	<0.001

For peer review only - http://bmjopen.bmj.com/site/about/guidelines.xhtml

Yes	77.39±12.40	134.50±29.28		
Hepatitis history				
No	76.36±12.45	130.76±26.50	0.017	0.144
Yes	74.10±11.56	127.76±32.79		
History of antilipidemic medication				
No	76.07±12.34	130.12±26.01	<0.001	<0.001
Yes	80.09±13.46	138.80±35.01		
History of antidiabetic medication				
No	76.25±12.47	130.17±25.46	0.100	<0.001
Yes	77.73±11.83	140.96±45.17		
Menarche age(years)				
≤14	77.07±12.07	131.09±26.36		
15	77.28±12.53	131.66±25.49	<0.001 [a]	0.062 [a]
16	77.01±12.51	131.63±26.64		
17	76.31±12.80	129.90±29.90		
≥18	74.72±12.31	129.47±25.82		

CVDs: cardiovascular diseases; DBP: diastolic blood pressure; SBP: systolic blood pressure.

[a] *P*-value for trend

Correlations between characteristics, independent, moderators, mediators and dependent variables, according to DBP and SBP, were presented in Table 4. Firstly, drinking, history of CVDs, antilipidemic medication, LDL-C, HDL-C, LDL-C/HDL-C, TG, HbA_1c, Scr, SUA, BMI, and WC were significantly correlated with DBP and SBP in women; Secondly, eating habits, history of liver diseases, and menarche age were significantly correlated with DBP; At last, age, educational attainment, marital status, physical exercises, and antidiabetic medication were significantly correlated with SBP.

Table4 Relationship between various characteristics and blood pressure status of participants (N=4513)

Variables	DBP r(*P*)	SBP r(*P*)
Age	-0.016(0.275)	0.259(<0.001)
Educational attainment	-0.016(0.288)	-0.106(<0.001)
Marital status	-0.013(0.388)	-0.139(<0.001)
Current residence	0.012(0.435)	0.023(0.121)
Smoking status	0.006(0.673)	0.029(0.051)
Drinking	-0.045(0.003)	-0.051(0.001)
Eating habits	-0.055(<0.001)	-0.023(0.117)
Entertainment	0.018(0.216)	0.000(0.975)
The experience of a traumatic event	-0.018(0.219)	-0.027(0.075)
Physical exercises	0.002(0.873)	-0.034(0.024)
History of CVDs	0.035(0.018)	0.058(<0.001)
History of liver diseases	-0.035(0.018)	-0.022(0.146)
Antilipidemic medication	0.078(<0.001)	0.078(<0.001)
Antidiabetic medication	0.025(0.100)	0.083(<0.001)
LDL-C	0.050(0.001)	0.060(<0.001)
HDL-C	-0.099(<0.001)	-0.062(<0.001)
LDL-C/HDL-C	0.106(<0.001)	0.094(<0.001)
TG	0.100(<0.001)	0.091(<0.001)
HbA_1c	0.061(<0.001)	0.066(<0.001)
Scr	0.056(<0.001)	0.076(<0.001)

For peer review only

For peer review only - http://bmjopen.bmj.com/site/about/guidelines.xhtml

SUA	0.085(<0.001)	0.111(<0.001)
BMI	0.221(<0.001)	0.129(<0.001)
WC	0.183(<0.001)	0.177(<0.001)
Menarche age	-0.060(<0.001)	-0.014(0.335)

CVDs: cardiovascular diseases; LDL-C: low-density lipoprotein cholesterol; HDL-C: high-density lipoprotein cholesterol; TG: triglycerides; HbA1c: Hemoglobin A1C; Scr: serum creatinine; SUA: serum uric acid; BMI: body mass index; WC: waist circle; DBP: diastolic blood pressure; SBP: systolic blood pressure.

In the moderator analysis, we tested the interaction between menarche age and obesity in predicting systolic and diastolic BP. The standard error, standardized coefficients and the adjusting associated 95% confidence intervals (CIs) are shown in Table 5. We controlled for socio-demographic characteristics, health behaviors, medical histories, and metabolic measures. After controlling for the total potential confounders, the moderating effect of obesity indicators on the relationship between age of menarche and BP was tested. The interaction term of obesity parameters*age of menarche was not significant for predicting either DBP (BMI, adjusted P= 0.2556; WC, adjusted P= 0.1833) or SBP (BMI, adjusted P= 0.8561; WC, adjusted P= 0.8427) in women.

For peer review only

For peer review only - http://bmjopen.bmj.com/site/about/guidelines.xhtml

Table5 Moderator effect of obesity on the relationship between menarche age and BP in women (N=4513)

Dependent	Variables	Model one[a]			Model two[b]			Model three[c]			Model four[d]			Model five[e]		
		SE	B (95%*CI*)	*P*	se	B (95%*CI*)	*P*	SE	B (95%*CI*)	*P*	SE	B (95%*CI*)	*P*	se	B (95%*CI*)	*P*
DBP	Menarche age	0.0835	-0.2027(-0.3665, -0.0390)	0.0153	0.0855	-0.2528(-0.4203, -0.0852)	0.0031	0.0853	-0.2411(-0.4084, -0.0738)	0.0047	0.0854	-0.2485(-0.4158, -0.0811)	0.0036	0.0973	-0.2162(-0.4069, -0.0254)	0.0263
	BMI	0.0440	0.6546(0.5684,0.7409)	<0.001	0.0447	0.6757(0.5881,0.7634)	<0.001	0.0449	0.6835(0.5955,0.7715)	<0.001	0.0458	0.6647(0.5750,0.7544)	<0.001	0.0556	0.5916(0.4827,0.7005)	<0.001
	BMI× Menarche age	0.0204	0.0279(-0.0121,0.0678)	0.1719	0.0204	0.0258(-0.0141,0.0658)	0.2046	0.0203	0.0247(-0.0152,0.0645)	0.2248	0.0203	0.0220(-0.0178,0.0618)	0.2790	0.0229	0.0260(-0.0189,0.071)	0.2556
SBP	Menarche age	0.1826	-0.0003(-0.3583,0.3576)	0.9986	0.1789	-0.5822(-0.9329, -0.2315)	0.0011	0.1788	-0.5682(-0.9187, -0.2177)	0.0015	0.1795	-0.5944(-0.9462, -0.2425)	<0.001	0.2141	-0.5084(-0.9282, -0.0886)	0.0176
	BMI	0.0964	0.8361(0.6470,1.0251)	<0.001	0.0938	1.0927(0.9088,1.2766)	<0.001	0.0942	1.0906(0.9058,1.2753)	<0.001	0.0965	1.0287(0.8396,1.2178)	<0.001	0.1223	0.8846(0.6448,1.1244)	<0.001
	BMI× Menarche age	0.0446	0.0178(-0.0696,0.1052)	0.6896	0.0426	0.0111(-0.0726,0.0947)	0.7955	0.0426	0.0113(-0.0722,0.0948)	0.7914	0.0427	0.0052(-0.0786,0.0889)	0.9038	0.0504	0.0091(-0.0897,0.108)	0.8561
DBP	Menarche age	0.0838	-0.2569(-0.4213, -0.0925)	0.0022	0.0861	-0.2588(-0.4277, -0.0900)	0.0027	0.0861	-0.2481(-0.4168, -0.0794)	0.0040	0.0861	-0.2561(-0.4248, -0.0873)	0.0029	0.0979	-0.2001(-0.3921, -0.0082)	0.0410
	WC	0.0142	0.1732(0.1452,0.2011)	<0.001	0.0143	0.1752(0.1471,0.2032)	<0.001	0.0143	0.1730(0.1449,0.2011)	<0.001	0.0145	0.1645(0.1361,0.193)	<0.001	0.0172	0.1472(0.1135,0.1809)	<0.001
	WC× Menarche age	0.0065	0.0058(-0.007,0.0186)	0.3758	0.0066	0.0054(-0.0075,0.0182)	0.4136	0.0065	0.005(-0.0079,0.0178)	0.4473	0.0065	0.0043(-0.0085,0.0171)	0.5075	0.0074	0.0099(-0.0047,0.0244)	0.1833
SBP	Menarche age	0.1777	-0.0009(-0.3492,0.3475)	0.9961	0.1761	-0.5656(-0.9107, -0.2204)	0.0013	0.1760	-0.5546(-0.8997, -0.2094)	0.0016	0.1766	-0.581(-0.9273, -0.2347)	0.0010	0.2096	-0.4548(-0.8658, -0.0438)	0.0301
	WC	0.0302	0.3637(0.3044,0.4229)	<0.001	0.0293	0.3228(0.2654,0.3802)	<0.001	0.0294	0.3153(0.2578,0.3729)	<0.001	0.0298	0.2944(0.236,0.3528)	<0.001	0.0368	0.2744(0.2022,0.3466)	<0.001
	WC× Menarche age	0.0139	-0.0156(-0.0428,0.0116)	0.2607	0.0134	-0.0061(-0.0323,0.0202)	0.6501	0.0134	-0.0063(-0.0326,0.0199)	0.6367	0.0134	-0.0081(-0.0344,0.0182)	0.5443	0.0159	-0.0032(-0.0343,0.028)	0.8427

CVDs: cardiovascular diseases; LDL-C: low-density lipoprotein cholesterol; HDL-C: high-density lipoprotein cholesterol; TG: triglycerides; HbA1c: Hemoglobin A1C; Scr: serum creatinine; SUA: serum uric acid; BMI: body mass index; WC: waist circle; DBP: diastolic blood pressure; SBP: systolic blood pressure; Un-standardized: B; Standard Error: SE.

[a] **Unadjusted;**

[b] **Adjusted for** age, educational attainment, marital status, region;

[c] **Adjusted for** age, educational attainment, marital status, region, smoking status, alcohol consumption, eating habits, entertainment, experience of a traumatic event, physical activity;

[d] **Adjusted for** age, educational attainment, marital status, region, smoking status, alcohol consumption, eating habits, entertainment, experience of a traumatic event, physical activity, history of CVDs, hepatitis history, antihyperlipidemic drugs, antidiabetic drugs;

[e] **Adjusted for** age, educational attainment, marital status, region, smoking status, alcohol consumption, eating habits, entertainment, experience of a traumatic event, physical activity, history of CVDs, hepatitis history, antihyperlipidemic drugs, antidiabetic drugs, LDL-C, HDL-C, TG, HbA1c, Scr, SUA.

For peer review only

In the mediation analysis, we estimated the BP equation using bootstrap inference for model coefficients. The un-standardized, standardized coefficients and the adjusting associated 95% confidence intervals (CIs) are shown in Table 6. We controlled for socio-demographic characteristics, health behaviors, medical histories, and metabolic measures. After controlling for the total potential confounders, the mediating effect of obesity indicators on the relationship between age of menarche and BP was tested. All correlations were significant correlation between age of menarche, obesity parameters and BP except the path of the age of menarche→SBP (BMI, crude P=0.9797; WC, crude P= 0.9804) in model one. In general, after controlling for potential confounders. BMI (DBP: adjusted P=0.0021, SBP: adjusted P <0.001) and WC (DBP: adjusted P=0.0020, SBP: adjusted P <0.001) partly mediated the relationship between the age of menarche and BP.

For peer review only

For peer review only - http://bmjopen.bmj.com/site/about/guidelines.xhtml

Table6 Mediation effect of obesity on the relationship between age at menarche and BP in women (N=4513)

Path		Model one[a]			Model two[b]			Model three[c]			Model four[d]			Model five[e]		
		β	B (95%*CI*)	*P*	β	B (95%*CI*)	*P*	β	B (95%*CI*)	*P*	β	B (95%*CI*)	*P*	β	B (95%*CI*)	*P*
**Path1**	Menarche age→BMI	-0.1080	-0.2045(-0.2595, -0.1496)	<0.001	-0.0725	-0.1374(-0.1930, -0.0818)	<0.001	-0.0707	-0.1339(-0.1892, -0.0786)	<0.001	-0.0705	-0.1334(-0.1880, -0.0788)	<0.001	-0.0468	-0.0885(-0.1409, -0.0361)	<0.001
	Menarche age→DBP	-0.0367	-0.2096(-0.3731, -0.0461)	0.0120	-0.0454	-0.2596(-0.4268, -0.0924)	0.0024	-0.0433	-0.2475(-0.4145, -0.0806)	0.0037	-0.0447	-0.2542(-0.4213, -0.0872)	0.0029	-0.0379	-0.2154(-0.3833, -0.0474)	0.0120
	BMI→DBP	0.2171	0.6545(0.5682,0.7408)	<0.001	0.2241	0.6757(0.5881, 0.7634)	<0.001	0.2276	0.6835(0.5955, 0.7715)	<0.001	0.2208	0.6643(0.5746,0.7541)	<0.001	0.1986	0.5973(0.5026,0.6921)	<0.001
	Total effect	-0.0601	-0.3435(-0.5099, -0.177)	<0.001	-0.0617	-0.3524(-0.5234, -0.1815)	<0.001	-0.0593	-0.3390(-0.5098, -0.1683)	<0.001	-0.0602	-0.3429(-0.5133, -0.1724)	<0.001	-0.0471	-0.2682(-0.4388, -0.0976)	0.0021
**Path2**	Menarche age→BMI	0.1085	-0.2051(-0.2601, -0.1502)	<0.001	-0.0731	-0.1381(-0.1936, -0.0826)	<0.001	-0.0712	-0.1346(-0.1898, -0.0793)	<0.001	-0.0711	-0.1342(-0.1887, -0.0797)	<0.001	-0.0473	-0.0892(-0.1416, -0.0369)	<0.001
	Menarche age→SBP	-0.0004	-0.0046(-0.3619,0.3526)	0.9797	-0.0477	-0.585(-0.9350, -0.2351)	0.0011	-0.0466	-0.5711(-0.9209, -0.2213)	0.0014	-0.0486	-0.5957(-0.9468, -0.2446)	<0.001	-0.0446	-0.5479(-0.9025, -0.1932)	0.0025
	BMI→SBP	0.1289	0.8359(0.6469,1.0249)	<0.001	0.1685	1.0927(0.9088, 1.2766)	<0.001	0.1682	1.0905(0.9058,1.2753)	<0.001	0.1585	1.0286(0.8395,1.2176)	<0.001	0.1441	0.9366(0.7361,1.1371)	<0.001
	Total effect	-0.0144	-0.1761(-0.5342,0.182)	0.3350	-0.0601	-0.736(-1.0902, -0.3817)	<0.001	-0.0586	-0.7178(-1.0719, -0.3638)	<0.001	-0.0599	-0.7337(-1.0883, -0.3791)	<0.001	-0.0515	-0.6314(-0.9889, -0.2739)	<0.001
**Path3**	Menarche age→WC	-0.0837	-0.4928(-0.6642, -0.3213)	<0.001	-0.0910	-0.5355(-0.7107, -0.3602)	<0.001	-0.0896	-0.5275(-0.7024, -0.3526)	<0.001	-0.0898	-0.5285(-0.7026, -0.3545)	<0.001	-0.0696	-0.4085(-0.5778, -0.2392)	<0.001
	Menarche age→DBP	-0.0454	-0.2589(-0.4232, -0.0945)	0.0020	-0.0456	-0.26(-0.4288, -0.0912)	0.0025	-0.0437	-0.2491(-0.4178, -0.0805)	0.0038	-0.0452	-0.2571(-0.4257, -0.0884)	0.0028	-0.0373	-0.2118(-0.3811, -0.0424)	0.0143
	WC→DBP	0.1789	0.1733(0.1454, 0.2013)	<0.001	-0.1810	0.1754(0.1473, 0.2034)	<0.001	0.1787	0.1732(0.1451, 0.2013)	<0.001	0.1705	0.1647(0.1363, 0.1931)	<0.001	0.1445	0.1399(0.1104, 0.1695)	<0.001
	Total effect	-0.0604	-0.3443(-0.5107, -0.1779)	<0.001	-0.0620	-0.3539(-0.5248, -0.183)	<0.001	-0.0597	-0.3405(-0.5112, -0.1698)	<0.001	-0.0606	-0.3441(-0.5145, -0.1737)	<0.001	-0.0474	-0.2689(-0.4395, -0.0984)	0.0020
**Path4**	Menarche age→WC	-0.0849	-0.4944(-0.6658, -0.323)	<0.001	-0.0914	-0.5376(-0.7129, -0.3624)	<0.001	-0.0901	-0.5299(-0.7048, -0.355)	<0.001	-0.0904	-0.531(-0.7051, -0.357)	<0.001	-0.0702	-0.411(-0.5803, -0.2417)	<0.001
	Menarche age→SBP	0.0004	0.0044(-0.3439, 0.3526)	0.9804	-0.0468	-0.5643(-0.9094, -0.2192)	0.0014	-0.0459	-0.5533(-0.8983, -0.2082)	0.0017	-0.0481	-0.5792(-0.9255, -0.233)	0.0010	-0.0433	-0.5228(-0.8721, -0.1735)	0.0034
	WC→SBP	0.1772	0.3632(0.3040, 0.4224)	<0.001	0.1574	0.3226(0.2652,0.38)	<0.001	0.1538	0.3151(0.2576, 0.3727)	<0.001	0.1436	0.2942(0.2358, 0.3526)	<0.001	0.1290	0.2655(0.2045,0.3266)	<0.001
	Total effect	-0.0145	-0.1752(-0.5277, 0.1773)	0.3300	-0.0612	-0.7377(-1.086, -0.3894)	<0.001	-0.0598	-0.7202(-1.0683, -0.3722)	<0.001	-0.0611	-0.7354(-1.084, -0.3869)	<0.001	-0.0524	-0.6320(-0.9832, -0.2807)	<0.001

CVDs: cardiovascular diseases; LDL-C: low-density lipoprotein cholesterol; HDL-C: high-density lipoprotein cholesterol; TG: triglycerides; HbA1c: Hemoglobin A1C; Scr: serum creatinine; SUA: serum uric acid; BMI: body mass index; WC: waist circle; DBP: diastolic blood pressure; SBP: systolic blood pressure; Un-standardized: B, standardized coefficients, β.
Path1: Mediation effect of BMI on the relationship between age at menarche and DBP.
Path2: Mediation effect of BMI on the relationship between age at menarche and SBP.
Path3: Mediation effect of WC on the relationship between age at menarche and DBP.
Path4: Mediation effect of WC on the relationship between age at menarche and SBP.
[a] **Unadjusted;**
[b] **Adjusted for** age, educational attainment, marital status, region;
[c] **Adjusted for** age, educational attainment, marital status, region, cigarette smoking, alcohol consumption, eating habits, entertainment, the experience of a traumatic event, physical activity;
[d] **Adjusted for** age, educational attainment, marital status, region, cigarette smoking, alcohol consumption, eating habits, entertainment, the experience of a traumatic event, physical activity, history of CVDs, hepatitis history, history of antilipidemic medication, history of antidiabetic medication;
[e] **Adjusted for** age, educational attainment, marital status, region, cigarette smoking, alcohol consumption, eating habits, entertainment, the experience of a traumatic event, physical activity, history of CVDs, hepatitis history, history of antilipidemic medication, history of antidiabetic medication, LDL-C, HDL-C, TG, HbA1c, Scr, SUA.

For peer review only

DISCUSSION

In the study, we attempted to explore the association between age of menarche and BP and examined the moderating and mediating effect of obesity parameters (BMI and WC) on the association. We have found that the interaction term of obesity parameters*age of menarche was not significant for predicting BP. It was also important to note that WC and BMI partly mediated the association between age of menarche and BP. The results of our study were partly constant with most previous studies. And so previous studies had only focused on the relationship between earlier biological maturation and health outcomes in late adolescence and adulthood. Most of the previous studies meant exactly that: earlier biological maturation was at a higher risk for health outcomes in late adolescence and adulthood. Furthermore, most previous studies found that early biological maturation (age of menarche) or puberty was significantly related to obesity parameters[34-38], hypertension[38-41], and metabolic syndrome[42-50]. In general, our results were mostly consistent with the results of previous studies that suggest that earlier biological maturation is a risk factor for several negative health outcomes in late adolescence and adulthood.

Our study suggested the likelihood of a dose-response relationship between age of menarche and obesity parameters (BMI and WC). The relationship could be identified by the fact that early-maturing girls already have more adiposity before menarche, given that fatty tissue, through leptin release, has a crucial role in the initiation of the biological maturation process[51 52]. Early maturing girls could maintain their higher fatty tissue in adulthood[53]. Another mechanism may also be associated with the time of the maturation process. It has been reported that early maturing girls could experience a more accelerated maturation process than late maturing ones. Thus, more significant interference with biological maturation in girls can lead to increased more fatty tissue[54]. We also found a significantly negative relationship between age at menarche and BP in mid-aged and elderly Chinese women. However, this association did not exist between the age of menarche and SBP before adjusting the related confounders. This result is consistent with previous findings[55-63] regarding the age of menarche as a cardiovascular risk factor. The weak association between age at menarche and BP in adulthood could, in part, be explained by the fact that early matures were younger than women with late menarche. This result could be an expression of the fact that BP is an outcome with a higher latency period than obesity. Moreover, among women who already entered menopause in our study, the effects of menarche could be even weaker, as results suggest, and obesity could have more effect on BP. We also found that women with a higher age of menarche had higher HDL-C

For peer review only - http://bmjopen.bmj.com/site/about/guidelines.xhtml

level but had lower DBP level. The results of the current study were quite consistent with those of [22] Feng Y, *et al.* Increased body fat, especially abdominal fat, partially explained the increased insulin resistance and dyslipidemia in women with early menarche age. Therefore, obesity in adolescence, sociodemographic, or even behavioral factors through life (including age, educational attainment, marital status, region, smoking status, alcohol consumption, eating habits, entertainment, the experience of a traumatic event, physical activity) were potential mediators to target when interventions for BP control and management.

Though so many studies have explored the association analysis between the age of menarche or obesity parameters and BP, there were only two studies[64 65] that explored the mediating effect of obesity parameters on the relation between age of menarche and BP. Zhang L, *et al.* [64] found that the relationship between age of menarche and DBP was partly mediated by WC. In contrast, the relationship between age of menarche and SBP was fully mediated by WC in women. Werneck AO, *et al.* [65] showed that women with late menarche are less likely to have a risk of hypertension, and BMI was an important mediator of the age at the menarche-hypertension association. Interestingly, we found that obesity parameters partly mediated the relationship between the age of menarche and BP in women. The difference between our research and others may due to the different populations, different definitions of early and later menarche, and different confounding variables by controlling. The individuals in our study were mid-aged and elderly Chinese women, where the mean age at recruitment and age of menarche were older than in the Werneck AO, *et al*' s study[65], and the level of socio-economic development also made some contribution to ontogenetic development. In addition, other studies and a meta-analysis[55 57 63 66-68] reported an inverse association between early age of menarche and hypertension, which was not observed in Zhang L, *et al*' study[64]. They found no direct relationship between the age of menarche and DBP, and we have found a direct relationship between the age of menarche and DBP. The participants were similar in China and its socio-economic background, this phenomenon could be explained by the cumulative effect, which showed a significantly negative association between age of menarche and DBP over life-span.

There were several limitations to the study. The main limitations of our research are related to the cross-sectional study and the self-reported method used for the assessment of age at menarche and most related confounders. BP in our study was measured at home by professionally trained volunteers, and the next step is the use of clinical BP and ambulatory blood pressure. However, this is the first

For peer review only - http://bmjopen.bmj.com/site/about/guidelines.xhtml

large population study to examine the moderation between the age of menarche and obesity parameters in predicting BP in middle-aged and elderly Chinese, as well as the mediation effects of obesity parameters on the relationship between age of menarche and BP. Moreover, a significant strength of the study is a large sample of 4513 middle-aged and older Chinese. The results presented in this article represent the baseline data that could be explored further in prospective cohort studies.

In sum, the interaction term of obesity parameters*age of menarche was not significant for predicting either DBP or SBP in women. Moreover, obesity parameters partly mediated the relationship between the age of menarche and BP.

CONCLUSIONS

The interaction term of obesity parameters*age of menarche was not significant for predicting either DBP or SBP in women. Moreover, Obesity parameters partly mediated the relationship between age of menarche and BP.

Author affiliations

[1] Department of Internal Medicine Nursing, School of Nursing, Wannan Medical College, 22 Wenchang West Road, Higher Education Park, Wuhu City, An Hui Province, P.R.China

[2] Obstetrics and Gynecology Nursing, School of Nursing, Wannan Medical College, 22 Wenchang West Road, Higher Education Park, Wuhu City, An Hui Province, P.R.China

[3]Department of Pediatric Nursing, School of Nursing, Wannan Medical College, 22 Wenchang West Road, Higher Education Park, Wuhu City, An Hui Province, P.R.China

[4] Department of Surgical Nursing, School of Nursing, Wannan Medical College, 22 Wenchang West Road, Higher Education Park, Wuhu City, An Hui Province, P.R.China

[5]Department of Emergency and Critical Care Nursing, School of Nursing, Wannan Medical College, 22 Wenchang West Road, Higher Education Park, Wuhu City, An Hui Province, P.R.China

[6]Rehabilitation Nursing, School of Nursing, Wanna Medical college, 22 Wenchang West Road, Higher Education Park, Wuhu City, An Hui Province, P.R.China

[7]Department of nursing, Yijishan Hospital, the First Affiliated Hospital of Wannan Medical College, Zheshan West Road, Yijishan District, Wuhu City, Anhui Province

Abbreviations

BMI, body mass index; BP, blood pressure; Confidence intervals, CIs; CDC, Centers for Disease Control and Prevention; CHARLS, China Health and Retirement Longitudinal Study; CVDs,

For peer review only

For peer review only - http://bmjopen.bmj.com/site/about/guidelines.xhtml

cardiovascular diseases; DBP, diastolic blood pressure; HDL, high-density lipoprotein; Hemoglobin A1C, HbA1c; LDL, low-density lipoprotein; M, mean; NSFC, The National Natural Science Foundation of China; National Institute on Aging, NIA; SBP, systolic blood pressure; SUA, serum uric acid; standard error, SE; unstandardized, B; standardized coefficients, β; Scr, serum creatinine; SD, standard deviation; TG, triglycerides; urinalysis, UA; World Bank, WB; waist circle, WC; World Health Organization, WHO.

Acknowledgements

We are grateful to the China Center for Economic Research, and we also thank all participants for their contribution and members of the research for collecting the data.

Author contributions

Conceived and designed the research: LZ. Wrote the paper: LZ. Analyzed the data: LZ. Revised the paper: LZ, L Y, C-z W, TY, Dm Z, H-h W, JL, Y-x L, LS, X-p L, YH, H-y C, and Y-z L.

Funding

CHARLS was supported by the NSFC (70910107022, 71130002) and National Institute on Aging (R03-TW008358-01; R01-AG037031-03S1), and World Bank (7159234) and the publication fee was supported by the Support Program for Outstanding Young Talents from the Universities and Colleges of Anhui Province for Lin Zhang(gxyqZD2021118).

Disclosure

The authors declare that there is no potential conflict of interest relevant to the research.

Patient consent

Completion of all authors declaration and consent to publish form is required.

Ethics approval and consent to participate

Ethics approval for the research was granted by the Ethics Review Committee of Peking University, and all participants provided informed consent. Approval for this study was given by the medical ethics committee of Wannan medical college (approval number 2021–3).

Data sharing

Data sharing statement Extra data can be accessed via http://charls.pku.edu.cn/pages/data/111/zh-cn.html.

For peer review only

References

1. Tian S, Xu Y, Dong H. The effect of metabolic health and obesity phenotypes on risk of hypertension: A nationwide population-based study using 5 representative definitions of metabolic health. Medicine (Baltimore) 2018;**97**(38):e12425 doi: 10.1097/MD.0000000000012425[published Online First: Epub Date]|.
2. Abdulsalam S, Olugbenga-Bello A, Olarewaju O, et al. Sociodemographic correlates of modifiable risk factors for hypertension in a rural local government area of oyo state South west Nigeria. Int J Hypertens 2014;**2014**:842028 doi: 10.1155/2014/842028[published Online First: Epub Date]|.
3. Ke L, Ho J, Feng J, et al. Modifiable risk factors including sunlight exposure and fish consumption are associated with risk of hypertension in a large representative population from Macau. J Steroid Biochem Mol Biol 2014;**144 Pt A**:152-5 doi: 10.1016/j.jsbmb.2013.10.019[published Online First: Epub Date]|.
4. Ibekwe R. Modifiable Risk factors of Hypertension and Socio-demographic Profile in Oghara, Delta State; Prevalence and Correlates. Ann Med Health Sci Res 2015;**5**(1):71-7 doi: 10.4103/2141-9248.149793[published Online First: Epub Date]|.
5. Pilakkadavath Z, Shaffi M. Modifiable risk factors of hypertension: A hospital-based case-control study from Kerala, India. J Family Med Prim Care 2016;**5**(1):114-9 doi: 10.4103/2249-4863.184634[published Online First: Epub Date]|.
6. Mamudu HM, Paul TK, Wang L, et al. Association Between Multiple Modifiable Risk Factors of Cardiovascular Disease and Hypertension among Asymptomatic Patients in Central Appalachia. South Med J 2017;**110**(2):90-96 doi: 10.14423/SMJ.0000000000000602[published Online First: Epub Date]|.
7. Collaboration NCDRF. Worldwide trends in blood pressure from 1975 to 2015: a pooled analysis of 1479 population-based measurement studies with 19.1 million participants. Lancet 2017;**389**(10064):37-55 doi: 10.1016/S0140-6736(16)31919-5[published Online First: Epub Date]|.
8. Bromfield S, Muntner P. High blood pressure: the leading global burden of disease risk factor and the need for worldwide prevention programs. Curr Hypertens Rep 2013;**15**(3):134-6 doi: 10.1007/s11906-013-0340-9[published Online First: Epub Date]|.
9. Mahajan S, Zhang D, He S, et al. Prevalence, Awareness, and Treatment of Isolated Diastolic Hypertension: Insights From the China PEACE Million Persons Project. J Am Heart Assoc 2019;**8**(19):e012954 doi: 10.1161/JAHA.119.012954[published Online First: Epub Date]|.
10. Cloutier L, Morris D, Bruneau J, et al. World Health Organization celebrates World Health Day, April 7,2013--focusing on hypertension. Canadian journal of cardiovascular nursing = Journal canadien en soins infirmiers cardio-vasculaires 2013;**23**(2):9-11
11. Webb MS. Stress management strategies in hypertension control. Ethnicity & disease 2002;**12**(4):S3-95-100
12. Kotchen TA. The search for strategies to control hypertension. Circulation 2010;**122**(12):1141-3 doi: 10.1161/CIRCULATIONAHA.110.978759[published Online First: Epub Date]|.
13. Mancia G, Rea F, Cuspidi C, et al. Blood pressure control in hypertension. Pros and cons of available treatment strategies. Journal of hypertension 2017;**35**(2):225-33 doi: 10.1097/HJH.0000000000001181[published Online First: Epub Date]|.
14. Werneck AO, Silva DR, Souza MF, et al. Correlates of Blood Pressure According to Early, On Time, and Late Maturation in Adolescents. Journal of clinical hypertension 2016;**18**(5):424-30 doi: 10.1111/jch.12699[published Online First: Epub Date]|.
15. Widen E, Silventoinen K, Sovio U, et al. Pubertal timing and growth influences cardiometabolic risk factors in adult males and females. Diabetes care 2012;**35**(4):850-6 doi: 10.2337/dc11-1365[published Online First: Epub Date]|.
16. Mueller NT, Duncan BB, Barreto SM, et al. Earlier age at menarche is associated with higher diabetes risk and cardiometabolic disease risk factors in Brazilian adults: Brazilian Longitudinal Study of Adult Health (ELSA-Brasil). Cardiovasc Diabetol 2014;**13**:22 doi: 10.1186/1475-2840-13-22[published Online First: Epub Date]|.

For peer review only - http://bmjopen.bmj.com/site/about/guidelines.xhtml

17. Dreyfus J, Jacobs DR, Jr., Mueller N, et al. Age at Menarche and Cardiometabolic Risk in Adulthood: The Coronary Artery Risk Development in Young Adults Study. J Pediatr 2015;**167**(2):344-52 e1 doi: 10.1016/j.jpeds.2015.04.032[published Online First: Epub Date]|.

18. Cao M, Cui B. Negative Effects of Age at Menarche on Risk of Cardiometabolic Diseases in Adulthood: A Mendelian Randomization Study. The Journal of clinical endocrinology and metabolism 2020;**105**(2) doi: 10.1210/clinem/dgz071[published Online First: Epub Date]|.

19. Power C, Lake JK, Cole TJ. Body mass index and height from childhood to adulthood in the 1958 British born cohort. The American journal of clinical nutrition 1997;**66**(5):1094-101 doi: 10.1093/ajcn/66.5.1094[published Online First: Epub Date]|.

20. Adair LS, Gordon-Larsen P. Maturational timing and overweight prevalence in US adolescent girls. American journal of public health 2001;**91**(4):642-4 doi: 10.2105/ajph.91.4.642[published Online First: Epub Date]|.

21. Must A, Naumova EN, Phillips SM, et al. Childhood overweight and maturational timing in the development of adult overweight and fatness: the Newton Girls Study and its follow-up. Pediatrics 2005;**116**(3):620-7 doi: 10.1542/peds.2004-1604[published Online First: Epub Date]|.

[dataset] 22. Zhao Y, Hu Y, Smith JP, et al. Cohort profile: the China Health and Retirement Longitudinal Study (CHARLS). International journal of epidemiology 2014;**43**(1):61-8 doi: 10.1093/ije/dys203[published Online First: Epub Date]|.

23. Li C, Liu T, Sun W, et al. Prevalence and risk factors of arthritis in a middle-aged and older Chinese population: the China health and retirement longitudinal study. Rheumatology 2015;**54**(4):697-706 doi: 10.1093/rheumatology/keu391[published Online First: Epub Date]|.

24. Zhang L, Yang L, Wang C, et al. Individual and combined association analysis of famine exposure and serum uric acid with hypertension in the mid-aged and older adult: a population-based cross-sectional study. BioMed research international 2021;**21**(1):420 doi: 10.1155/2021/5594718

10.1186/s12872-021-02230-z[published Online First: Epub Date]|.

25. Zhang L, Yang L, Wang C, et al. Combined Effect of Famine Exposure and Obesity Parameters on Hypertension in the Midaged and Older Adult: A Population-Based Cross-Sectional Study. 2021;**2021**:5594718 doi: 10.1155/2021/5594718[published Online First: Epub Date]|.

26. Zhang L, Li JL, Zhang LL, et al. Body mass index and serum uric acid level: Individual and combined effects on blood pressure in middle-aged and older individuals in China. Medicine (Baltimore) 2020;**99**(9):e19418 doi: 10.1097/MD.0000000000019418[published Online First: Epub Date]|.

27. Zhang L, Li JL, Zhang LL, et al. Relationship between adiposity parameters and cognition: the "fat and jolly" hypothesis in middle-aged and elderly people in China. Medicine 2019;**98**(10) doi: ARTN e14747

10.1097/MD.0000000000014747[published Online First: Epub Date]|.

28. Zhang L, Li JL, Zhang LL, et al. Association and Interaction Analysis of Body Mass Index and Triglycerides Level with Blood Pressure in Elderly Individuals in China. BioMed research international 2018;**2018**:8934534 doi: 10.1155/2018/8934534[published Online First: Epub Date]|.

29. Zhang L, Li JL, Zhang LL, et al. No association between C-reactive protein and depressive symptoms among the middle-aged and elderly in China Evidence from the China Health and Retirement Longitudinal Study. Medicine 2018;**97**(38) doi: ARTN e12352

10.1097/MD.0000000000012352[published Online First: Epub Date]|.

30. Zhang L, Liu K, Li H, et al. Relationship between body mass index and depressive symptoms: the "fat and jolly" hypothesis for the middle-aged and elderly in China. BMC public health 2016;**16**(1):1201 doi: 10.1186/s12889-016-3864-5[published Online First: Epub Date]|.

31. Hayes AF, Preacher KJ. Quantifying and Testing Indirect Effects in Simple Mediation Models When the Constituent Paths Are Nonlinear. Multivariate Behav Res 2010;**45**(4):627-60 doi: 10.1080/00273171.2010.498290[published Online First: Epub Date]|.

For peer review only

For peer review only - http://bmjopen.bmj.com/site/about/guidelines.xhtml

32. Hayes AF, Preacher KJ. Statistical mediation analysis with a multicategorical independent variable. Br J Math Stat Psychol 2014;**67**(3):451-70 doi: 10.1111/bmsp.12028[published Online First: Epub Date]|.
33. Hayes AF. An Index and Test of Linear Moderated Mediation. Multivariate Behav Res 2015;**50**(1):1-22 doi: 10.1080/00273171.2014.962683[published Online First: Epub Date]|.
34. Shrestha A, Olsen J, Ramlau-Hansen CH, et al. Obesity and age at menarche. Fertil Steril 2011;**95**(8):2732-4 doi: 10.1016/j.fertnstert.2011.02.020[published Online First: Epub Date]|.
35. Bralic I, Tahirovic H, Matanic D, et al. Association of early menarche age and overweight/obesity. J Pediatr Endocrinol Metab 2012;**25**(1-2):57-62 doi: 10.1515/jpem-2011-0277[published Online First: Epub Date]|.
36. Leitao RB, Rodrigues LP, Neves L, et al. Development of adiposity, obesity and age at menarche: an 8-year follow-up study in Portuguese schoolgirls. International journal of adolescent medicine and health 2013;**25**(1):55-63 doi: 10.1515/ijamh-2013-0007[published Online First: Epub Date]|.
37. Gallicchio L, Flaws JA, Smith RL. Age at menarche, androgen concentrations, and midlife obesity: findings from the Midlife Women's Health Study. Menopause 2016;**23**(11):1182-88 doi: 10.1097/GME.0000000000000691[published Online First: Epub Date]|.
38. Liu G, Yang Y, Huang W, et al. Association of age at menarche with obesity and hypertension among southwestern Chinese women: a new finding. Menopause 2018;**25**(5):546-53 doi: 10.1097/GME.0000000000001027[published Online First: Epub Date]|.
39. Zhou W, Wang T, Zhu L, et al. Association between Age at Menarche and Hypertension among Females in Southern China: A Cross-Sectional Study. Int J Hypertens 2019;**2019**:9473182 doi: 10.1155/2019/9473182[published Online First: Epub Date]|.
40. Shen L, Wang L, Hu Y, et al. Associations of the ages at menarche and menopause with blood pressure and hypertension among middle-aged and older Chinese women: a cross-sectional analysis of the baseline data of the China Health and Retirement Longitudinal Study. Hypertension research : official journal of the Japanese Society of Hypertension 2019;**42**(5):730-38 doi: 10.1038/s41440-019-0235-5[published Online First: Epub Date]|.
41. Guo L, Peng C, Xu H, et al. Age at menarche and prevention of hypertension through lifestyle in young Chinese adult women: result from project ELEFANT. BMC Womens Health 2018;**18**(1):182 doi: 10.1186/s12905-018-0677-y[published Online First: Epub Date]|.
42. Stockl D, Meisinger C, Peters A, et al. Age at menarche and its association with the metabolic syndrome and its components: results from the KORA F4 study. PloS one 2011;**6**(10):e26076 doi: 10.1371/journal.pone.0026076[published Online First: Epub Date]|.
43. Chang CJ, Lai MM, Lin CC, et al. Age at menarche and its association with the metabolic syndrome in Taiwan. Obesity research & clinical practice 2016;**10 Suppl 1**:S26-S34 doi: 10.1016/j.orcp.2015.10.003[published Online First: Epub Date]|.
44. Heys M, Schooling CM, Jiang C, et al. Age of menarche and the metabolic syndrome in China. Epidemiology 2007;**18**(6):740-6 doi: 10.1097/EDE.0b013e3181567faf[published Online First: Epub Date]|.
45. Yoo S, Hwang YS. Age-Group Related Cohort Effects on the Association between Age at Menarche and Metabolic Syndrome among Korean Premenopausal Women. Korean journal of family medicine 2019;**40**(4):280-81 doi: 10.4082/kjfm.19.0045[published Online First: Epub Date]|.
46. Akter S, Jesmin S, Islam M, et al. Association of age at menarche with metabolic syndrome and its components in rural Bangladeshi women. Nutr Metab (Lond) 2012;**9**(1):99 doi: 10.1186/1743-7075-9-99[published Online First: Epub Date]|.
47. Glueck CJ, Morrison JA, Wang P, et al. Early and late menarche are associated with oligomenorrhea and predict metabolic syndrome 26 years later. Metabolism: clinical and experimental 2013;**62**(11):1597-606 doi: 10.1016/j.metabol.2013.07.005[published Online First: Epub Date]|.
48. Kim Y, Je Y. Early Menarche and Risk of Metabolic Syndrome: A Systematic Review and Meta-Analysis. Journal of

For peer review only - http://bmjopen.bmj.com/site/about/guidelines.xhtml

women's health 2019;**28**(1):77-86 doi: 10.1089/jwh.2018.6998[published Online First: Epub Date]|.

49. Farahmand M, Ramezani Tehrani F, Behboudi Gandevani S, et al. Is There any Association between Age at Menarche and Risk of Metabolic Syndrome? The Tehran Lipid & Glucose Study. Arch Iran Med 2019;**22**(9):495-500

50. Hwang YS, Park EJ, Choi JG, et al. Relationship between Age at Menarche and Metabolic Syndrome in Premenopausal Women: Korea National Health and Nutrition Examination Survey 2013-2014. Korean journal of family medicine 2018;**39**(5):300-06 doi: 10.4082/kjfm.17.0022[published Online First: Epub Date]|.

51. Ahmed ML, Ong KK, Dunger DB. Childhood obesity and the timing of puberty. Trends Endocrinol Metab 2009;**20**(5):237-42 doi: 10.1016/j.tem.2009.02.004[published Online First: Epub Date]|.

52. Jasik CB, Lustig RH. Adolescent obesity and puberty: the "perfect storm". Ann N Y Acad Sci 2008;**1135**:265-79 doi: 10.1196/annals.1429.009[published Online First: Epub Date]|.

53. Garn SM. Continuities and changes in fatness from infancy through adulthood. Curr Probl Pediatr 1985;**15**(2):1-47 doi: 10.1016/0045-9380(85)90015-5[published Online First: Epub Date]|.

54. van Lenthe FJ, Kemper CG, van Mechelen W. Rapid maturation in adolescence results in greater obesity in adulthood: the Amsterdam Growth and Health Study. The American journal of clinical nutrition 1996;**64**(1):18-24 doi: 10.1093/ajcn/64.1.18[published Online First: Epub Date]|.

55. Remsberg KE, Demerath EW, Schubert CM, et al. Early menarche and the development of cardiovascular disease risk factors in adolescent girls: the Fels Longitudinal Study. The Journal of clinical endocrinology and metabolism 2005;**90**(5):2718-24 doi: 10.1210/jc.2004-1991[published Online First: Epub Date]|.

56. Feng Y, Hong X, Wilker E, et al. Effects of age at menarche, reproductive years, and menopause on metabolic risk factors for cardiovascular diseases. Atherosclerosis 2008;**196**(2):590-7 doi: 10.1016/j.atherosclerosis.2007.06.016[published Online First: Epub Date]|.

57. Won JC, Hong JW, Noh JH, et al. Association Between Age at Menarche and Risk Factors for Cardiovascular Diseases in Korean Women: The 2010 to 2013 Korea National Health and Nutrition Examination Survey. Medicine (Baltimore) 2016;**95**(18):e3580 doi: 10.1097/MD.0000000000003580[published Online First: Epub Date]|.

58. Au Yeung SL, Jiang C, Cheng KK, et al. Age at menarche and cardiovascular risk factors using Mendelian randomization in the Guangzhou Biobank Cohort Study. Preventive medicine 2017;**101**:142-48 doi: 10.1016/j.ypmed.2017.06.006[published Online First: Epub Date]|.

59. Ley SH, Li Y, Tobias DK, et al. Duration of Reproductive Life Span, Age at Menarche, and Age at Menopause Are Associated With Risk of Cardiovascular Disease in Women. J Am Heart Assoc 2017;**6**(11) doi: 10.1161/JAHA.117.006713[published Online First: Epub Date]|.

60. Luijken J, van der Schouw YT, Mensink D, et al. Association between age at menarche and cardiovascular disease: A systematic review on risk and potential mechanisms. Maturitas 2017;**104**:96-116 doi: 10.1016/j.maturitas.2017.07.009[published Online First: Epub Date]|.

61. Yang L, Li L, Millwood IY, et al. Age at menarche and risk of major cardiovascular diseases: Evidence of birth cohort effects from a prospective study of 300,000 Chinese women. International journal of cardiology 2017;**227**:497-502 doi: 10.1016/j.ijcard.2016.10.115[published Online First: Epub Date]|.

62. Chen X, Liu Y, Sun X, et al. Age at menarche and risk of all-cause and cardiovascular mortality: a systematic review and dose-response meta-analysis. Menopause 2018;**26**(6):670-76 doi: 10.1097/GME.0000000000001289[published Online First: Epub Date]|.

63. Lee JJ, Cook-Wiens G, Johnson BD, et al. Age at Menarche and Risk of Cardiovascular Disease Outcomes: Findings From the National Heart Lung and Blood Institute-Sponsored Women's Ischemia Syndrome Evaluation. J Am Heart Assoc 2019;**8**(12):e012406 doi: 10.1161/JAHA.119.012406[published Online First: Epub Date]|.

64. Zhang L, Li Y, Zhou W, et al. Mediation effect of BMI on the relationship between age at menarche and hypertension: The Henan Rural Cohort Study. Journal of human hypertension 2019 doi: 10.1038/s41371-019-0247-2[published Online First: Epub Date]|.

65. Werneck AO, Oyeyemi AL, Cyrino ES, et al. Association between age at menarche and blood pressure in adulthood:

For peer review only - http://bmjopen.bmj.com/site/about/guidelines.xhtml

is obesity an important mediator? Hypertension research : official journal of the Japanese Society of Hypertension 2018;**41**(10):856-64 doi: 10.1038/s41440-018-0079-4[published Online First: Epub Date]|.

66. Lakshman R, Forouhi NG, Sharp SJ, et al. Early age at menarche associated with cardiovascular disease and mortality. The Journal of clinical endocrinology and metabolism 2009;**94**(12):4953-60 doi: 10.1210/jc.2009-1789[published Online First: Epub Date]|.
67. Charalampopoulos D, McLoughlin A, Elks CE, et al. Age at menarche and risks of all-cause and cardiovascular death: a systematic review and meta-analysis. American journal of epidemiology 2014;**180**(1):29-40 doi: 10.1093/aje/kwu113[published Online First: Epub Date]|.
68. Zheng Y, Zhang G, Chen Z, et al. Association between Age at Menarche and Cardiovascular Disease Risk Factors in China: A Large Population-Based Investigation. Cardiorenal Med 2016;**6**(4):307-16 doi: 10.1159/000445506[published Online First: Epub Date]|.

For peer review only

For peer review only - http://bmjopen.bmj.com/site/about/guidelines.xhtml

Fig. 1 Selection of participants

For peer review only

For peer review only - http://bmjopen.bmj.com/site/about/guidelines.xhtml

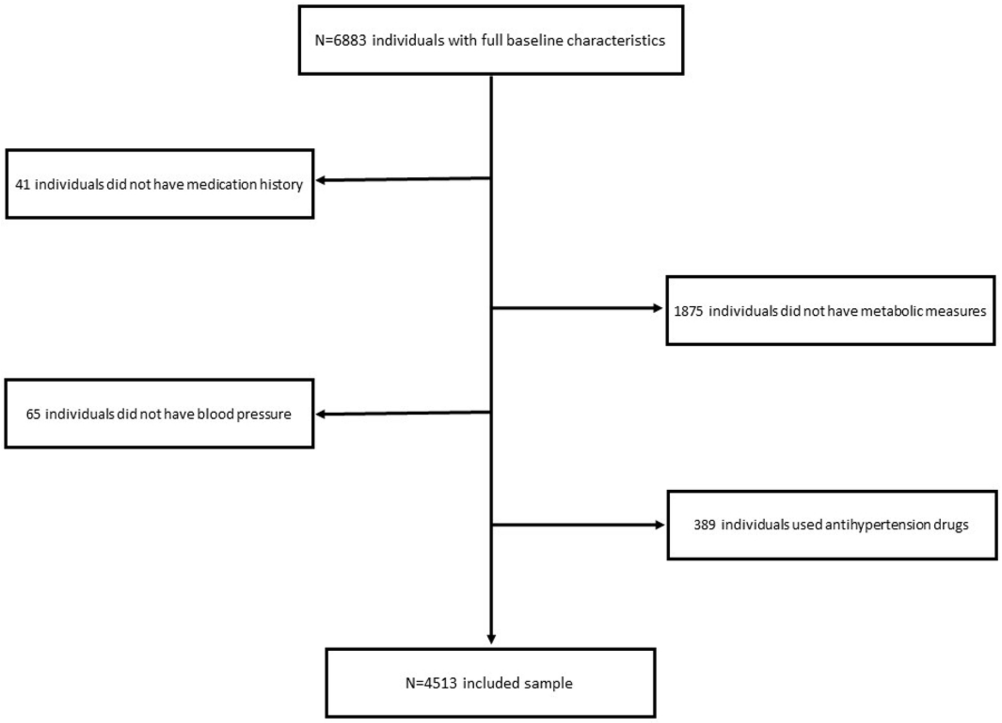

90x90mm (300 x 300 DPI)

STROBE 2007 (v4) Statement—Checklist of items that should be included in reports of *cross-sectional studies*

Section/Topic	Item #	Recommendation	Reported on page #
**Title and abstract**	1	(*a*) Indicate the study's design with a commonly used term in the title or the abstract	1
		(*b*) Provide in the abstract an informative and balanced summary of what was done and what was found	2-3
**Introduction**			
Background/rationale	2	Explain the scientific background and rationale for the investigation being reported	4-5
Objectives	3	State specific objectives, including any prespecified hypotheses	5
**Methods**			
Study design	4	Present key elements of study design early in the paper	6
Setting	5	Describe the setting, locations, and relevant dates, including periods of recruitment, exposure, follow-up, and data collection	6
Participants	6	(*a*) Give the eligibility criteria, and the sources and methods of selection of participants	7
Variables	7	Clearly define all outcomes, exposures, predictors, potential confounders, and effect modifiers. Give diagnostic criteria, if applicable	7-8
Data sources/ measurement	8*	For each variable of interest, give sources of data and details of methods of assessment (measurement). Describe comparability of assessment methods if there is more than one group	8
Bias	9	Describe any efforts to address potential sources of bias	*8*
Study size	10	Explain how the study size was arrived at	7
Quantitative variables	11	Explain how quantitative variables were handled in the analyses. If applicable, describe which groupings were chosen and why	7
Statistical methods	12	(*a*) Describe all statistical methods, including those used to control for confounding	7-8
		(*b*) Describe any methods used to examine subgroups and interactions	7-8
		(*c*) Explain how missing data were addressed	7-8
		(*d*) If applicable, describe analytical methods taking account of sampling strategy	7-8
		(*e*) Describe any sensitivity analyses	7-8
**Results**			

For peer review only

Participants	13*	(a) Report numbers of individuals at each stage of study—eg numbers potentially eligible, examined for eligibility, confirmed eligible, included in the study, completing follow-up, and analysed	9-10
		(b) Give reasons for non-participation at each stage	9-10
		(c) Consider use of a flow diagram	9-10
Descriptive data	14*	(a) Give characteristics of study participants (eg demographic, clinical, social) and information on exposures and potential confounders	10-13
		(b) Indicate number of participants with missing data for each variable of interest	10-13
Outcome data	15*	Report numbers of outcome events or summary measures	10-13
Main results	16	(*a*) Give unadjusted estimates and, if applicable, confounder-adjusted estimates and their precision (eg, 95% confidence interval). Make clear which confounders were adjusted for and why they were included	13-16
		(*b*) Report category boundaries when continuous variables were categorized	13-16
		(*c*) If relevant, consider translating estimates of relative risk into absolute risk for a meaningful time period	13-16
Other analyses	17	Report other analyses done—eg analyses of subgroups and interactions, and sensitivity analyses	
**Discussion**			
Key results	18	Summarise key results with reference to study objectives	17-19
Limitations	19	Discuss limitations of the study, taking into account sources of potential bias or imprecision. Discuss both direction and magnitude of any potential bias	17-19
Interpretation	20	Give a cautious overall interpretation of results considering objectives, limitations, multiplicity of analyses, results from similar studies, and other relevant evidence	17-19
Generalisability	21	Discuss the generalisability (external validity) of the study results	17-19
**Other information**			
Funding	22	Give the source of funding and the role of the funders for the present study and, if applicable, for the original study on which the present article is based	20

*Give information separately for cases and controls in case-control studies and, if applicable, for exposed and unexposed groups in cohort and cross-sectional studies.

Note: An Explanation and Elaboration article discusses each checklist item and gives methodological background and published examples of transparent reporting. The STROBE checklist is best used in conjunction with this article (freely available on the Web sites of PLoS Medicine at http://www.plosmedicine.org/, Annals of Internal Medicine at http://www.annals.org/, and Epidemiology at http://www.epidem.com/). Information on the STROBE Initiative is available at www.strobe-statement.org.

For peer review only
